# Genetic disruption of mitochondrial dynamics and stasis leads to liver injury and tumorigenesis

**DOI:** 10.1172/JCI194441

**Published:** 2025-12-16

**Authors:** Xiaowen Ma, Xiaoli Wei, Mengwei Niu, Chen Zhang, Zheyun Peng, Wanqing Liu, Junrong Yan, Xiaoyang Su, Lichun Ma, Shaolei Lu, Wei Cui, Hiromi Sesaki, Wei-Xing Zong, Hong-Min Ni, Wen-Xing Ding

**Affiliations:** 1 Department of Pharmacology, Toxicology and Therapeutics, University of Kansas Medical Center, Kansas City, Kansas, USA.; 2 Department of Pharmaceutical Sciences, Eugene Applebaum College of Pharmacy and Health Sciences and; 3 Department of Pharmacology, School of Medicine, Wayne State University, Detroit, Michigan, USA.; 4 Rutgers Cancer Institute, Rutgers University, Piscataway, New Jersey, USA.; 5 Cancer Data Science Laboratory and; 6 Liver Cancer Program, Center for Cancer Research, National Cancer Institute, Bethesda, Maryland, USA.; 7 Department of Pathology and Laboratory Medicine, Alpert Medical School of Brown University, Providence, Rhode Island, USA.; 8 Department of Pathology, The University of Kansas Medical Center, Kansas City, Kansas, USA.; 9 Department of Cell Biology, Johns Hopkins University School of Medicine, Baltimore, Maryland, USA.; 10 Department of Internal Medicine, The University of Kansas Medical Center, Kansas City, Kansas, USA.

**Keywords:** Cell biology, Hepatology, Liver cancer

## Abstract

Mitochondrial fission is mediated by dynamin-related protein 1 (gene name *DNM1L*) and fusion by mitofusins (MFN1 and MFN2) and optic atrophy 1. The role of mitochondrial dynamics in liver disease and cancer remains poorly understood. We analyzed single, double, and triple liver-specific KO mice lacking mitochondrial fission and fusion proteins using systematic analyses of mitochondrial morphology, untargeted metabolomics, RNA-seq, hydrodynamic tail vein injection of oncogenes, and human hepatocellular carcinoma samples. Liver-specific *Dnm1l-*KO (L*-Dnm1l*–KO) mice showed increased alanine aminotransferase levels and hepatic fibrosis, with spontaneous liver tumors developing by 12 to 18 months of age. L-*Mfn1*– and L-*Mfn2*–KO mice showed no significant liver damage or tumor development, although a small percentage of L-*Mfn1*, *Mfn2* double KO mice developed tumors. *Dnm1l*, *Mfn1*, and *Mfn2* triple KO (TKO) mice experienced significantly reduced liver injury and fibrosis, along with decreased spontaneous and oncogene-induced tumorigenesis. L-*Dnm1l*–KO mice showed increased activation of the cGAS/STING/interferon pathway and pyrimidine metabolism, which were significantly normalized in TKO mice. Deletion of hepatic *cGas* reduced both basal and oncogene-induced liver injury and tumor development in L-*Dnm1l*–KO mice. These findings indicate that mitochondrial dynamics are crucial for maintaining hepatic pyrimidine metabolism and regulating the cGAS/STING-mediated immune response to prevent liver tumorigenesis.

## Abstract 2

Alterations in mitochondrial dynamics and the accumulation of large mitochondria contribute to liver tumor development.

## Graphical abstract

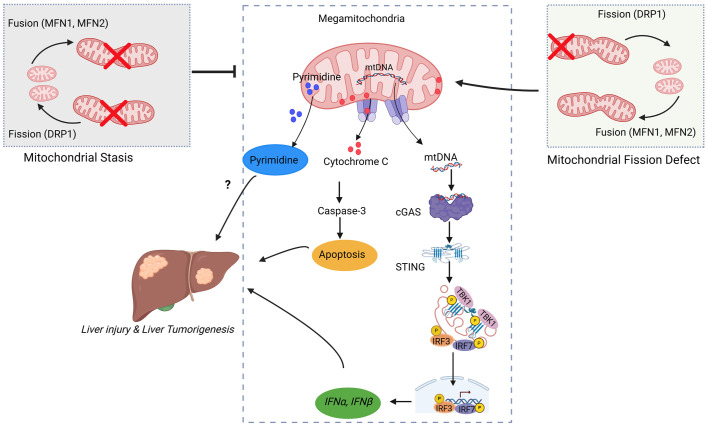

## Introduction

Primary liver cancer is the sixth most prevalent cancer and the third leading cause of global cancer mortality as of 2020, with an estimated 906,000 new cases and 830,000 deaths each year (1). Hepatocellular carcinoma (HCC) is the most common type of primary liver cancer that accounts for 75%–85% of instances and has had an increasing incidence over the years (1). Although early HCC is curable by surgical resection, in most cases, HCC is diagnosed at an advanced stage, from which the standard anti-angiogenic drugs, such as sorafenib and lenvatinib, can only slightly improve the survival rate (2). Recent studies have shown that mitochondria in HCC tumor tissues exhibit distinct morphological features, which are associated with HCC cell survival and promote the infiltration of tumor-associated macrophages (3, 4). Mitochondria size is strictly regulated by fission/fusion machinery: mitofusin 1 (MFN1), MFN2, and optic atrophy 1 mediate the fusion of mitochondrial outer and inner membranes, while dynamin-related protein 1 (DRP1) (gene name *DNM1L*) serves as a major fission protein, driving mitochondrial fragmentation (5, 6). Mitochondrial fusion is widely recognized for maintaining the fidelity of mtDNA, whereas dysfunctional mitochondria normally undergo fragmentation and tend to be removed by selective mitophagy and replaced via mitochondria biogenesis (5, 6). In addition to quality control, mitochondrial morphological homeostasis is also closely involved in various stress-induced cellular responses, including apoptosis, energy transmission, innate and sterile inflammation, cell cycle regulation, and dedifferentiation (6, 7). Although clinical evidence regarding mitochondrial size in HCC tumor tissues is controversial, increased levels of fission protein DRP1 and decreased levels of fusion protein MFNs have been observed in both human HCC and mouse models of liver cancer (8–11). Increased mitochondrial fission has been associated with a higher risk of distant metastasis and poor prognosis in patients with HCC (3, 8). However, it remains unclear whether the imbalanced fission and fusion events in cancer tissues represent an adaptive metabolic response for survival or actively contribute to HCC tumorigenesis.

Chronic liver injury is often associated with anomalous mitochondrial morphology (12–14), suggesting dysregulated mitochondrial dynamics in precancerous conditions. From an etiological perspective, metabolic dysfunction-associated steatotic liver disease (MASLD) and alcohol-associated liver disease (ALD) have emerged as major contributors to HCC development, and alcohol-associated cirrhosis is now the second most common cause of HCC worldwide, following HBV infection (15). Both MASLD and ALD have been shown to induce hepatic megamitochondria formation (13, 16). In ALD, megamitochondria formation is initially a respiratory adaptive response to facilitate alcohol metabolism, but megamitochondria accumulate under chronic alcohol exposure, leading to mitochondrial maladaptation and liver injury (16). Whether the long-standing megamitochondria also contribute to liver tumor formation is unknown. This study investigates the role of mitochondria fission/fusion homeostasis in liver tumorigenesis by generating genetically engineered mice with impaired mitochondrial fission (*Dnm1l*) and/or fusion (*Mfn1* or *Mfn2*) and performing systematic analysis of mitochondrial morphology, untargeted metabolomics, and RNA-seq.

## Results

### Abnormal and heterogeneous expression of DRP1/DNM1L in human HCC samples.

**Figure 1:**
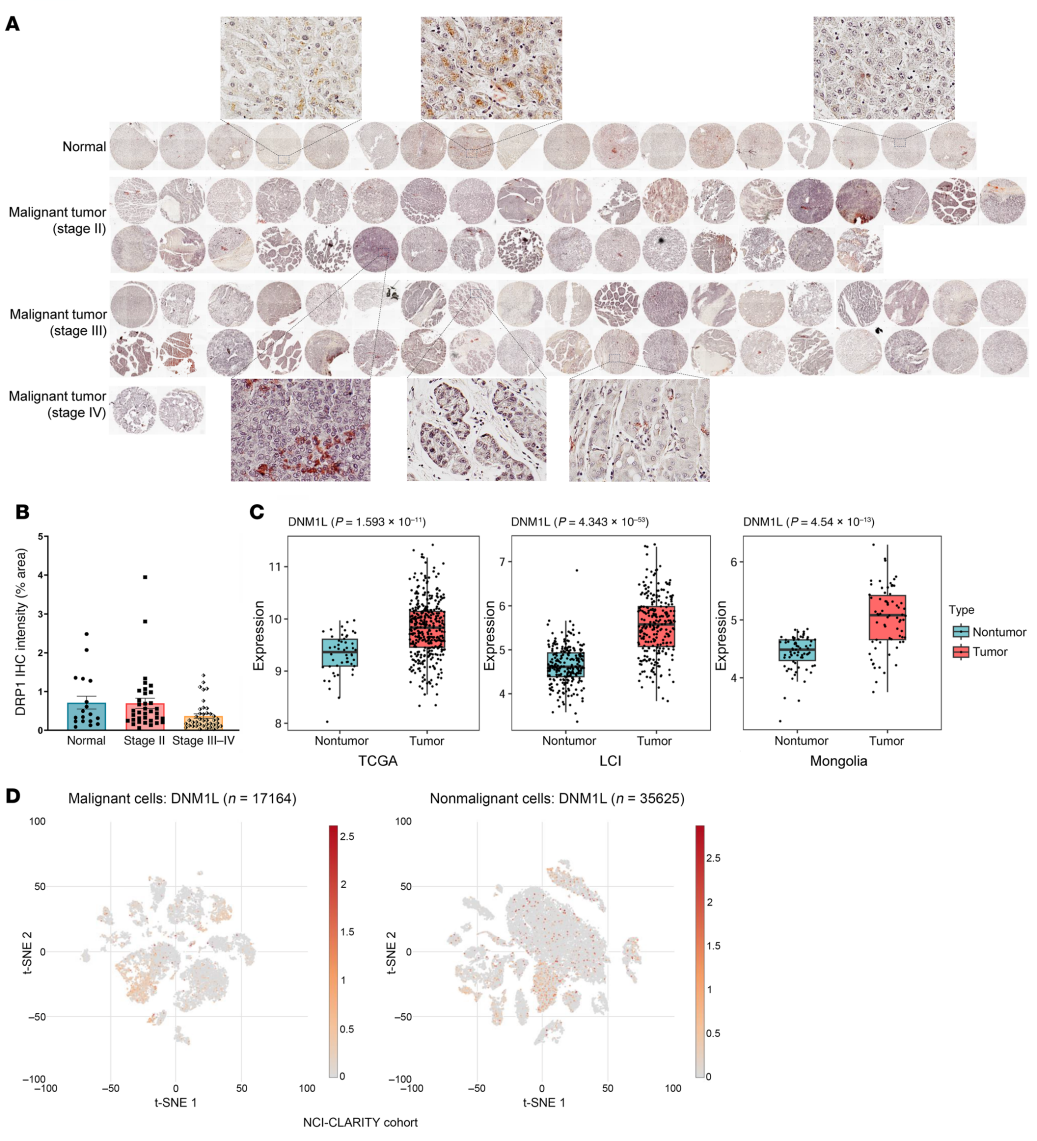
Heterogeneity of DRP1/*DNM1L* expression in human HCC samples. (**A**) IHC staining of DRP1 on human normal liver and HCC samples. The intensity of each staining was quantified using ImageJ (NIH). Original magnification, x20. (**B**) The value of each dot represents the optical density of 1 sample from **A**. Data are expressed as mean ± SE. *n* = 18, 35, 38, and 2 of normal, malignant tumor stage II, stage III, and stage IV. (**C**) Bioinformatics analysis of *DNM1L* expression in nontumor and HCC tumor tissues in the TCGA, LCI, and Mongolia databases. The box plots depict the minimum and maximum values (whiskers), the upper and lower quartiles, and the median. (**D**) scRNA-seq analysis of *DNM1L* expression in the NCI-CLARITY cohort dataset.

To determine whether changes in mitochondrial dynamic proteins and genes are associated with human HCC, we first examined the expression of DRP1 protein using clinical samples from tissue microarrays (TMAs). These TMAs contained 75 HCC samples, consisting of 35 stage II, 38 stage III, and 2 stage IV malignant tumors, along with 18 normal human liver samples (LV1002). IHC staining for DRP1 revealed significant heterogeneity in its expression between normal liver and HCC tissues, but no substantial differences were observed between them. However, there was a trend of decreased DRP1 expression in the more malignant HCC samples (Figure 1, A and B). Bioinformatics analysis utilizing The Cancer Genome Atlas (TCGA), the Liver Cancer Institute (LCI), and the Mongolia databases also indicated considerable heterogeneity in *DNM1L* expression in both normal human liver and HCC tissues. Interestingly, all 3 datasets showed a trend of increased *DNM1L* expression in HCC, though a small proportion of HCC samples exhibited relatively lower *DNM1L* expression levels (Figure 1C). Furthermore, single-cell analysis of the NCI-CLARITY cohort revealed significant variability in *DNM1L* expression within both malignant and nonmalignant cells. Only 17.6% of malignant cells and 9.9% of nonmalignant cells exhibited higher *DNM1L* expression (Figure 1D and Table 1). We conducted further analyses of gene expression related to mitochondria and HCC, focusing on mitochondrial dynamics, biogenesis regulators, tumor suppressors, the WNT/β-catenin pathway, mTOR-PI3K-AKT signaling, oxidative stress responses, growth factors, de novo pyrimidine synthesis, nucleotide interconversion, and nucleotide catabolism (Supplemental Figures 1 and 2; supplemental material available online with this article; https://doi.org/10.1172/JCI194441DS1). Our findings revealed a positive correlation between *DNM1L* expression and the genes *MNF1* and *MFN2*. This correlation likely indicates a compensatory mechanism that maintains mitochondrial dynamics. Additionally, *DNM1L* expression showed positive correlations with several tumor suppressor genes, including *TP53*, *ARID2*, and *CDKN2A*. Interestingly, we also found that *DNM1L* expression negatively correlated with genes involved in de novo pyrimidine synthesis. Overall, these data suggest that HCC cells exhibit abnormal and heterogeneous *DNM1L* expression, with a positive correlation between *DNM1L* and *MFN1* and *MFN2*.

### L-Dnm1l–KO mice have an increased number of megamitochondria, liver injury, fibrosis, and spontaneous liver tumorigenesis, which can be rescued by restoration of mitochondrial stasis.

**Figure 2:**
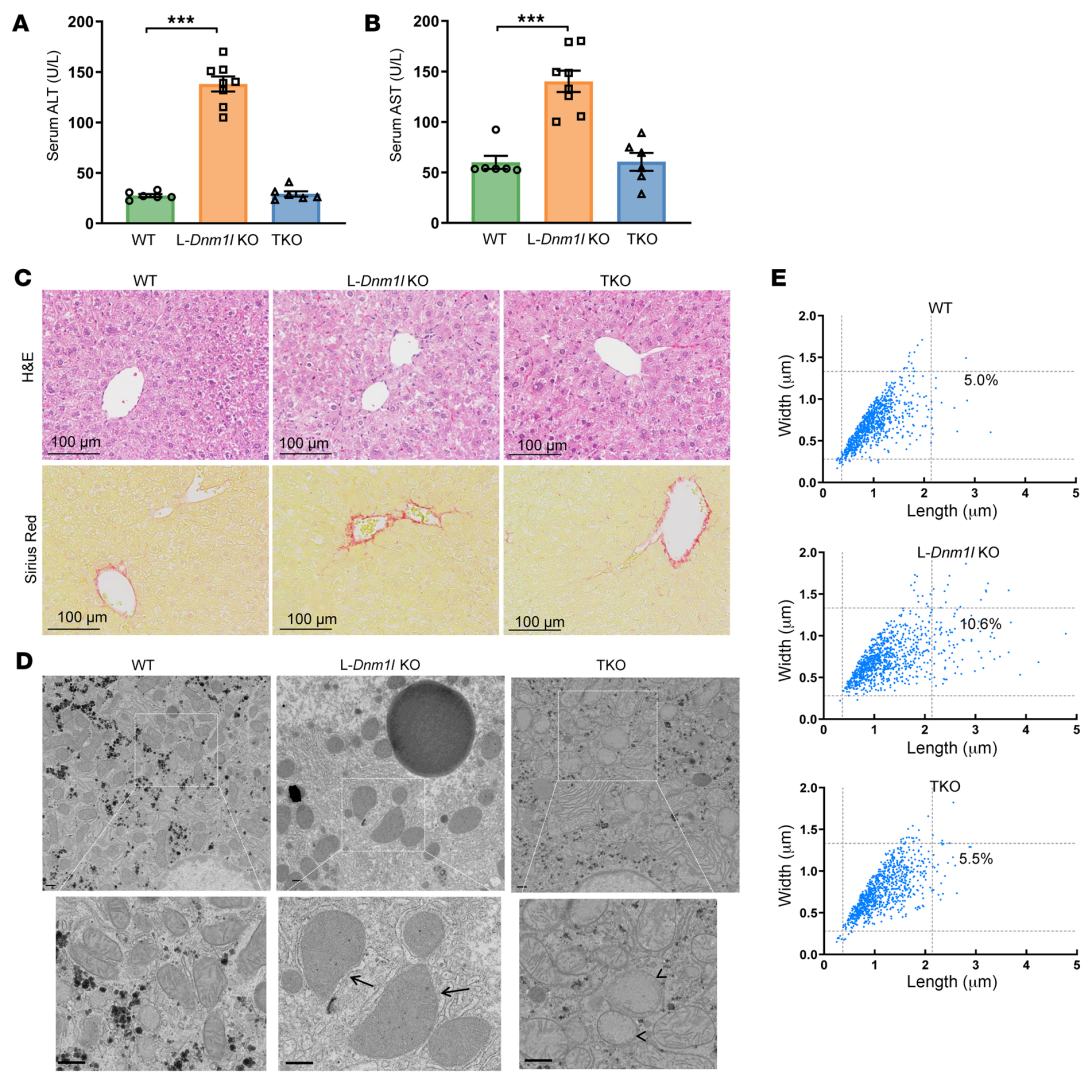
L-*Dnm1l–*KO mice have an increased number of megamitochondria and liver injury, restored by additional *Mfn1* and *Mfn2* ablation. (**A** and **B**) Serum ALT and AST activities of 2M mice with indicated genotypes. (**C**) Representative H&E and Sirius red staining from 2M mouse liver. Scale bars: 100 μm. (**D**) Representative EM images of the livers from 2M mice. Arrows denote megamitochondria, and arrowheads denote abnormal mitochondria cristae. Scale bars: 500 nm. (**E**) Quantification of mitochondrial size from EM images. The reference lines represent the 95th percentile in the WT group. Images from 3 mice were quantified in each group (mitochondria number = 600–900). All results are expressed as mean ± SE (*n* = 6–8). ***P < 0.001; 1-way ANOVA analysis with Dunnett’s test.

L-*Dnm1l*–KO; L-*Mfn1–* and L-*Mfn2*–double KO (DKO); and L-*Mfn1–,* L-*Mfn2–,* and *Dnm1l*–triple KO (TKO) mice were born at the expected Mendelian ratio, and the appearance of newborns was normal. An approximately 5-fold increase in serum alanine aminotransferase (ALT) and a 2-fold increase in serum aspartate aminotransferase (AST) activities were found in L-*Dnm1l*–KO mice at 2 months old (2M) compared with their matched WT littermates, which were completely rescued by further deletion of mitochondrial fusion proteins MFN1 and MFN2 (Figure 2, A and B). L-*Dnm1l*–KO mice had increased hepatic Sirius red staining and hepatic α-smooth muscle actin (α-SMA) levels than WT mice, both of which were blunted in TKO mice (Figure 2C and Supplemental Figure 3). Ultrastructure electron microscopy (EM) analysis showed heterogeneity of mitochondrial size in hepatocytes of mouse liver, but the number of large or elongated mitochondria was more than 2-fold higher in L-*Dnm1l*–KO mice than in WT or TKO mice (Figure 2, D and E). There was an increased number of mitochondria exhibiting loss or abnormal reorganization of mitochondrial cristae in TKO hepatocytes (Figure 2D, arrowheads). Mitochondrial energetic analysis using Seahorse also revealed a decreased oxygen consumption rate in TKO hepatocytes compared with WT hepatocytes (Supplemental Figure 4). Furthermore, mitophagy decreased in L-*Dnm1l*–KO mouse livers, and the reduced mitophagy was restored in TKO mouse livers (Supplemental Figure 5, A and B). Overexpression of MFN2 increased the number of elongated mitochondria and resulted in liver injury (Supplemental Figure 6, A–D). This is similar to the effects observed in L-*Dnm1l*–KO mice, supporting the idea that the accumulation of megamitochondria in hepatocytes can lead to liver injury. Furthermore, levels of serum ALT, hepatic triglyceride, and cholesterol as well as positive Sirius red staining and expression of inflammatory cytokines were markedly decreased in CDAHFD-fed DKO mice compared with WT mice (Supplemental Figure 7, A–G). These data indicate that loss of hepatic *Mfn1* and *Mfn2* protects against diet-induced metabolic dysfunction–associated steatohepatitis (MASH).

**Figure 3:**
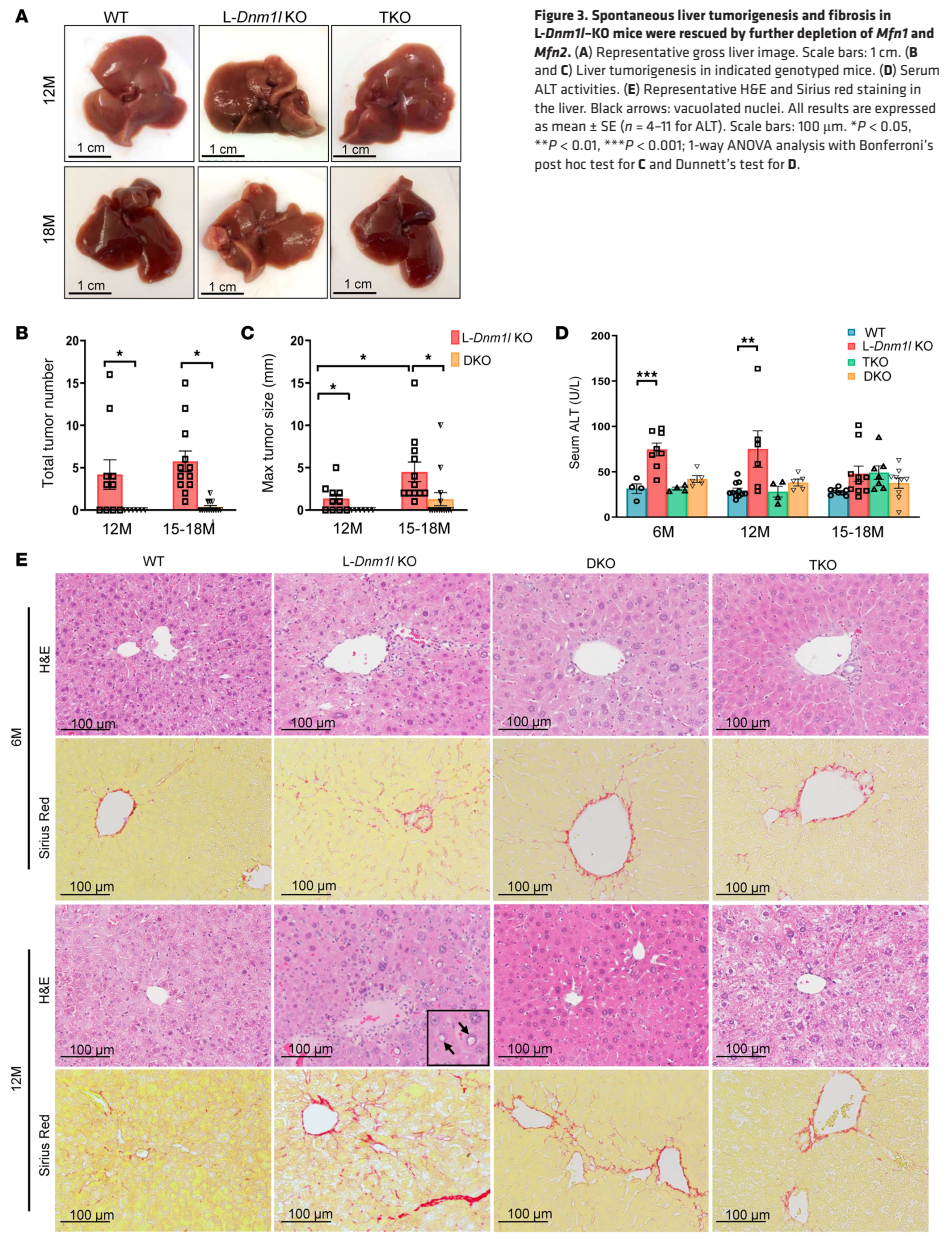
Spontaneous liver tumorigenesis and fibrosis in L-*Dnm1l*–KO mice were rescued by further depletion of *Mfn1* and *Mfn2*. (**A**) Representative gross liver image. Scale bars: 1 cm. (**B** and **C**) Liver tumorigenesis in indicated genotyped mice. (**D**) Serum ALT activities. (**E**) Representative H&E and Sirius red staining in the liver. Black arrows: vacuolated nuclei. All results are expressed as mean ± SE (*n* = 4–11 for ALT). Scale bars: 100 μm. *P < 0.05, **P < 0.01, ***P < 0.001; 1-way ANOVA analysis with Bonferroni’s post hoc test for **C** and Dunnett’s test for **D**.

Starting at the age of 12M, L-*Dnm1l*–KO mice developed spontaneous liver tumors, with 64% incidence at 12M and 92% incidence after 15–18M in male mice. The incidence of liver tumors was much lower in female L-*Dnm1l–*KO mice than in the age-matched L-*Dnm1l*–KO male mice (Table 2). Neither L-*Mfn1–*KO nor L-*Mfn2–*KO mice had an obvious liver injury or visible tumor formation (Figure 3, A–D). However, a small proportion (29%) of DKO mice developed liver tumors at 15M, but the tumor burden and maximum size were significantly lower than those of age-matched L-*Dnm1l*–KO mice (Figure 3, B and C). No visible tumors were found in the TKO mouse livers (Figure 3, A and B, and Table 2), indicating that reestablishment of mitochondrial stasis is critical to rescue the liver tumorigenesis induced by the unbalanced mitochondrial fission and fusion. The elevated serum ALT activity in L-*Dnm1l*–KO mice persisted until 12M, with the peak at 2M, and normalized at 15–18M (Figure 2A and Figure 3D). No significant changes in ALT activities were found in TKO or DKO mice (Figure 3D). Starting at 6M, the nuclei in L-*Dnm1l*–KO hepatocytes showed a pleomorphic pattern with an increased number of vacuolated nuclei (Figure 3E, arrows), which were more obvious in 12M L-*Dnm1l*–KO hepatocytes but were absent in age-matched WT and TKO hepatocytes (Figure 3E). Moreover, we found severe liver fibrosis in L-*Dnm1l–*KO mice, but not TKO or DKO, as early as 6M and persistent to 12M, showing prototypical perisinusoidal (chicken wire) collagen fiber deposition by Sirius red staining and increased hepatic α-SMA (Figure 3E and Supplemental Figure 3, B and C), indicating increased wound-healing response to the chronic liver injury in L-*Dnm1l*–KO mice. Taken together, impaired mitochondrial fission in hepatocytes leads to increased liver injury, fibrosis, and liver tumorigenesis in mice, which can be rescued with hepatic mitochondria stasis.

### Loss of liver Dnm1l activates the cGAS/STING/interferon pathway and induces liver inflammation, which are attenuated in TKO mouse livers.

**Figure 4:**
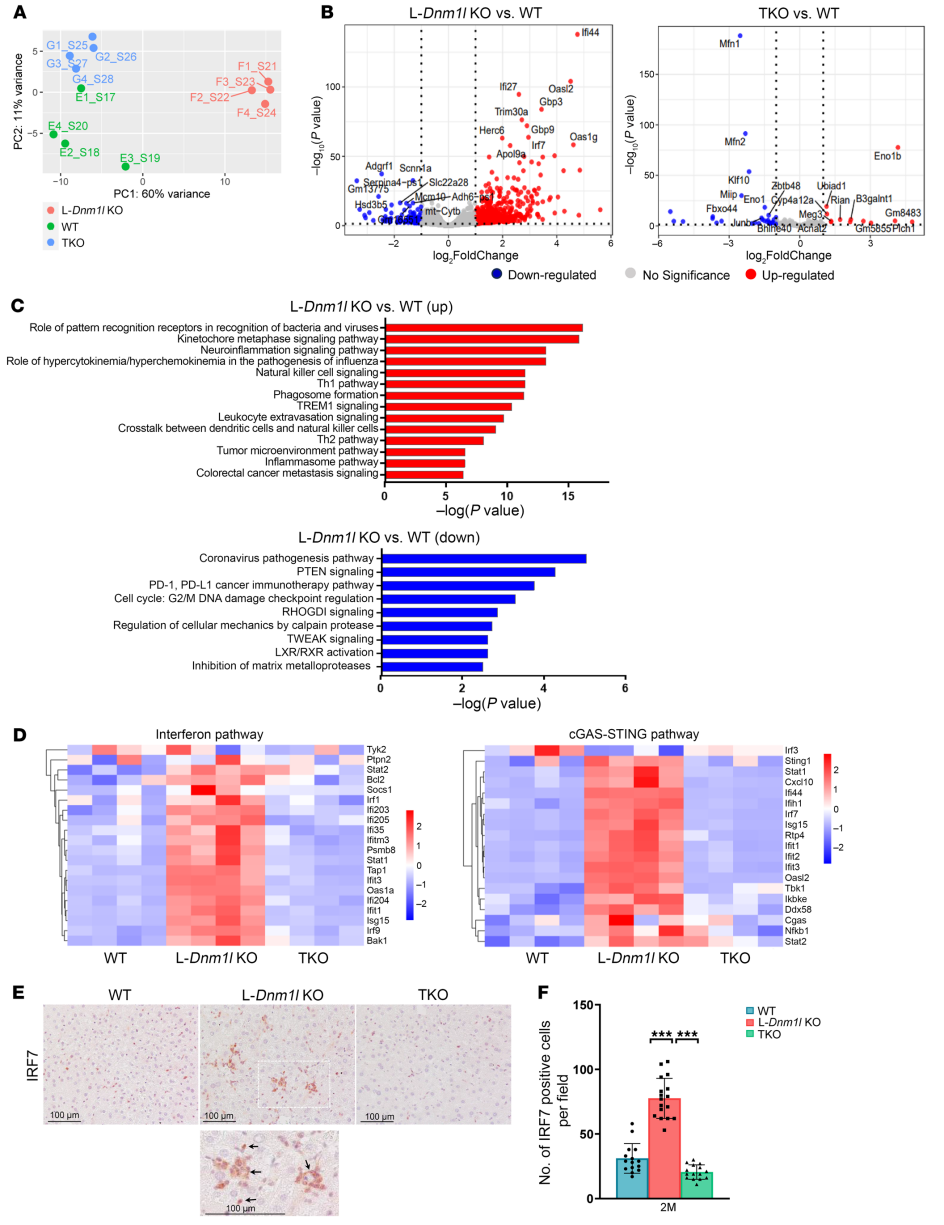
Loss of liver *Dnm1l* activates the cGAS/STING/interferon pathway and promotes tumor microenvironment transformation, blunted in TKO mice. (**A**) PCA of RNA-seq dataset. (**B**) Volcano plot of RNA-seq dataset. (**C**) The top up- and downregulated pathways identified by Ingenuity Pathway Analysis from RNA-seq dataset of indicated mouse liver tissues. (**D**) Heatmap of genes involved in the interferon and cGAS/STING pathways from the RNA-seq dataset. (**E** and **F**) Representative IHC staining of IRF7 in 2M mouse liver. At least 5 fields were quantified from each mouse (*n* = 3 mice). Scale bars: 100 μm. All results are expressed as mean ± SD. ***P < 0.001; 1-way ANOVA analysis with Bonferroni’s post hoc test.

To investigate the potential mechanisms underlying early-stage liver injury in L-*Dnm1l*–KO mice, we performed RNA-seq analysis on liver tissues from 2M and 6M mice. In 2M mice, principal component analysis (PCA) revealed a distinct separation in gene expression between the WT and L-*Dnm1l*–KO groups, while the TKO group exhibited a gene expression profile closer to that of WT mice (Figure 4A). A similar gene distribution pattern was identified in 6M mice (Supplemental Figure 8A). In 2M mice, 743 upregulated and 125 downregulated genes were identified in L-*Dnm1l*–KO mice compared with the WT, whereas only 48 differential genes were detected between the TKO and WT mice (Figure 4B). These findings suggest that the gene expression alterations in L-*Dnm1l–*KO mice were effectively corrected by the reconstitution of mitochondria stasis through a simultaneous block of fusion and fission events in TKO mice. Compared with WT mice, many of the top upregulated genes in L-*Dnm1l*–KO mice, such as *Ifi44*, *Ifi27*, *Oasl2,* and *Irf7*, belong to the cyclic GMP-AMP Synthase (cGAS)/Stimulator of Interferon Genes (STING)/interferon innate immune pathway. In contrast, *Mfn1* and *Mfn2* are among the most significantly downregulated genes in TKO mouse livers (Figure 4B), confirming a successful deletion of these genes in TKO mice. Ingenuity Pathway Analysis revealed a strong activation of the inflammation-related and tumor microenvironment pathways in L-*Dnm1l*–KO mice (Figure 4C), along with a notable upregulation of genes within the cGAS/STING/interferon pathway, as shown in the heatmap (Figure 4D). Interestingly, the increased expression of cGAS/STING/interferon pathway genes in L-*Dnm1l–*KO mice persisted from 2M to 6M but was nearly completely abolished in TKO mice (Figure 4D and Supplemental Figure 8B). In addition, no significant changes in the expression of cGAS/STING/interferon pathway genes were observed in DKO mice compared with WT mice (Supplemental Figure 8B), suggesting that inhibiting mitochondria fission, rather than fusion, is more critical for the activation of the cGAS/STING innate immune pathway.

In parallel, IHC staining revealed enriched IRF7-positive immune cell infiltration in the livers of 2M L-*Dnm1l*–KO mice (Figure 4, E and F). Protein levels of cGAS, STING, p-TBK1, IRF3, and IRF7 were significantly elevated in the normal tissues of 15–18M L-*Dnm1l–*KO mice, with even higher levels detected in the tumor tissues. However, most of these increases were reduced in age-matched TKO mice (Supplemental Figure 8C). Western blot analysis confirmed the successful deletion of hepatic DRP1, MFN1, and MFN2 in the KO, TKO, and DKO mice (Supplemental Figure 8D). In L-*Dnm1l–*KO mouse liver, we found an increased number of F4/80^+^ macrophages and myeloperoxidase^+^ neutrophils compared with WT and TKO mice, and the F4/80^+^ macrophage-enriched areas also had increased IRF7^+^ cells (Supplemental Figure 9, A and B, and Supplemental Figure 10). This phenomenon was particularly obvious in 2M mice but was attenuated by 6M. Consistent with the IHC staining, RNA-seq analysis indicated decreased expression of gene signatures in hepatocytes and increased expression in hepatic stellate cells and Kupffer cells/macrophages in 2M and 6M L-*Dnm1l–*KO mice, although the changes were less prominent in 6M. Interestingly, regardless of age, the alterations in cell-type–specific gene signatures were significantly blunted in TKO mice (Supplemental Figure 11, A and B). Furthermore, the levels of cleaved caspase-3 and ER stress markers, including BIP and CHOP, were markedly increased in the livers of 2M L-*Dnm1l*–KO mice (Supplemental Figure 9C), suggesting that hepatic DRP1 loss may induce ER stress, apoptosis, and inflammation, which may contribute to the early stage of liver injury in mice.

### Enhanced pyrimidine synthesis and decreased mitochondrial gene expression in L-Dnm1l–KO mouse livers are restored in TKO mice.

**Figure 5:**
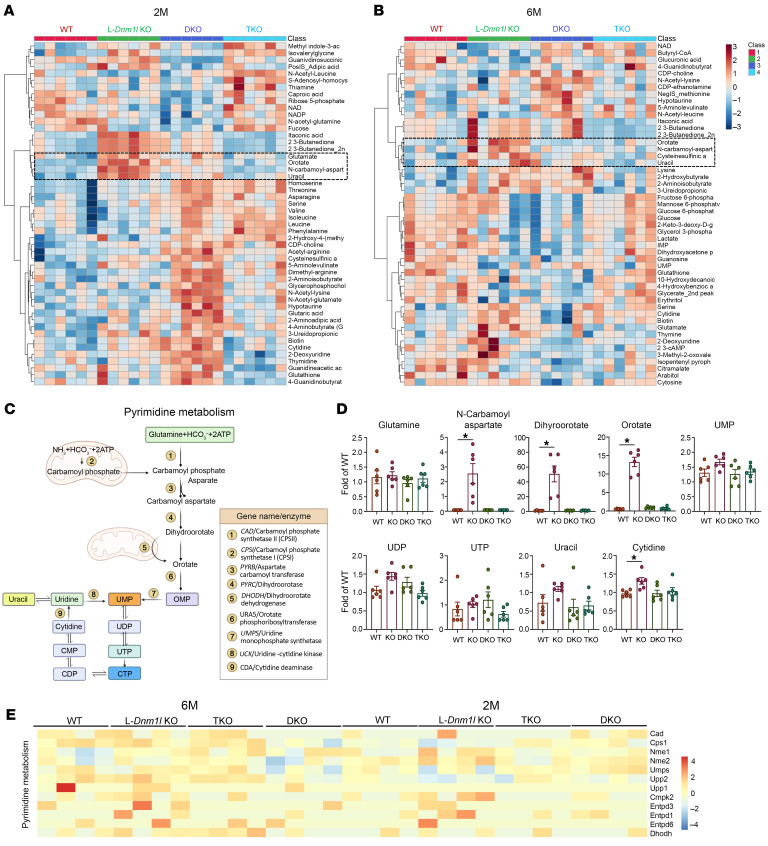
Increased pyrimidine synthesis and metabolism in the liver of L-*Dnm1l*–KO but not TKO mice. Polar metabolites were extracted and subjected to LC-MS. Heatmap of targeted metabolomics analysis of indicated 2M (**A**) and 6M (**B**) mouse liver tissues (*n* = 6). Pyrimidine-related metabolites are in boxes. (**C**) A summary graph of mitochondria and pyrimidine synthesis pathway. (**D**) Levels of key pyrimidine metabolites from untargeted metabolomics analysis (*n* = 6). (**E**) Heatmap of pyrimidine metabolism genes from the RNA-seq analysis (*n* = 6). All results are expressed as mean ± SE. *P < 0.05; 1-way ANOVA analysis with Bonferroni’s post hoc test.

Metabolomics analysis of the mitochondrial TCA cycle, amino acids, and nucleotides showed that pyrimidine-related metabolites, including glutamate, orotate, N-carbamoyl-aspartate, and uracil, exhibited the most significant increases in the livers of 2M L-*Dnm1l*–KO mice compared with WT, DKO, and TKO mice (Figure 5A). This trend was similarly observed in the livers of 6M mice (Figure 5B). To rigorously validate the changes in pyrimidine metabolism, we conducted untargeted metabolomics analysis using a different batch of 2M mouse liver tissues. The results confirmed an increase in hepatic pyrimidine metabolites, specifically dihydroorotate and orotate, which were elevated approximately 50-fold and 14-fold respectively, in L-*Dnm1l*–KO mice compared with WT mice (Figure 5, C and D). Therefore, these results provide direct evidence that mitochondrial morphology affects pyrimidine metabolism. In contrast, no significant differences were observed in DKO or TKO mice relative to WT mice (Figure 5, C and D). Consistent with the metabolomics data, RNA-seq analysis revealed increased expression of genes related to pyrimidine metabolism in 2M L-*Dnm1l–*KO mice compared with WT mice. These changes were less pronounced in 6M mice as well as in TKO and DKO mice (Figure 5E). Hepatic protein levels of dihydroorotate dehydrogenase (DHODH), which is a rate-limiting mitochondrial enzyme in the de novo pyrimidine synthesis pathway, showed a significant increase in the livers of L-*Dnm1l*–KO mice and were attenuated in the TKO mouse livers (Supplemental Figure 12).

To better understand the broader mitochondrial dysfunction in L-*Dnm1l–*KO mice, we next examined gene expression profiles associated with various mitochondrial components and functions. This analysis included genes related to complexes I, II, III, and IV, as well as mitochondrial cristae architecture, metabolic transporters, mitochondrial transcription, protein import, RNA/tRNA modification and processing, proteostasis and mitophagy, tRNA amino acid synthetases, ubiquinone synthesis, mtRibosome subunits and assembly factors, mitochondrial translation, and mtDNA replication. In 2M L-*Dnm1l*–KO mice, gene expression in these pathways generally showed a downward trend compared with WT mice but was largely corrected in TKO mice. However, the overall gene expression in the above pathways was higher in 6M WT mice compared with 2M WT mice, and the differences between L-*Dnm1l*–KO mice and WT mice were substantially reduced (Supplemental Figures 13–17), suggesting possible adaptive responses to mitochondrial dysfunction developed with age in L-*Dnm1l*–KO mice.

### L-Dnm1l–KO mice develop hepatocellular adenoma associated with increased tumor-related signaling pathways.

**Figure 6:**
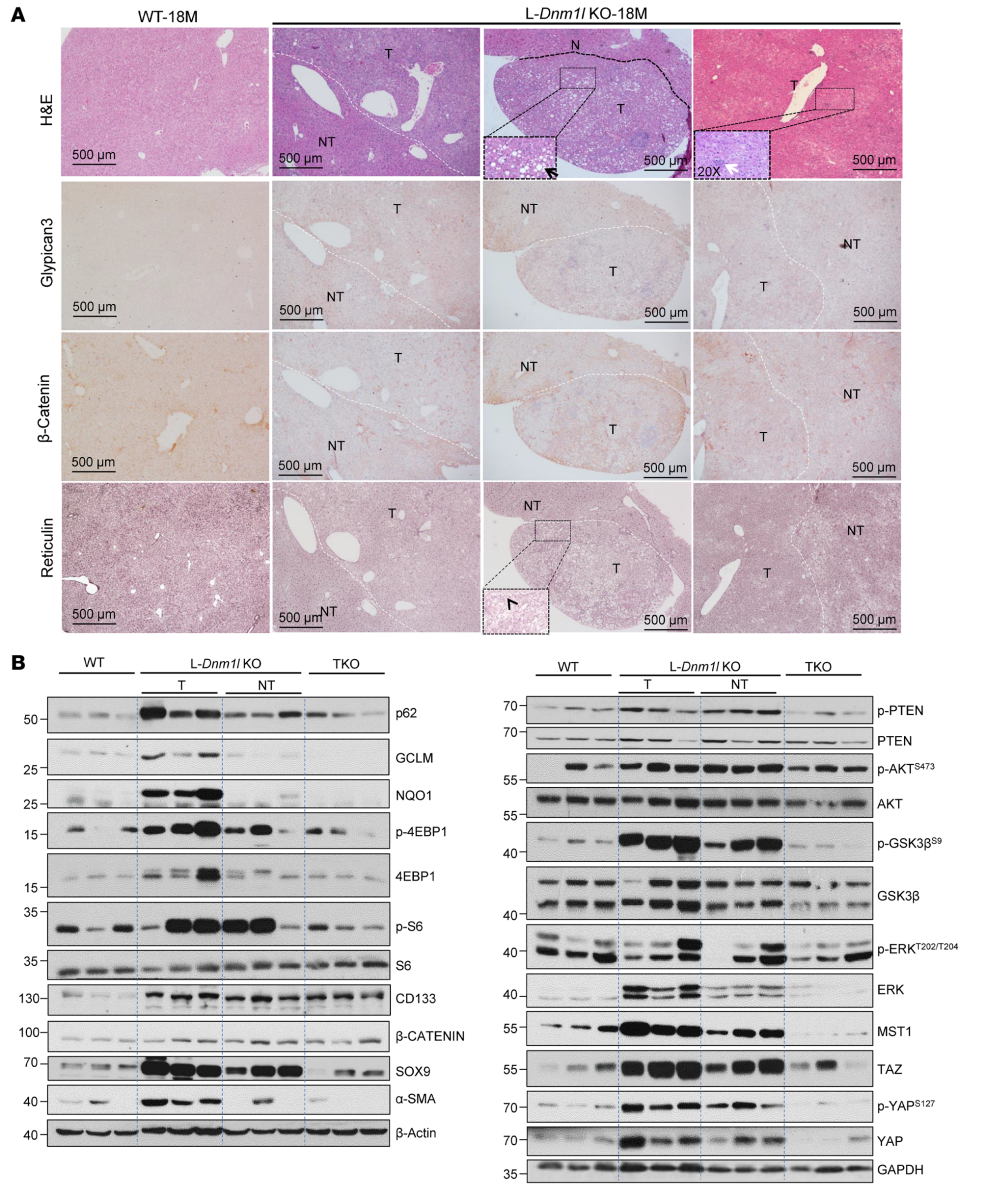
Hepatocellular adenoma in L-*Dnm1l*–KO mice. (**A**) Representative H&E and IHC staining of Glypican 3 and β-CATENIN as well as reticulin staining in 18M L-*Dnm1l*–KO and matched WT mouse liver. The white dotted line marks the boundary of tumor and nontumor areas. The black arrow indicates hepatocellular adenoma with lipid accumulation, the white arrow indicates infiltrated inflammatory cells, and the arrowhead indicates the reticulin^+^ staining. Scale bars: 500 μm. Zoom, ×20. (**B**) Western blot analysis from total liver lysates or tumor (T) and nontumor (NT) tissues of indicated 18M mice.

To characterize the tumor type in L-*Dnm1l–*KO mice, we performed histopathological analysis. H&E staining of tumor sections revealed the presence of hepatocellular adenoma in L-*Dnm1l–*KO mice. The adenomas lacked portal triads and were poorly defined, with benign hepatocytes arranged in plates of regular thickness accompanied by infiltrated inflammatory cells (Figure 6A, white arrow). Some tumor cells also showed marked steatosis, forming a steatotic adenoma and indicating a deficiency of lipid metabolism (Figure 6A, black arrow). Reticulin was utilized to highlight the normal thickness of liver cell plates, excluding the diagnosis of well-differentiated HCC. Our findings indicate that certain liver tumors exhibited steatosis and intact reticulin fibers and lacked Glypican 3 and nuclear β-catenin staining, which are characteristics of adenoma (Figure 6A, arrowhead).

Next, we assessed tumor heterogeneity in spontaneous tumors from L-*Dnm1l–*KO mice using Western blot analysis and identified 3 distinct types of changes. Increased levels of hepatic p62 have oncogenic effects by promoting the activation of NRF2 and mTORC1 pathways in the liver (17, 18). First, there was a consistent increase in tumor-related signaling pathways in all 3 tumors, including NRF2-related (p62, GCLM, and NQO1), PTEN/AKT–related (p-PTEN, p-AKT, and p-GASK3β), SOX9, SMA, mammalian STE20-like kinase 1 (MST1)/yes-associated protein (YAP)/TAZ pathways. Second, there was an increase in mTOR (p-S6, p-4EBP1) and p-ERK, though with significant variation among the tumors. Third, there were increases in both tumor and tumor-adjacent normal tissues in L-*Dnm1l–*KO mice, including p-S6, CD133, SOX9, p-PTEN, p-AKT, p-GSK3β, p-ERK, and MST1-YAP-TAZ (Figure 6B).

We next determined whether adenoma cells also lost the Dnm1l gene. Results from Western blot analysis using total liver lysates confirmed the deletion of DRP1 in the livers of L-*Dnm1l*–KO and TKO mice. Among the 3 spontaneous tumors in the L-*Dnm1l*–KO mice, 1 showed no detectable DRP1, another had relatively lower DRP1 levels, and 1 exhibited remarkably higher DRP1 expression (Supplemental Figure 18A). IHC staining results showed that DRP1 levels were significantly lower in both normal and spontaneous tumor tissues of L-*Dnm1l*–KO mouse livers compared with WT mice. WT normal liver tissues showed cytosolic DRP1 staining in distinct zonation, with higher DRP1 expression in the central vein area and lower expression in the portal vein area (Supplemental Figure 18B). The distinct zonational expression of DRP1 is consistent with previous findings that periportal and pericentral mitochondria are morphologically and functionally distinct in mouse liver (19). However, DRP1 staining was substantially reduced in L-*Dnm1l*–KO normal and tumor tissues. Interestingly, strong DRP1 staining persisted in a subset of immune cells and the endothelial cells of blood vessels (Supplemental Figure 18B, arrows). These findings suggest that the adenoma tumor cells in L-*Dnm1l*–KO mice are most likely derived from DRP1-KO hepatocytes. However, because we could only examine a limited number of spontaneous tumor tissues due to their small size, we cannot exclude the possibility that some tumors may originate from residual WT hepatocytes that carry mutations in L-*Dnm1l*–KO mice. Overall, these results indicate that hepatocellular adenoma, but not HCC, develops in L-*Dnm1l*–KO mice.

### Increased DNA damage, senescence, and compensatory proliferation in the liver of L-Dnm1l–KO but not TKO mice.

**Figure 7:**
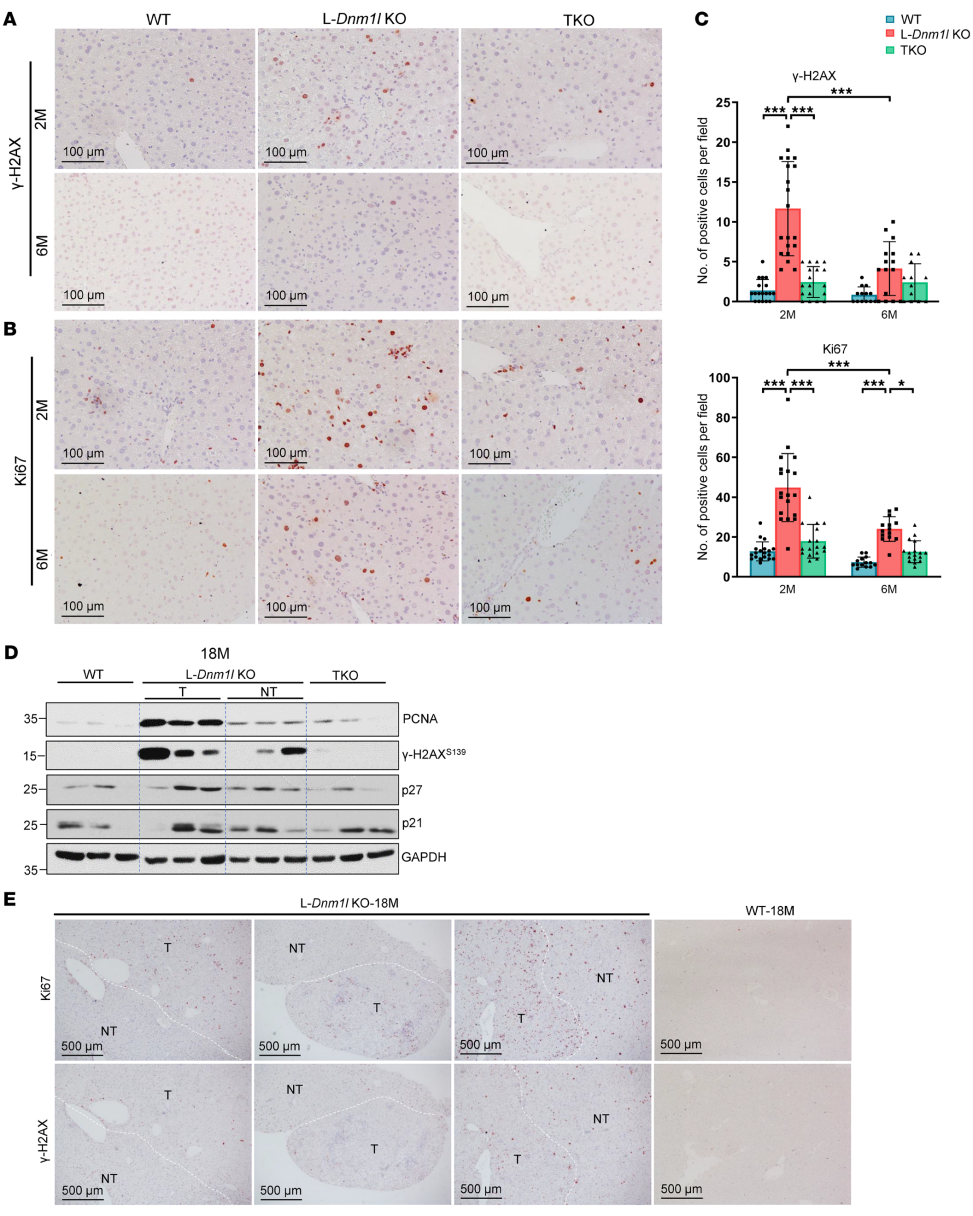
Increased DNA damage and proliferation in L-*Dnm1l*–KO but not TKO mice. (**A** and **B**) Representative IHC staining of γ-H2AX and Ki67 of indicated mouse genotypes. Scale bars: 100 μm. (**C**) At least 5 fields were quantified from each mouse (*n* = 3 mice). All results are expressed as mean ± SD. *P < 0.05, ***P < 0.001; 1-way ANOVA analysis with Bonferroni’s post hoc test. (**D**) Western blot analysis from total liver lysates or tumor and nontumor tissues of indicated 18M mice. (**E**) Representative IHC staining of γ-H2AX and Ki67 in 18M indicated genotyped mice. The white dotted line marks the boundary of tumor (T) and nontumor (NT) areas. Scale bars: 500 μm.

DNA damage and cell proliferation play a crucial role in cancer development and progression. Results from the IHC staining showed an increased number of phosphorylated γ-H2AX and Ki67^+^ cells in 2M L-*Dnm1l–*KO mouse liver (Figure 7, A–C). Intriguingly, the number of phosphorylated γ-H2AX and Ki67^+^ hepatocytes was significantly decreased in 6M L-*Dnm1l*–KO mice compared with 2M, suggesting a possible ongoing DNA lesion repair and adaptive process in these mice (Figure 7, A–C). Consistent with this, p21 and p27, the target genes of tumor suppressor p53 — whose induction leads to transcriptional downregulation of many cell cycle genes and levels of PCNA and γ-H2AX — were upregulated in the tumors of 18M L-*Dnm1l*–KO mice compared with WT mice (Figure 7D). There were higher numbers of γ-H2AX and Ki67^+^ cells in the tumor areas than the adjacent normal hepatocytes (Figure 7E). Furthermore, the number of β-galactosidase^+^ (β-Gal^+^) senescent cells increased in 18M L-*Dnm1l–*KO and TKO mouse livers, although it only increased in the 2M TKO but not L-*Dnm1l*–KO mouse livers (Supplemental Figure 19, A and B). Collectively, these data suggest that L-*Dnm1l*–KO mice have increased DNA damage, and compensatory proliferation in the liver as early as 2M, which is absent in TKO mice.

### Genetic restoration of mitochondrial stasis abolishes oncogene-driven liver cancer.

**Figure 8:**
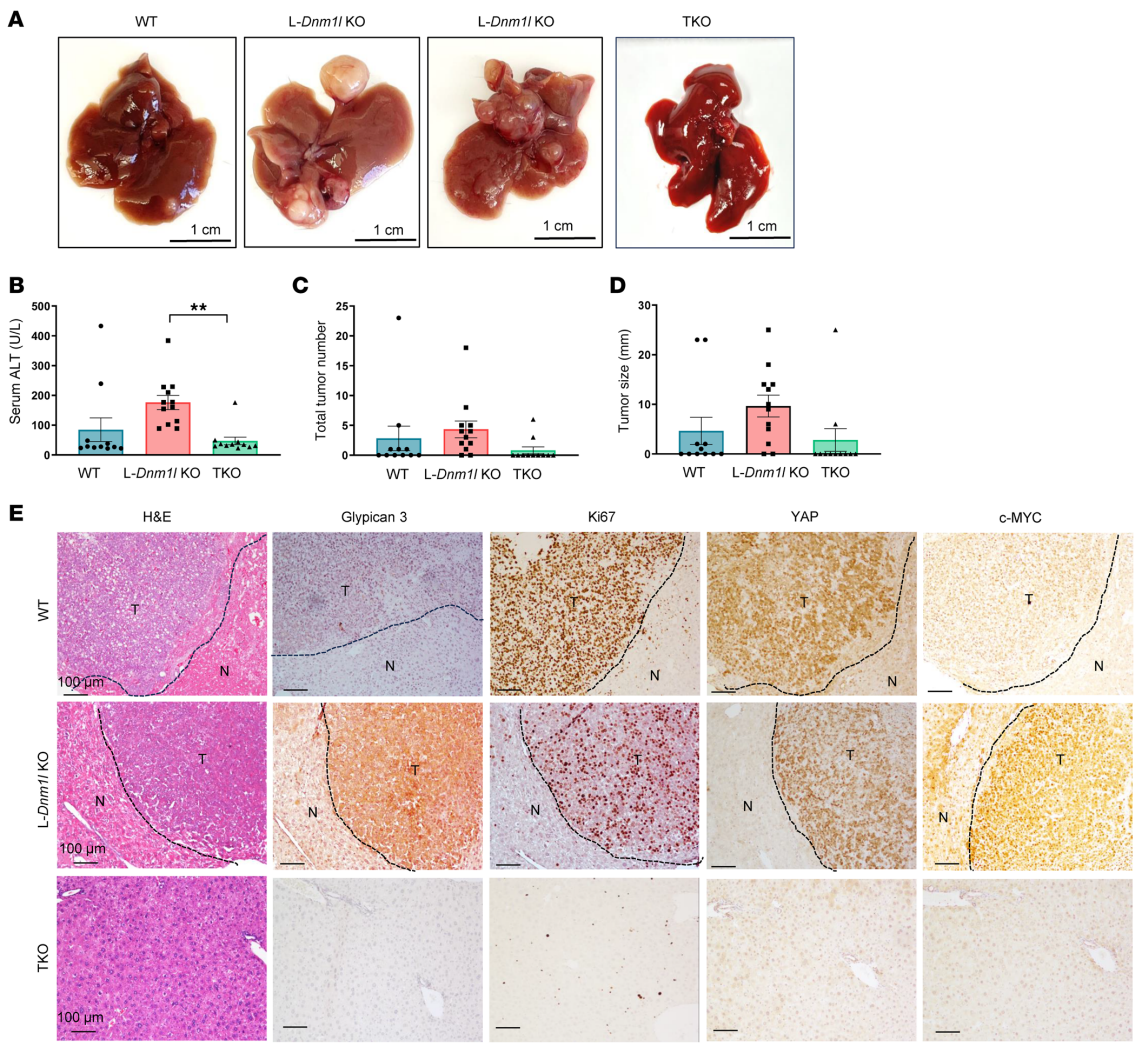
Increased oncogene-driven liver tumorigenesis in L-*Dnm1l*–KO but not TKO mice. Sleeping beauty transposon (SB10) and c-MYC/YAP^S127A^ were delivered into 8-week-old male mice of indicated genotypes through hydrodynamic tail vein injection. Liver tissues and blood were collected 8 weeks after injection. (**A**) Representative gross mouse liver images of indicated genotypes. Scale bars: 1 cm. (**B**) Serum ALT activities, (**C**) the number of tumors per mouse liver, and (**D**) tumor size are quantified. All results are expressed as mean ± SE (*n* = 11–12). **P < 0.01; 1-way ANOVA analysis with Dunnett’s test. (**E**) Representative H&E and IHC staining of indicated proteins in livers from mice with specific genotypes. The black dotted line marks the boundary of tumor (T) and nontumor (N) areas. Scale bars: 100 μm.

To determine the role of mitochondria dynamics in oncogene-induced liver cancer, we used the sleeping beauty transposon and hydrodynamic tail vein injection system to deliver *c*-*MYC* and the constitutively active *YAP* mutant (*YAP S127A*), which has been characterized to induce HCC (20). After 8 weeks following the hydrodynamic injection of *c-MYC/YAP*, visible tumor nodules appeared in mouse liver, with both tumor size and numbers, as well as serum ALT levels, increased in L-*Dnm1l*–KO mice. Strikingly, hydrodynamic injection of *c-MYC/YAP* did not increase serum ALT levels in TKO mice, and only 2 out of 11 TKO mice developed visible tumors (Figure 8, A–D). H&E staining revealed typical HCC features of these tumors with increased nuclear cytoplasmic ratios. IHC staining showed increased Glypican 3 staining and Ki67^+^ cells.

IHC staining showed cytosolic DRP1 staining in WT mouse tumors but significantly reduced DRP1 staining in normal and tumor tissues in L-*Dnm1l*–KO mice, with only strong staining in a subset of immune cells (Supplemental Figure 20, arrows). Western blot analysis of liver tissues from normal and tumor samples in WT, L-*Dnm1l*–KO, and TKO mice showed that DRP1 levels are notably lower in L-*Dnm1l–*KO and TKO liver tissues (both tumor and adjacent normal tissues) compared with WT tissues. Levels of DRP1, MFN1, and MFN2 were generally lower in tumor samples compared with adjacent normal tissues. There was a slight increase in the levels of outer mitochondrial membrane proteins and the inner membrane protein TIM23 in L-*Dnm1l*–KO tumors, but no change was observed in the mitochondrial matrix proteins (Supplemental Figure 21, A and B). Notably, the size of mitochondria in tumor cells of WT mice was larger than in adjacent normal cells. In contrast, the normal cells neighboring the tumors in L-*Dnm1l*–KO mice already exhibited larger mitochondria, with sizes being comparable between normal and tumor cells (Supplemental Figure 22).

These tumors were also positive for c-MYC and YAP proteins, confirming that they were driven by c-MYC/YAP (Figure 8E and Supplemental Figure 23). Notably, the levels of c-GAS, STING, TBK1, and IRF7, but not IRF3, were elevated in tumors of WT mice and further increased in L-*Dnm1l–*KO mice. There were no significant changes in c-GAS, STING, TBK1, and IRF7 in nontumor sections of TKO mice compared with WT mice (Supplemental Figure 23). These data suggest that loss of hepatic *Dnm1l* promotes oncogene-induced HCC, which is blunted by restoration of hepatic mitochondrial stasis in mice.

### Loss of hepatic cGAS inhibits spontaneous and oncogene-driven liver tumor development.

**Figure 9:**
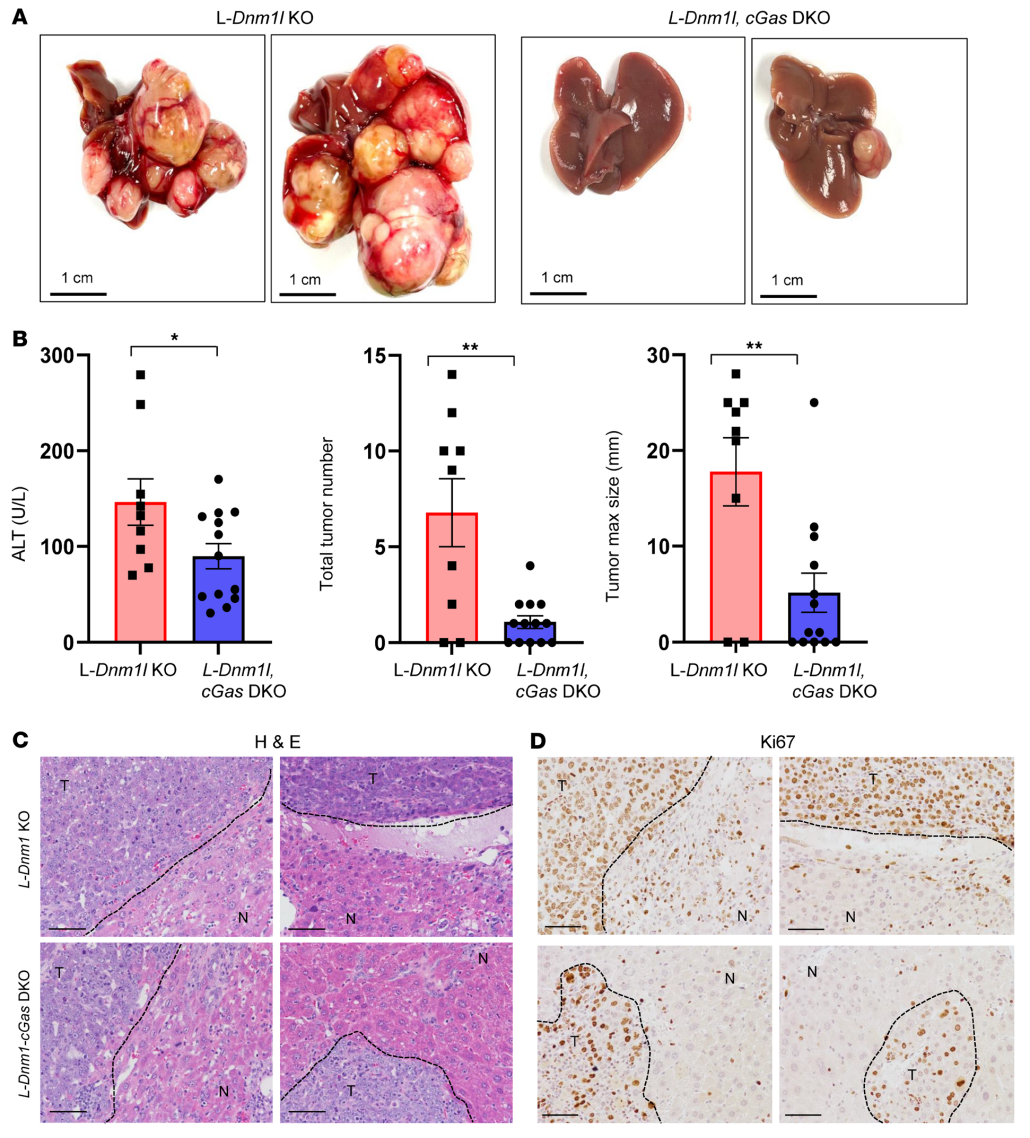
Loss of hepatic cGAS inhibits oncogene-driven liver tumor development. Sleeping beauty transposon (SB10) and c-MYC/YAP^S127A^ were delivered into 8-week-old male mice of indicated genotypes through hydrodynamic tail vein injection. Liver tissues and blood were collected 8 weeks after injection. (**A**) Representative gross mouse liver images of indicated genotypes. Scale bars: 1 cm. (**B**) Serum ALT activities, the number of tumors per mouse liver, and tumor size are quantified. All results are expressed as mean ± SE (*n* = 9–13). *P < 0.05, **P < 0.01; Student’s *t* test. (**C** and **D**) Representative H&E and IHC staining of Ki67 in livers from mice with specific genotypes. The black dotted line marks the boundary of tumor (T) and nontumor (N) areas. Scale bars: 100 μm.

We generated L-*Dnm1l–* and *cGas-*DKO mice, and these mice were either fed a chow diet for 15–18 months or injected with *c-MYC/YAP* and sleeping beauty plasmids. We found that these DKO mice at 15–18M exhibit decreased serum ALT levels, reduced tumor incidence and size, and improved histology compared with L-*Dnm1l*–KO mice (Supplemental Figure 24 and Supplemental Table 1). Similarly, the L-*Dnm1l–* and *cGas-*DKO mice also showed decreased serum ALT levels, fewer tumors, smaller tumor sizes, improved histology, and fewer Ki67^+^ cells compared with WT mice that received the oncogene injection (Figure 9).

## Discussion

Mitochondrial morphological changes have been observed in various cancers and are considered a crucial metabolic factor that supports cancer cell growth and metastasis (21–23). Human HCC tissues are generally found to have higher DRP1/MFN1 expression ratios, which was linked to a poor prognosis, and enhanced mitochondrial fission has been shown to promote HCC cell survival in vitro and in vivo via elevated ROS production (3, 10). These results led to a widely accepted notion that fragmented mitochondria promote the viability of cancer cells in nonpermissive conditions. However, there is still no verdict on the actual mitochondrial size in human HCC tumor tissues. Indeed, direct evidence from 2 independent studies investigating mitochondrial length from paired HCC tumor-peritumor tissues through EM drew conflicting conclusions (3, 24), indicating a strong heterogeneous metabolic feature of cancer cells in HCC. However, mitochondrial fusion was also found to support liver tumor growth (24). Another study revealed that metabolic reprogramming via mitochondrial elongation is favorable for HCC cell survival and adaptation to energy stress (25). In the present study, we also observed increased mitochondria size in *c-MYC/YAP*–induced HCC cells in mice. Therefore, the role of mitochondrial architecture changes in liver cancer progression is complicated, and it seems that both fragmented and elongated mitochondria can be protumorigenic in the liver.

In this study, we found that deletion of either mitochondrial fission protein DRP1 or the 2 fusion proteins MFN1 and MFN2 in the liver resulted in spontaneous tumor formation in mice, and this phenotype can be rescued by restoration of mitochondrial dynamic balance through triple depletion of DRP1, MFN1, and MFN2. These findings suggest that mitochondria stasis is the key to preserve normal cellular functions in the liver and prevent tumorigenesis. Unlike previous reports showing that the removal of *Mfn1* or *Mfn2* promoted liver tumor formation either under aging-associated spontaneous conditions for up to 24M or triggered by carcinogen exposure (9, 26, 27), our data suggested that single KO of either *Mfn1* or *Mfn2* is not sufficient to cause age-related spontaneous tumor formation in the liver up to 18M. Some differences between the published results and ours may stem from our L-*Mfn2* mice being younger (18M) compared with the older ones used (24M). Nonetheless, it is highly likely that the loss of 1 MFN protein may be compensated by the other. In this study, we found a small portion (29% incidence) of L-*Mfn1*, *Mfn2* DKO mice developed tumors at 15–18M; however, the tumor burden and size in these mice were significantly lower than L-*Dnm1l–*KO mice, suggesting that mitochondrial fission may play a more critical role than mitochondrial fusion in maintaining hepatocyte homeostasis and preventing tumorigenesis. As fragmented mitochondria are more readily removed by mitophagy, it is likely that the lower levels of mitophagy in L-*Dnm1l–*KO mice compared with L-*Mfn1*, *Mfn2* DKO mice would lead to increased accumulation of damaged mitochondria, elevated oxidative stress, DNA damage, genome instability, and, eventually, liver tumorigenesis.

One of the most intriguing findings of this study is the increased metabolism of pyrimidines observed in the livers of L-*Dnm1l–*KO mice. Mitochondria are essential in pyrimidine metabolism, particularly in de novo pyrimidine synthesis, as the key enzyme catalyzing the conversion of dihydroorotate to orotate, DHODH, is located within the mitochondrial inner membrane (28). Our current study found that increased DHODH levels in L-*Dnm1l*–KO mice were associated with megamitochondria and enhanced pyrimidine biosynthesis. The increased production of pyrimidine nucleotides, which are essential for DNA and RNA synthesis, may contribute to liver tumor development in L-*Dnm1l–*KO mice. Future research is needed to further explore the causal relationship between megamitochondria formation, increased DHOH levels, and how DHODH-mediated pyrimidine synthesis and metabolism promote liver cancer progression.

The activation of adaptive and innate immune responses is a key outcome of mitochondrial dysfunction, which has been implicated in tumor surveillance (29). The cGAS/STING pathway is an evolutionarily conserved DNA-sensing defense mechanism that was initially identified for its role in protecting cells against viral infections (30). Accumulating evidence recently revealed a dual function of cGAS/STING signaling in both tumor suppression and promotion (31). Acute activation of STING in early neoplastic cells leads to cell cycle arrest via senescence-associated secretory phenotype (SASP). Experiment data indicated that cGAS or STING deficiency impairs senescence and SASP responses, accelerates spontaneous immortalization, and promotes tumor development (32–35). However, chronic exposure to activated cGAS/STING signaling can lead cancer cells to lose downstream cell cycle regulators, fostering tolerance. Additionally, sustained activation of inflammatory signaling in this state exhausts effector immune cells and recruits immunosuppressive cells, collectively creating an immune-suppressive tumor microenvironment that facilitates tumor survival and immune evasion (31, 36–38). Here, we reported a remarkable upregulated cGAS/STING pathway in L-*Dnm1l*–KO mouse liver, which is associated with an increased number of senescent cells and elevated cell cycle inhibitors, and these phenotypes become more evident in 15–18M tumor-bearing mice. Highly elevated cGAS/STING protein expression was observed in oncogene-induced tumor regions of L*-Dnm1l–*KO mice. Most importantly, further deletion of hepatic *cGAS* reduced spontaneous and oncogene-induced liver tumor development in L-*Dnm1l*–KO mice, supporting the idea that the activated cGAS/STING pathway promotes liver tumorigenesis in L-*Dnm1l*–KO mice.

Another interesting finding from this study is that TKO mice, with reestablished mitochondrial fission/fusion stasis, were more resistant to oncogene-induced hepatic tumorigenesis than WT mice. This resistance is likely attributed to an enhanced SASP and reduced pyrimidine synthesis, as indicated by the presence of more senescent cells and lower pyrimidine metabolites in TKO mouse liver. The mitochondria in TKO mice are theoretically less active than those in WT mice due to the absence of both fission and fusion proteins, rendering them less responsive to external stress. Indeed, TKO hepatocytes have an increased number of mitochondria with loss or abnormal reorganization of cristae, leading to reduced mitochondrial oxygen consumption and respiration. While these changes might only moderately affect the survival of normal hepatocytes, they could significantly impact the growth of transformed tumor cells due to their higher energy needs.

In conclusion, we found that deletion of mitochondrial fission protein DRP1 in the liver resulted in spontaneous tumor formation in mice. Mitochondrial dynamics and stasis are critical to maintaining hepatic mitochondrial homeostasis, pyrimidine metabolism, and hepatocyte functions. L-*Dnm1l–*KO mice had elevated pyrimidine metabolism and an activated cGAS/STING signaling pathway, which is associated with increased DNA damage, senescence, and compensatory proliferation. These factors collectively accelerated tumorigenesis during aging or exposure to external oncogenes. This study lays the foundation for future drug development aimed at maintaining homeostasis of mitochondrial dynamics to preserve normal liver physiology and prevent cancer.

## Methods

### Sex as a biological variable.

Male animals have a higher incidence of HCC than female ones. Our study examined both male and female animals, and results are reported for both sexes.

### Animal experiments.

*Dnm1l*^fl/fl^ mice (C57BL/6/129); *Mfn1* and *Mfn2*^fl/fl^ mice (C57BL/6/129); and *Dnm1l, Mfn1,* and *Mfn2*^fl/fl^ mice (C57BL/6/129) were generated as described previously (39). *cGas*^fl/fl^ mice were purchased from The Jackson Laboratory (catalog 026554). All mice were crossed with albumin-Cre mice (Alb-Cre, C57BL/6J, The Jackson Laboratory) for 6 generations. To generate a model of diet-induced MASH, male 8-week-old DKO mice and matched WT mice were fed with a chow diet and a CDAHFD (choline-deficient, amino acid–defined, high-fat diet [45% fat] containing 0.1% methionine) for 6 weeks. All animals were specific pathogen free and maintained in a barrier rodent facility under standard experimental conditions. All procedures were approved by the IACUC of The University of Kansas Medical Center. Blood and liver tissues were collected at the indicated mouse age. Liver injury was determined by measuring serum ALT and AST. Liver sections and H&E staining were performed as described previously (40).

### Hydrodynamic injection mouse model.

Eight-week-old male L-*Dnm1l*–KO, TKO, and matched WT mice were used in this study. Hydrodynamic injection was performed as previously described (20). Briefly, 20 μg of pT3-EF1aH *c-MYC* and pT3-EF1aH *YAP S127A* along with 1.6 μg of pCMV-SB10 transposase at a ratio of 25:1 were diluted in Ringer’s solution at a volume equivalent to 10% of the mouse’s body weight (g/mL). The solution was filtered through a 0.22 μm filter (Millipore, GSWP04700) and injected into the lateral tail vein in 5–7 seconds. Liver tissues and blood were collected 8 weeks after injection.

### RNA extraction and amplification.

Total RNA was extracted from mouse livers using TRIzol reagent (15596-026, Ambion, Thermo Fisher Scientific). For RNA-seq analysis, total RNA was assessed for quality using a Qubit assay for quantification and Agilent TapeStation gel analysis for integrity. Sequencing libraries were constructed using 1 μg of total RNA using the NEBNext Ultra II Directional RNA Library Prep Kit for Illumina (New England Biolabs). The sequencing library construction process included mRNA purification by the poly(A) tails with poly(T) magnetic beads, fragmentation, strand-specific cDNA synthesis, end repair, 3′ end adenylation, adapter ligation, and PCR amplification. The constructed sequencing libraries were validated and quantified with Qubit and TapeStation assays. The sequencing library preparations were pooled together equally by nanogram amount, and the nanomolar concentration of the pool was verified with an Illumina KAPA Library Quantification qPCR assay (Roche). Each library was indexed with a barcode sequence and sequenced in a multiplexed fashion. An Illumina NextSeq 550 system was used to generate single-end, 75-base sequence reads from the libraries. Base calling was carried out by the instrument’s Real-Time Analysis software. The base call files (BCL files) were demultiplexed and converted to compressed FASTQ files by bcl2fastq2.

### RNA-seq data analysis.

RNA-seq data were analyzed using our previously established technical pipeline (41, 42). Briefly, we used HISAT2 v.2.1.0.13 to map the high-quality reads to the mouse reference genome (GRCm38.90). Quantification of the gene expression was generated with HTSeq-counts v0.6.0. Significant differentially expressed genes were generated with the R package DESeq2. Statistical significance was calculated by adjusting the *P* values with Benjamini-Hochberg FDR (43). The clustered heatmaps of the cGAS/STING pathway genes were plotted with ClustVis (44). PCA and volcano plots were prepared with R. Pathway analysis was performed using Ingenuity Pathway Analysis. We analyzed the enrichment of up- and downregulated genes among DRP1 KO and TKO groups compared with the WT group. Genes with a log_2_ fold change > 1.5 or < −1.5 with an FDR-corrected *q* value < 0.05 were used to perform the analysis.

### Liver metabolomics analysis.

Metabolomics analysis was performed by Metabolon, resulting in a dataset comprising 907 compounds of known identity (biochemicals). After log transformation and imputation of missing values using the minimum observed value for each compound, Welch’s 2-sample *t* test was applied to identify biochemicals that significantly differed between groups. A summary of the numbers of biochemicals that achieved statistical significance (*P* ≤ 0.05) and those approaching significance (0.05 < *P* < 0.10) is provided in the Supporting Data Values file.

### Liquid chromatography–mass spectrometry.

Twenty to forty milligrams of frozen liver tissue samples was weighed and kept in prechilled 2 mL Eppendorf tubes with beads and ground with a CryoMill (Retsch, NC1216630) at liquid nitrogen temperature and 20 Hz for 2 min. The ground samples were extracted by adding 40× 40:40:20 methanol:acetonitrile:water solution at –20°C with 0.1 M formic acid, followed by vortexing and centrifugation at 16,000*g* for 10 min at 4°C. Forty-four microliters of 15% (m/v) NH_4_HCO_3_ was added to 500 μL of the transferred extract to neutralize the acid. The samples were centrifuged at 16,000*g* for 10 min to remove the protein precipitate. Then, 300 μL of the supernatant was collected into a new tube for LC-MS. LC-MS was performed at the Rutgers Cancer Institute Metabolomics Shared Resource as previously described (45). The metabolite features were extracted in El-MAVEN with a mass accuracy window of 5 ppm. The metabolite annotation is based on accurate mass and retention time that matches the in-house library.

### Western blot analysis.

Total liver lysates were prepared using RIPA buffer (1% NP-40, 0.5% sodium deoxycholate, and 0.1% SDS). Proteins (30 μg) were separated by 8%–12% SDS-PAGE before transfer to a PVDF membrane. Membranes were probed using indicated primary and secondary antibodies and developed with SuperSignal West Pico Plus Chemiluminescent Substrate (Thermo Fisher Scientific, 34579) and Immobilon Western Chemiluminescent HRP Substrate (Millipore, WBKLS0500). All the primary and secondary antibodies used in this study are listed in Table 3.

### EM.

Fine-cut liver tissues were fixed with 2% glutaraldehyde in 0.1 mol/L phosphate buffer (pH 7.4) followed by 1% OsO_4_. After dehydration, thin sections were stained with uranyl acetate and lead citrate for observation under a JEM 1016CX electron microscope (JEOL). Images were acquired digitally.

### Histological staining.

A standard IHC procedure was applied in this study. Briefly, paraffin-embedded tissue sections were incubated with primary antibodies at 4°C overnight after deparaffinization and heat-induced antigen retrieval in citrate buffer. Sections were then washed and incubated with secondary antibodies for 1 h at 37°C and developed using ImmPACT NovaRED HRP substrate (Vector Labs, SK-4805). Tissues were counterstained with hematoxylin. The number of IHC positively stained cells was counted from 15 different fields (×200) in a double-blinded fashion. For Sirius red staining, paraffin-embedded liver tissue sections were dewaxed and rehydrated followed by staining sections with Sirius red solution.

The human HCC and normal liver tissue array slide (Biomax, LV1002) was immunostained for DRP1.

### β-Gal staining.

Liver cryosections were used for β-Gal staining following the manufacturer’s instructions (Cell Signaling Technology, 9860). Briefly, liver cryosections were fixed with 1× Fixative Solution for 15 min at room temperature and rinsed 2 times with 1× PBS. β-Gal Staining Solution was applied for a 37°C overnight incubation until a desired color was reached. Tissue sections were imaged immediately thereafter.

### Statistics.

All experimental data are expressed as mean ± SE and were subjected to 1-way ANOVA analysis with Bonferroni’s post hoc test or 2-tailed Student’s *t* test where appropriate. Mean ± SD was used for the quantitative data. A *P* value less than 0.05 was considered significant.

### Study approval.

All procedures were approved by the IACUC of The University of Kansas Medical Center (protocol IPROTO2024-387).

### Data availability.

The RNA-seq data have been deposited in NCBI’s GEO (GSE295255) and are publicly available. Values for all data points in graphs are reported in the Supporting Data Values file.

## Author contributions

WXD conceived and supervised the project. XM, XW, MN, CZ, ZP, WL, JY, XS, LM, SL, WC, WXZ, and HMN performed experiments and analyzed the data. XM, HMN, and WXD conceived and designed the experiments. WXZ and HS provided key reagents and discussed the manuscript. WL, ZP, and XM analyzed the RNA-seq data. XM and WXD analyzed data and wrote the manuscript.

## Funding support

This work is the result of NIH funding, in whole or in part, and is subject to the NIH Public Access Policy. Through acceptance of this federal funding, the NIH has been given a right to make the work publicly available in PubMed Central.

- National Institute on Alcohol Abuse and Alcoholism awards R37 AA020518, R21 AA030617, and R01 AA031230 (to WXD).
- National Cancer Institute, R01 CA224550 (to WXZ).
- National Institute of Diabetes and Digestive and Kidney Diseases, R01 DK124612, R01 DK106540, and R01 DK124612 (to WL).
- National Institute of Diabetes and Digestive and Kidney Diseases, R01DK134737 (to HMN).
- National Institute of Environmental Health Sciences, R01 ES033623 (to WL).
- The University of Kansas Cancer Center, William Warner Abercrombie endowment (to WXD).

## Supplementary Material

Supplemental data

Unedited blot and gel images

Supporting data values

## Figures and Tables

**Table 3:**
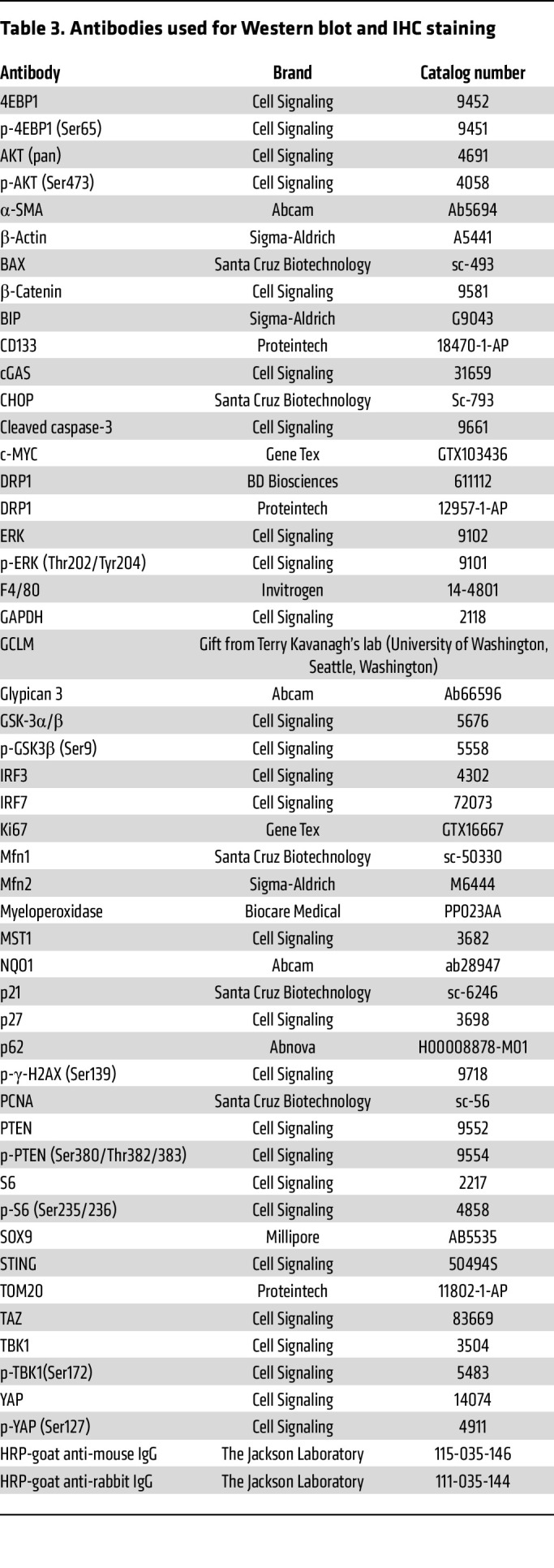
Antibodies used for Western blot and IHC staining

**Table 2:**
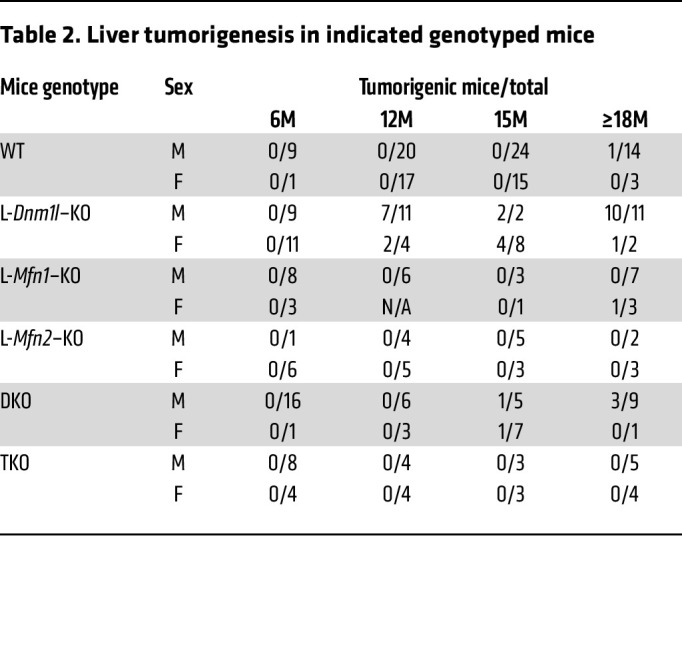
Liver tumorigenesis in indicated genotyped mice

**Table 1:**
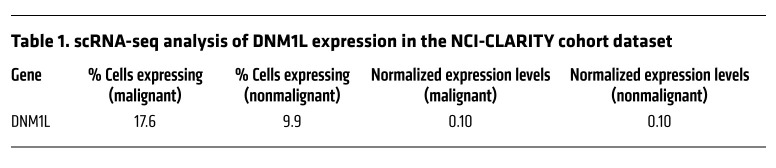
scRNA-seq analysis of DNM1L expression in the NCI-CLARITY cohort dataset

## Version 1 12/16/2025

In-Press Preview

## Version 2 02/16/2026

Electronic publication
