# Supplementary material for: Genetic disruption of mitochondrial dynamics and stasis leads to liver injury and tumorigenesis
Source: J Clin Invest. 2025 Dec 16;136(4):e194441. doi: 10.1172/JCI194441 (PMC12904713; doi:10.1172/JCI194441)
Supplement: Supplemental data [file jci-136-194441-s195.pdf]

Genetic Disruption of Mitochondrial Dynamics and Stasis Leads to Liver Injury and Tumorigenesis

Xiaowen Ma[1#], Xiaoli Wei[1], Mengwei Niu[1], Chen Zhang[1], Zheyun Peng[2], Wanqing Liu[2,3], Junrong Yan[4], Xiaoyang Su[4], Lichun Ma[5,6], Shaolei Lu[7], Wei Cui[8], Hiromi Sesaki[9], Wei-Xing Zong[4], Hong-Min Ni[1], and Wen-Xing Ding[1,8*]

Supplemental Figure Legend

Supplemental Figure 1. Gene expression correlation of mitochondrial dynamic genes with other pathways.

Gene expression data from non-tumor (n=50) and HCC tumor tissues (n=374) in the TCGA-LIHC dataset was used for heatmap and gene-gene expression correlation analysis. The heatmap and correlation plots display the expression of mitochondrial dynamic genes and their correlation with basic mitochondrial genes (A) and oncogenes (B).

Supplemental Figure 2. Gene expression correlation of mitochondrial dynamic genes with other pathways.

Gene expression data from non-tumor (n=50) and HCC tumor tissues (n=374) in the TCGA-LIHC dataset was used for heatmap and gene-gene expression correlation analysis. The heatmap and correlation plots illustrate the expression of mitochondrial dynamic genes and their correlation with pyrimidine metabolism genes (A) and cGAS-interferon pathway genes (B).

Supplemental Figure 3. Increased hepatic α-SMA levels in L-*Dnm1l* KO but not TKO mice.

Western blot analysis of total liver lysates of indicated mouse genotypes at 2M (A), 6M (B) and 12M (C).

Supplemental Figure 4. Decreased mitochondria oxygen consumption rate (OCR) and state 3 respiration in L-*Dnm1l* KO but not TKO mouse liver mitochondria.

(A) Hepatic mitochondria were isolated for bioenergetics analysis using Seahorse Bioscience XF analyzer (n=3-5) (B). Quantification of state 3 respiration from (A). Data are shown as means± SEM (n=3-5). ADP, adenosine diphosphate; FCCP, carbonyl cyanide p-trifluoromethoxyphenylhydrazone; OCR, oxygen consumption rate, AIF, apoptosis-inducing factor.

Supplemental Figure 5. L-Dnm1l KO mouse livers have decreased mitophagy which was rescued in TKO mice.

(A) Representative EM images of livers from 2-month-old mice of the indicated genotypes. Black arrows indicate mitochondria enclosed by an autophagosome. Red arrow indicates megamitochondria. Bar: 500 nm. The TKO image contains overlapping areas and was cropped from two different regions of the same sample, as shown in Figure 2D. (B) Quantification of mitophagy from EM images. Data shown are dot plot of the mitophagosomes that contain mitochondria (n=31-46 images of 3 different mouse livers).

Supplemental Figure 6. Characterization of overexpression of MFN2 in mouse livers.

Male 2-3 months old C57BL/6J mice were either injected with Ad-null or Ad-Mfn2 (iv, 2x10^9 PFU/mouse) for 10 days. (A) Total liver lysates were analyzed by western blot. (B) Representative immunofluorescence images of TOM20 staining in the livers. Nuclei were stained with Hoechst 33342. Arrows indicate megamitochondria. (C) Serum ALT levels were quantified. Data are presented as means ± SE (n=3). * p<0.05, Student's t-test. (D) Representative H&E staining of liver tissues.

Supplemental Figure 7. L-Mfn1, Mfn2 DKO mice are resistant to CDAHFD-induced metabolic dysfunction-associated steatohepatitis (MASH).

Male 2 months old Alb Cre- *Mfn1, Mfn2* flox/flox mice and Alb Cre+, *Mfn1, Mfn2* flox/flox mice were fed either with a control chow diet or CDAHFD for 6 weeks. (A) Serum ALT levels, (B) hepatic triglyceride (TG) and (C) hepatic cholesterol levels were quantified (n=7). (D-E) Representative images

of H &E and Sirius Red staining of liver sections are shown. (F) Total liver lysates were subjected to western blot analysis. (G) Total hepatic RNA was extracted and subjected to qPCR analysis. Data are presented as means ± SE (n=7). * $p<0.05$, Two-way analysis of variance analysis with Bonferroni's post hoc test.

Supplemental Figure 8. L-*Dnm1l* KO mice but not DKO and TKO mice have increased activation of cGAS-STING-interferon pathway in the liver.

(A) Principal component analysis (PCA) of RNA-seq dataset of 6M mice. (B) Heatmap of interferon and cGAS-STING pathway involved genes from the RNA-seq dataset. (C) Western blot analysis from total liver lysates or tumor and non-tumor tissues of indicated mice at 18M. (D) Western blot analysis of total liver lysates from mice of indicated genotypes at 18M. T: tumor, NT: non-tumor.

Supplemental Figure 9. Loss of liver *Dnm1l* promotes endoplasmic reticulum (ER) stress and immune cell infiltration in mouse livers, which are suppressed in TKO mice.

(A-B) Representative immunohistochemistry staining of F4/80 and MPO from indicated mouse liver. At least 5 fields were quantified from each mouse (n=3 mice). (C) Western blot analysis of total liver lysate. All results are expressed as means± SD. *$p<0.05$, **$p<0.01$, ***$p<0.001$; One-Way ANOVA analysis with Bonferroni's post hoc test.

Supplemental Figure 10. IRF7-positive cells are closely localized in areas enriched with F4/80-positive macrophages.

Representative immunohistochemistry staining of F4/80 and IRF7 from 2-month-old L-*Dnm1l* KO mice. Arrows indicate IRF7 and F4/80-positive cells.

Supplemental Figure 11. Liver cell type gene expression signature in WT, L-*Dnm1l* KO, DKO and TKO mice.

(A) Pathway analysis of signature gene expression of hepatocytes, HSC and Kupffer cell/macrophage of RNA-seq dataset of 2M (A) and 6M (B) mice of indicated genotypes

Supplemental Figure 12. Changes of hepatic DHODH in L-*Dnm1l* KO mice.

(A) Total liver lysates from 2-month-old WT, L-*Dnm1l* KO, and L-*Dnm1l, Mfn1, Mfn2* TKO mouse livers were subjected to western blot analysis. (B) Total liver lysates from 2-month-old WT, L-*Dnm1l* KO, and L-*Dnm1l, cGAS* DKO mouse livers were subjected to western blot analysis.

Supplemental Figure 13. Mitochondria complex assembly gene expression changes in L-*Dnm1l* KO, DKO and TKO mice.

Heatmap of mitochondria complex assembly genes from the RNA-seq dataset of 2M and 6M mouse livers of indicated genotypes.

Supplemental Figure 14. Mitochondria cristae architecture and metabolic transporter gene expression changes in L-*Dnm1l* KO, DKO and TKO mice. Heatmap of mitochondria cristae architecture and metabolic transporter genes from the RNA-seq dataset of 2M and 6M mouse livers of indicated genotypes.

Supplemental Figure 15. Mitochondria transcription and protein import gene expression changes in L-*Dnm1l* KO, DKO and TKO mice.

Heatmap of mitochondria transcription and protein import genes from the RNA-seq dataset of 2M and 6M mouse livers of indicated genotypes.

Supplemental Figure 16. Mitochondria mRNA/mRNA/rRNA modification and processing, proteostasis and mitophagy, TRNA amino acid synthetase and UQ synthesis gene expression changes in L-*Dnm1l* KO, DKO and TKO mice. Heatmap of mitochondria mRNA/mRNA/rRNA modification and processing, proteostasis and mitophagy, TRNA amino acid synthetase and UQ synthesis genes from the RNA-seq dataset of 2M and 6M mouse livers of indicated genotypes.

Supplemental Figure 17. Mitochondria Ribosome subunit assembly factors, mitochondria

translation, and mtDNA replication gene expression changes in L-*Dnm1l* KO, DKO and TKO mice.

Heatmap of mitochondria Ribosome subunit assembly factors, mitochondria translation, and mtDNA replication genes from the RNA-seq dataset of 2M and 6M mouse livers of indicated genotypes.

Supplemental Figure 18. Liver adenoma cells in L-*Dnm1l* KO mice do not express DRP1 protein.

(A) Total liver lysates from normal, tumor and tumor adjacent normal tissues from 18-month-old WT, L-*Dnm1l* KO, and L-*Dnm1l, Mfn1, Mfn2* TKO mouse livers were subjected to western blot analysis. (B) Representative images of IHC staining of DRP1 from 18-month-old WT and L-*Dnm1l* KO mice. Arrows indicate DRP1 positive immune and endothelial cells. CV: central vein; PV: portal vein. The red line divides normal (N) and tumor tissues (T).

Supplemental Figure 19. Liver cell senescence staining in L-*Dnm1l* KO, DKO and TKO mice.

(A-B) Representative images of β-Galactosidase (β-Gal) staining using liver cryosections of 2M and 18M mice of indicated genotypes (n=2 mice in each group). All results are expressed as means± SD. *$p<0.05$, **$p<0.01$, ***$p<0.001$; One-Way ANOVA analysis with Bonferroni's post hoc test.

Supplemental Figure 20. DRP1 IHC staining in oncogene-driven tumor sections of L-*Dnm1l* KO and WT mice.

Sleeping beauty transposon (SB10) and *c-MYC/YAP*^{S127A} were delivered into 8-week-old male mice of indicated genotypes through hydrodynamic tail vein injection. Liver tissues and blood were collected 8 weeks post-injection. Representative IHC DRP1 staining images are shown.

Arrows indicate DRP1 positive immune cells. N: normal, T: Tumor. Bar: 100 μm.

Supplemental Figure 21. Expression of mitochondrial proteins in oncogene-driven tumor sections of L-*Dnm1l* KO, TKO and WT mice.

Sleeping beauty transposon (SB10) and *c-MYC/YAP*^{S127A} were delivered into 8-week-old male mice of indicated genotypes through hydrodynamic tail vein injection. Liver tissues were collected 8 weeks post-injection. (A) Western blot analysis from tumor and non-tumor tissues of indicated mice. (B) Densitometry analysis from (A). All results are expressed as means± SD (n=3-5). T: tumor, NT: non-tumor. OM: outer mitochondrial membrane, IM: inner mitochondrial membrane.

Supplemental Figure 22. TOM20 staining in oncogene-driven tumor sections of L-*Dnm1l* KO and WT mice.

Sleeping beauty transposon (SB10) and *c-MYC/YAP*^{S127A} were delivered into 8-week-old male mice of indicated genotypes through hydrodynamic tail vein injection. Liver tissues and blood were collected 8 weeks post-injection. (A) Representative immunofluorescence images of TOM20 staining are shown. (B) and (C) are enlarged photographs from the boxed areas. Arrow denotes fragmented mitochondria. Arrow heads denote large size mitochondria. Bar: 20 μm.

Supplemental Figure 23. Activated cGAS-STING pathway in oncogene-driven tumor sections of L-*Dnm1l* KO and WT mice.

Sleeping beauty transposon (SB10) and *c-MYC/YAP*^{S127A} were delivered into 8-week-old male mice of indicated genotypes through hydrodynamic tail vein injection. Liver tissues and blood were collected 8 weeks post-injection. Western blot analysis from tumor and non-tumor tissues of indicated mice. T: tumor, NT: non-tumor.

Supplemental Figure 24. Loss of hepatic cGAS inhibits liver tumorigenesis in L-*Dnm1l* KO mice.

L-*Dnm1l* KO and L-*Dnm1l, cGAS* DKO mice were fed with a regular chow diet and liver tissues were harvested at indicated time. (A) Representative gross liver image. (B) Serum ALT activities and quantification of liver tumor numbers and size. All results are expressed as means± SEM (n=9-11). *p<0.05, Student's t-test compared to corresponding L-*Dnm1l KO* group. (C) Representative H& E staining of liver tissues of L-Dnm1l KO and L-Dnm1l, cGAS DKO mice.

Supplemental Figure 1

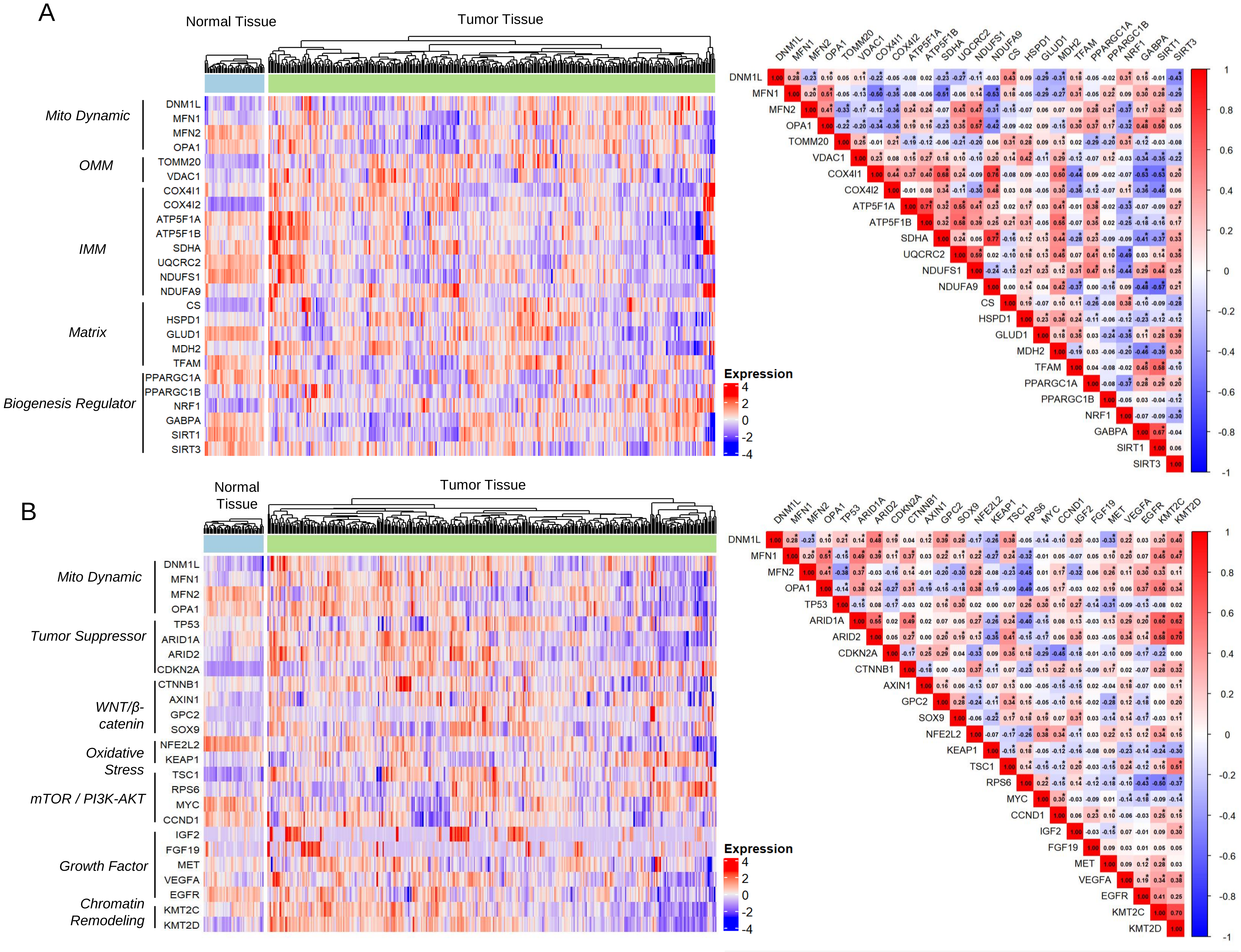

Supplemental Figure 2

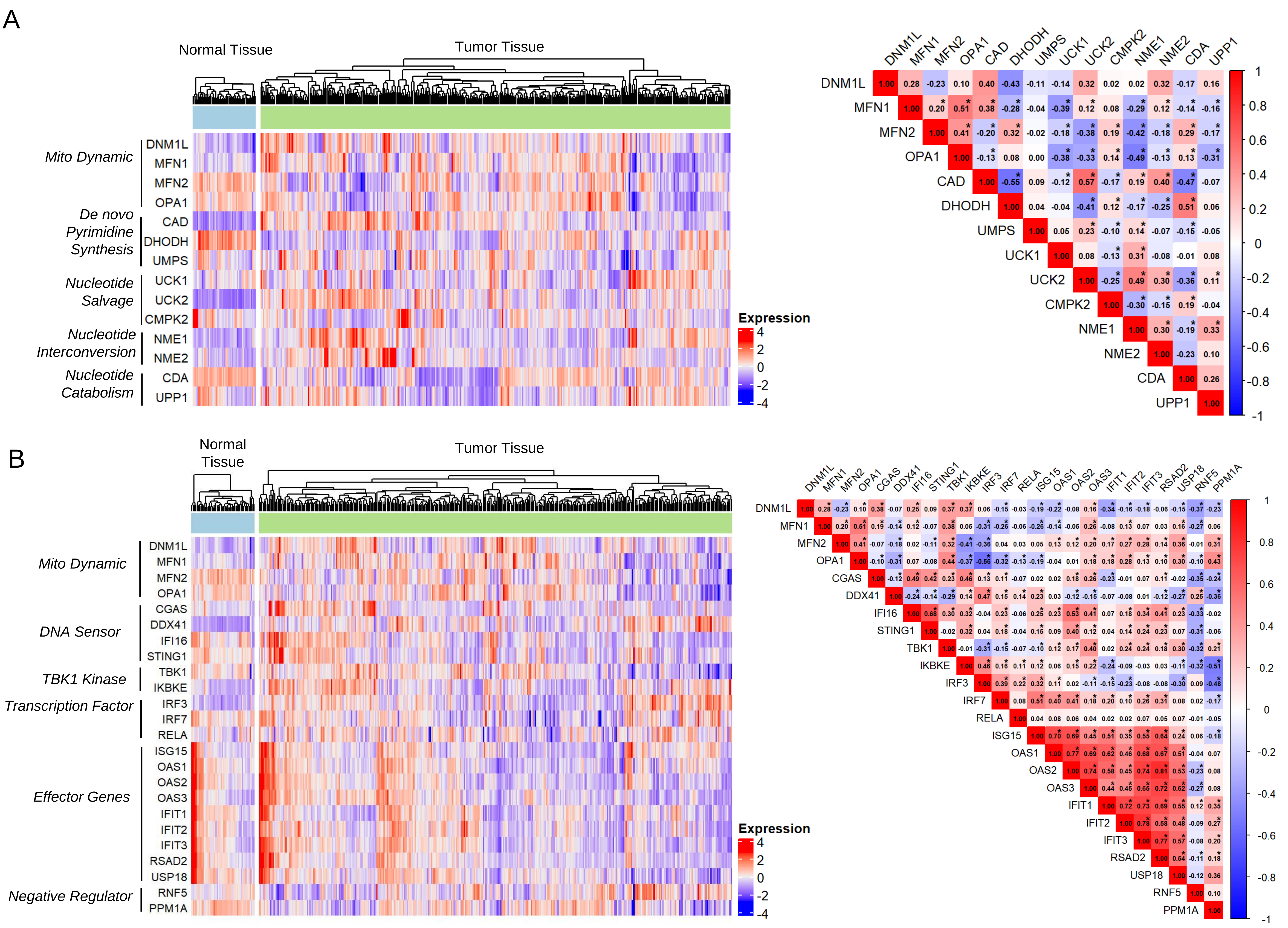

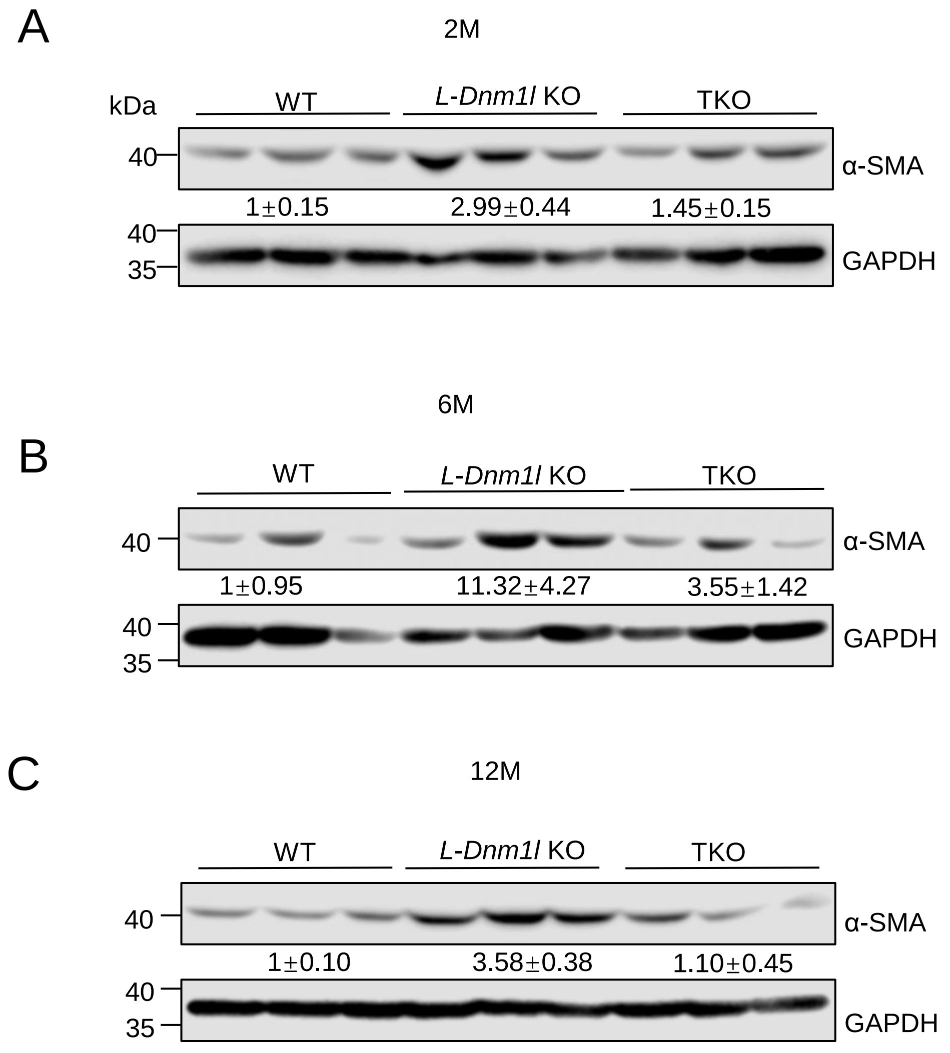
A
2M
kDa
WT
L-Dnm1l KO
TKO
40
α-SMA
1±0.15
2.99±0.44
1.45±0.15
40
35
GAPDH
B
6M
WT
L-Dnm1l KO
TKO
40
α-SMA
1±0.95
11.32±4.27
3.55±1.42
40
35
GAPDH
C
12M
WT
L-Dnm1l KO
TKO
40
α-SMA
1±0.10
3.58±0.38
1.10±0.45
40
35
GAPDH

Supplemental Figure 4

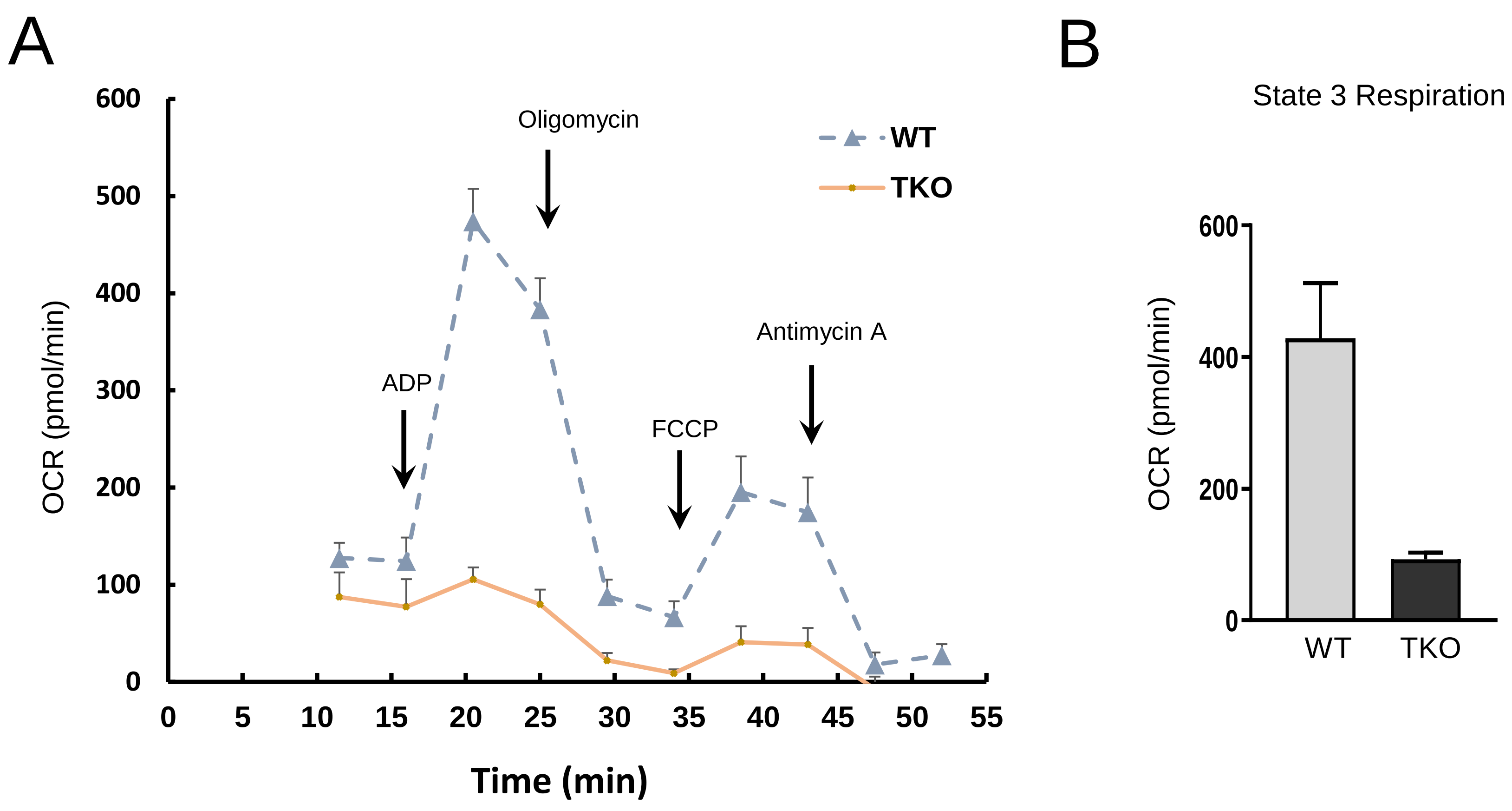

Supplemental Figure 5

A

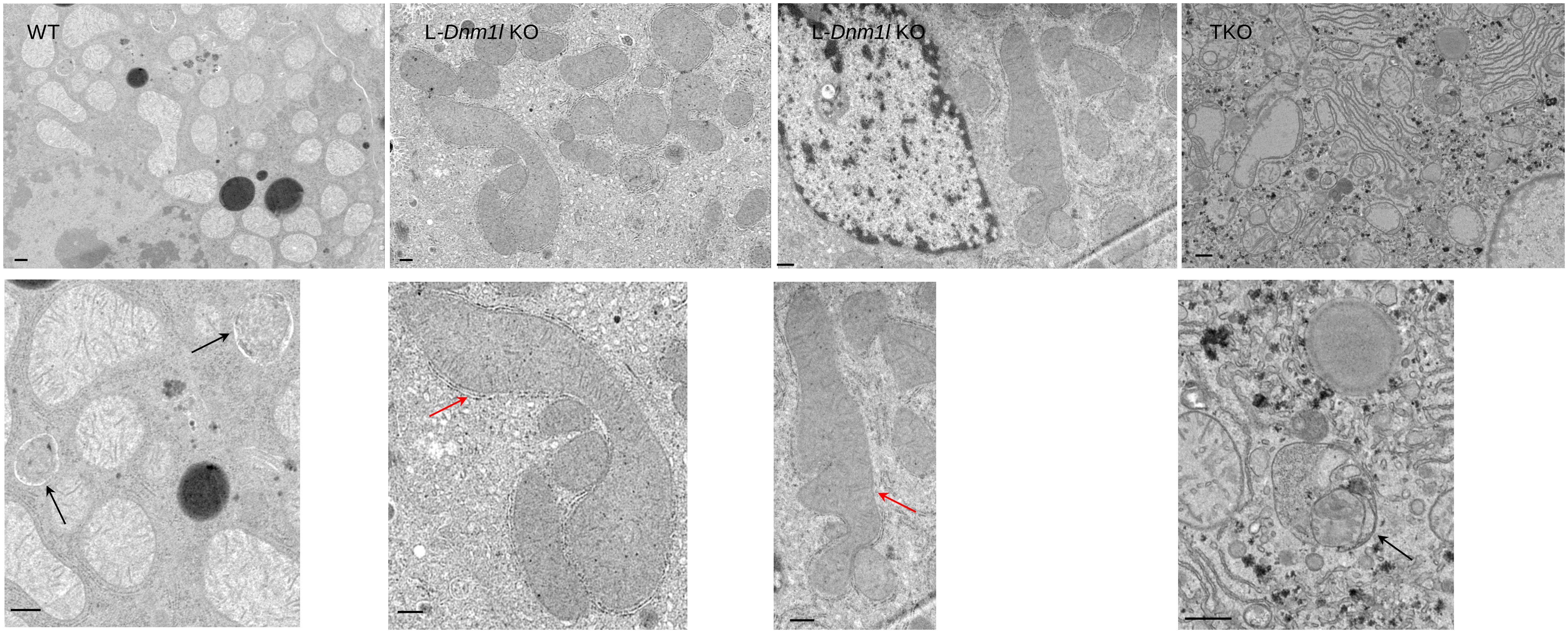

B

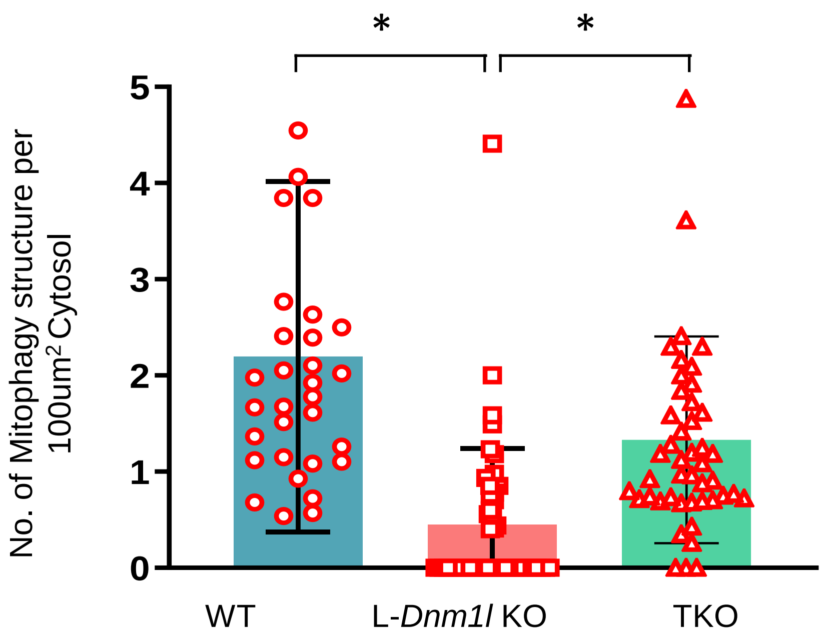

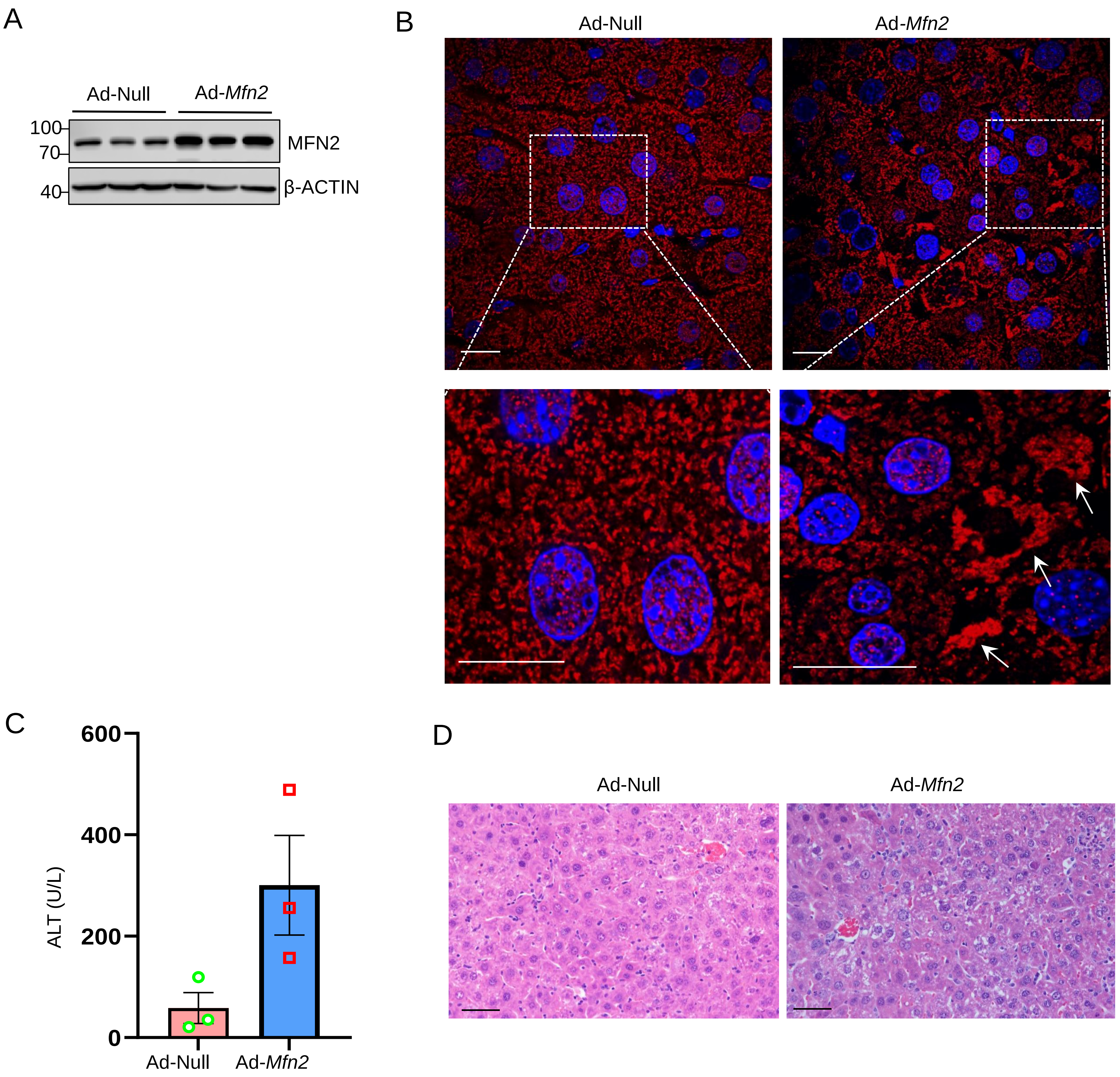
A
Ad-Null
Ad-*Mfn2*
100
70
40
MFN2
β-ACTIN
B
Ad-Null
Ad-*Mfn2*
C
600
400
200
0
ALT (U/L)
Ad-Null
Ad-*Mfn2*
D
Ad-Null
Ad-*Mfn2*

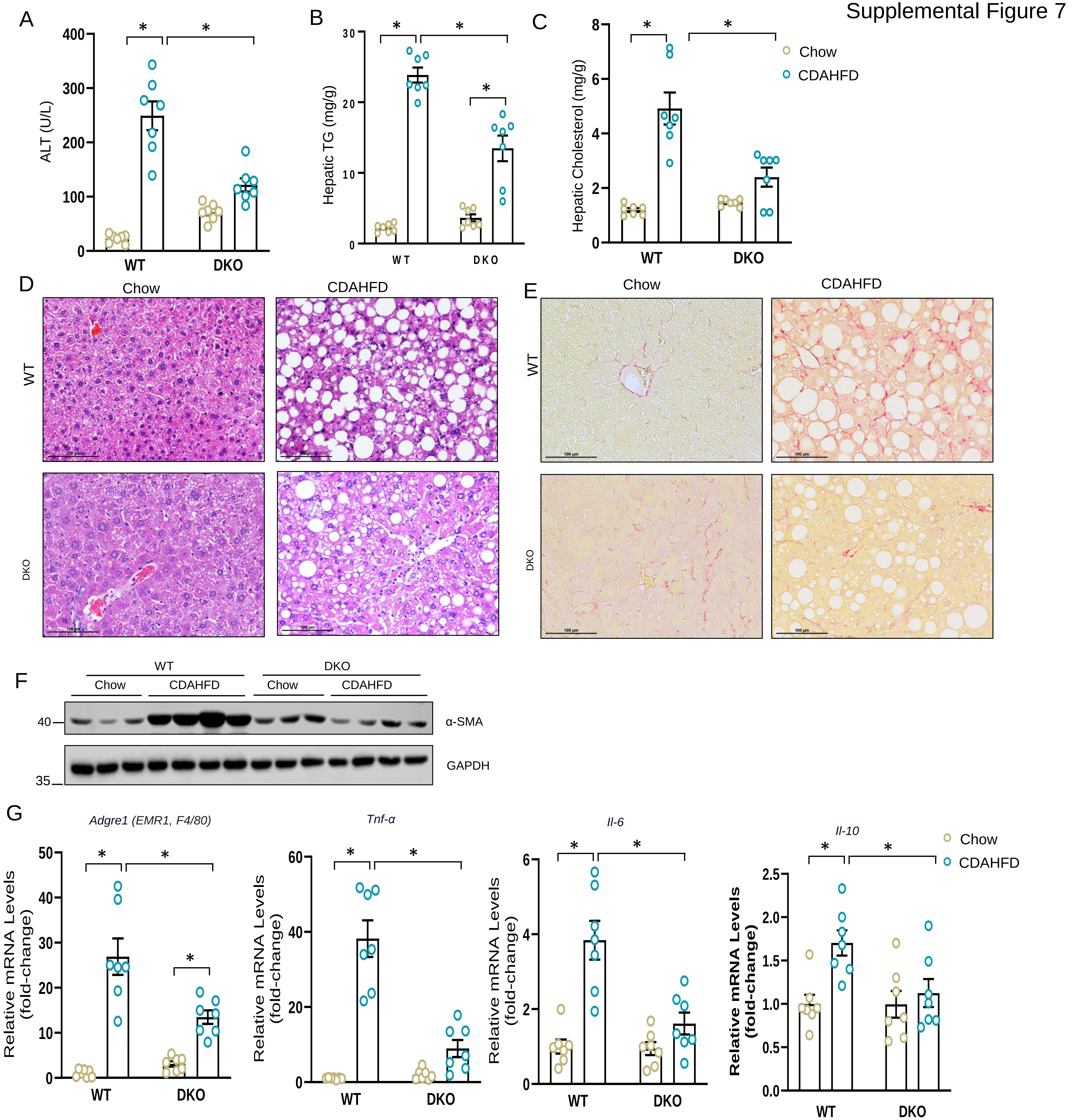
A
ALT (U/L)
400
300
200
100
0
WT
DKO
*
B
Hepatic TG (mg/g)
30
20
10
0
C
Hepatic Cholesterol (mg/g)
8
6
4
2
Chow
CDAHFD
D
E
WT
DKO
100 μm
F
α-SMA
GAPDH
40
35
G
Adgre1 (EMR1, F4/80)
Tnf-α
Il-6
Il-10
Relative mRNA Levels (fold-change)
50
40
60
2.5
2.0
1.5
1.0
0.5
0.0

Supplemental Figure 8

A

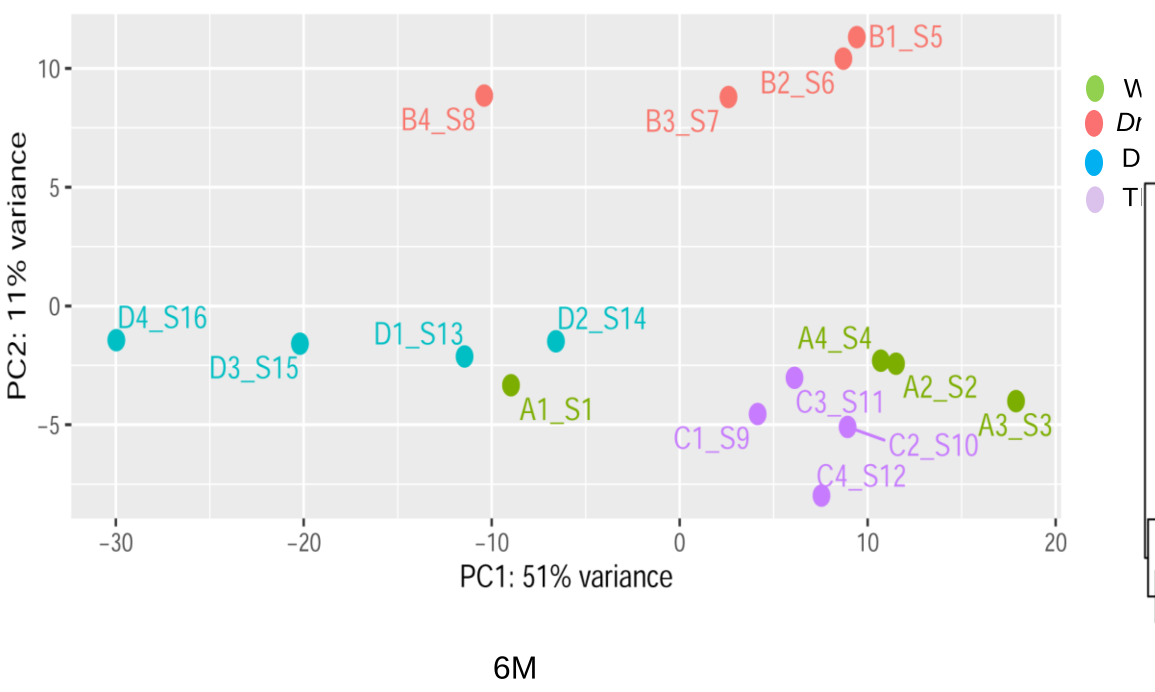

B

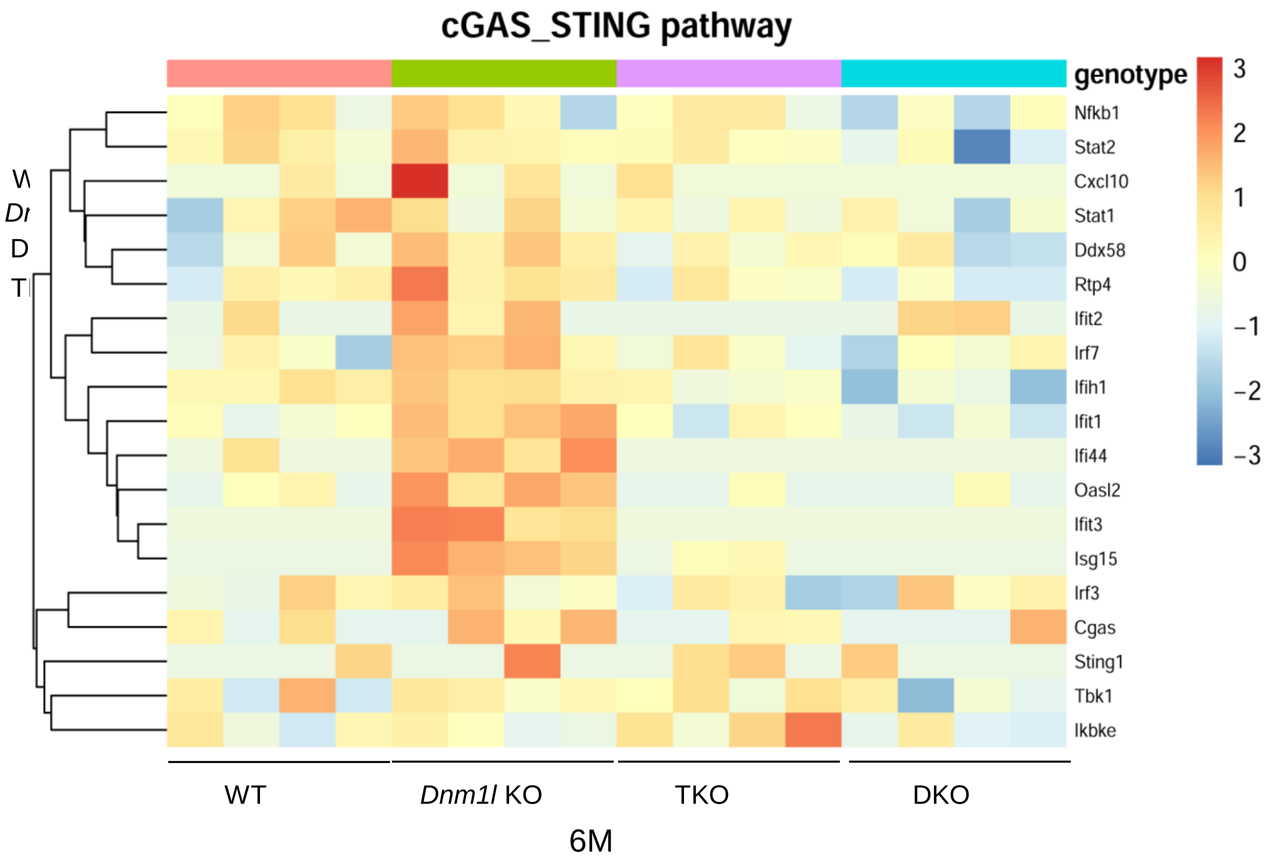

C

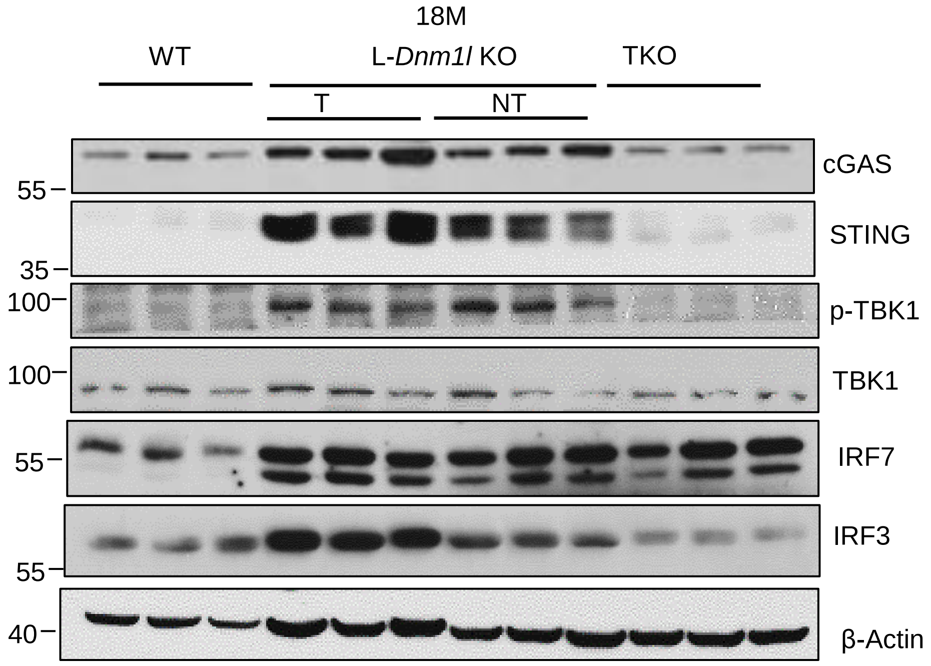

D

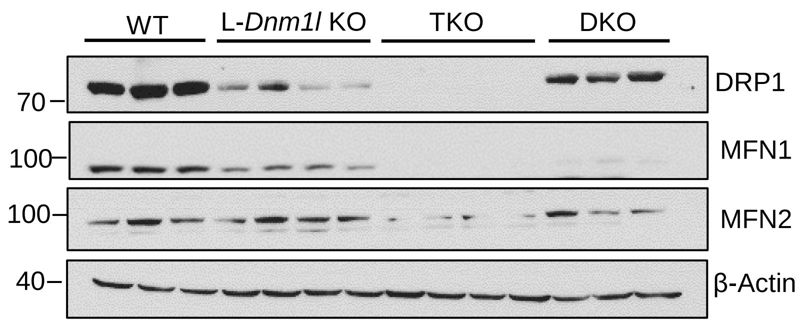

Supplement Figure 9

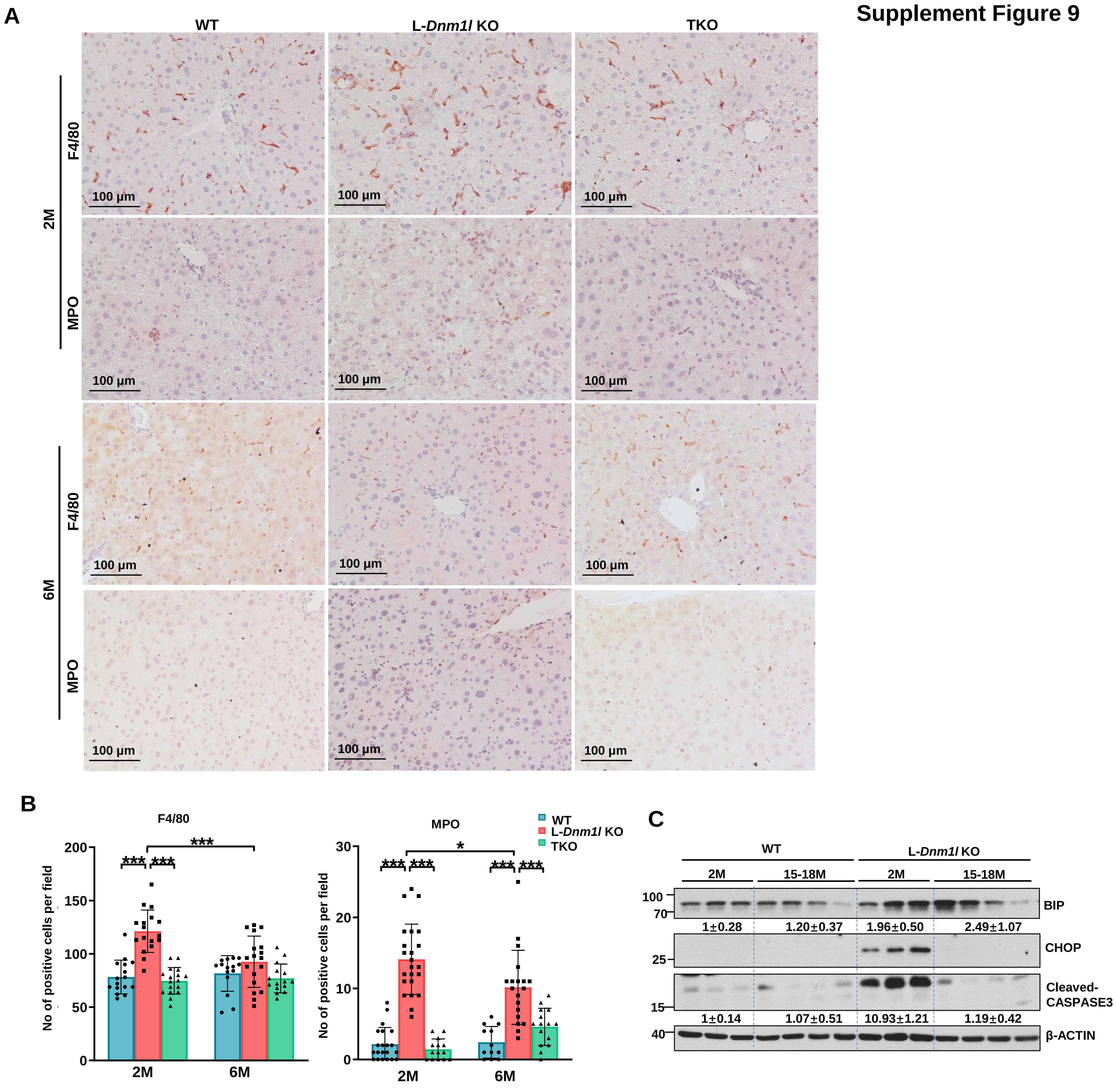

Supplemental Figure 10

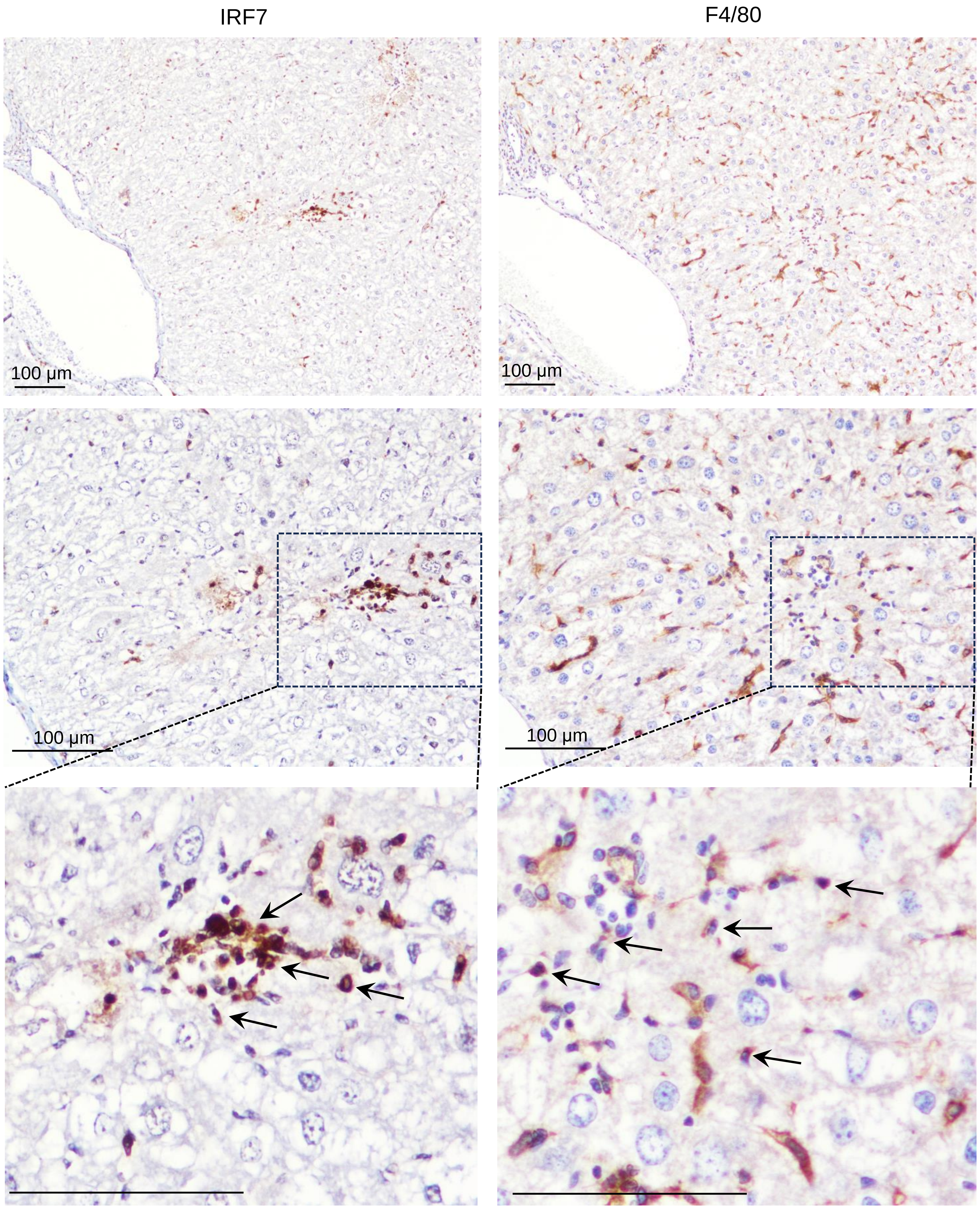

Supplemental Figure 11

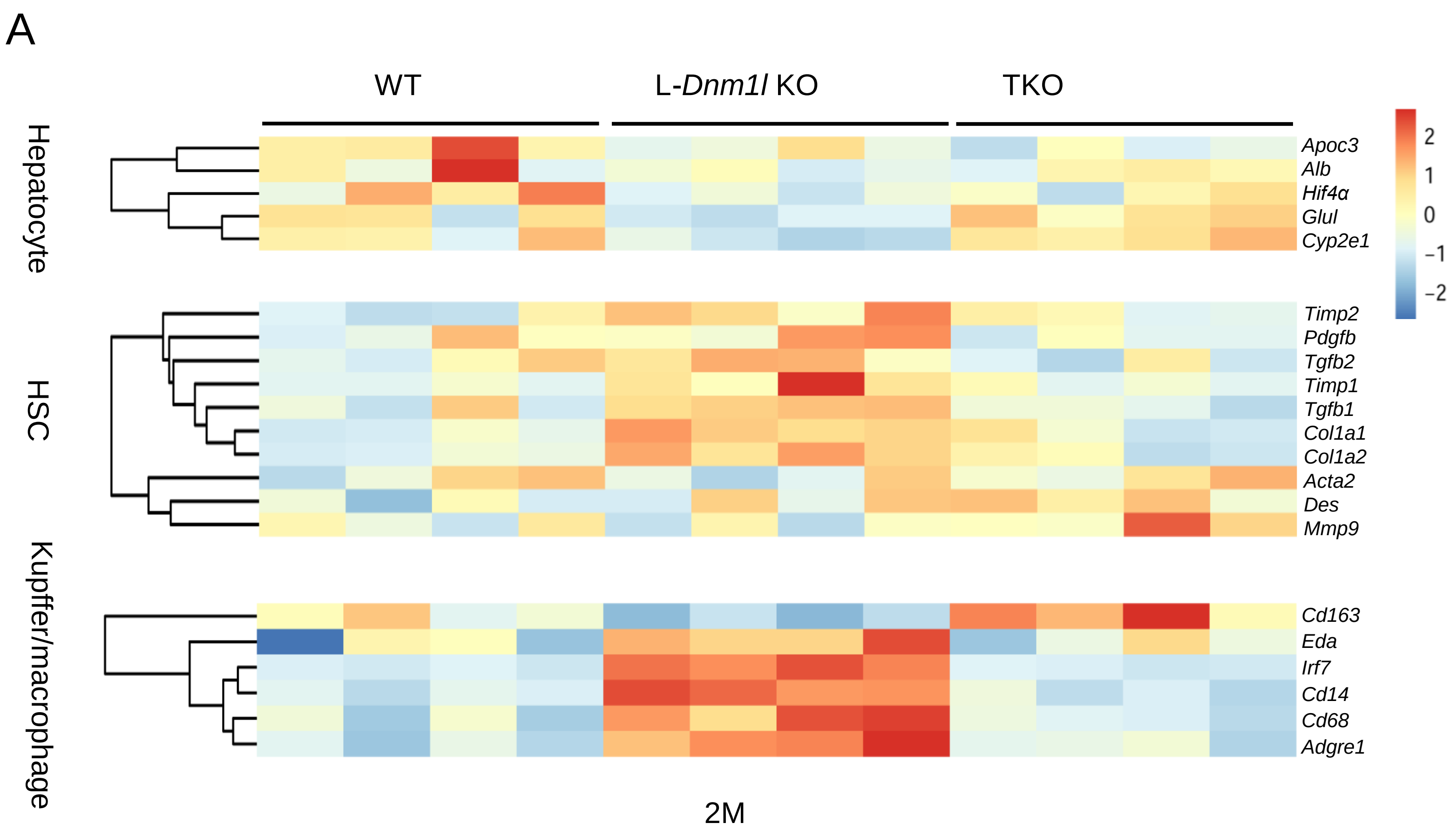

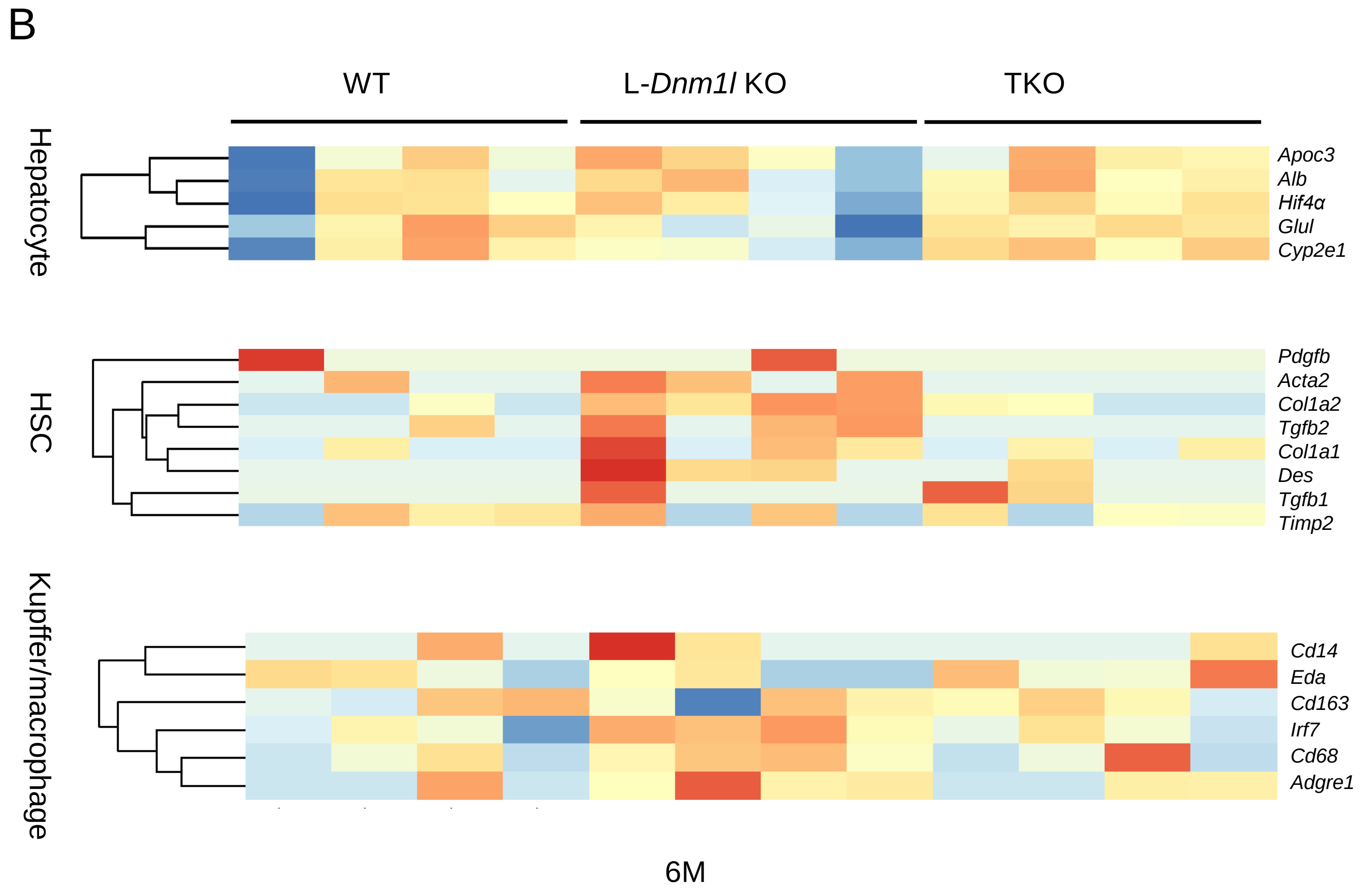

Supplemental Figure 12

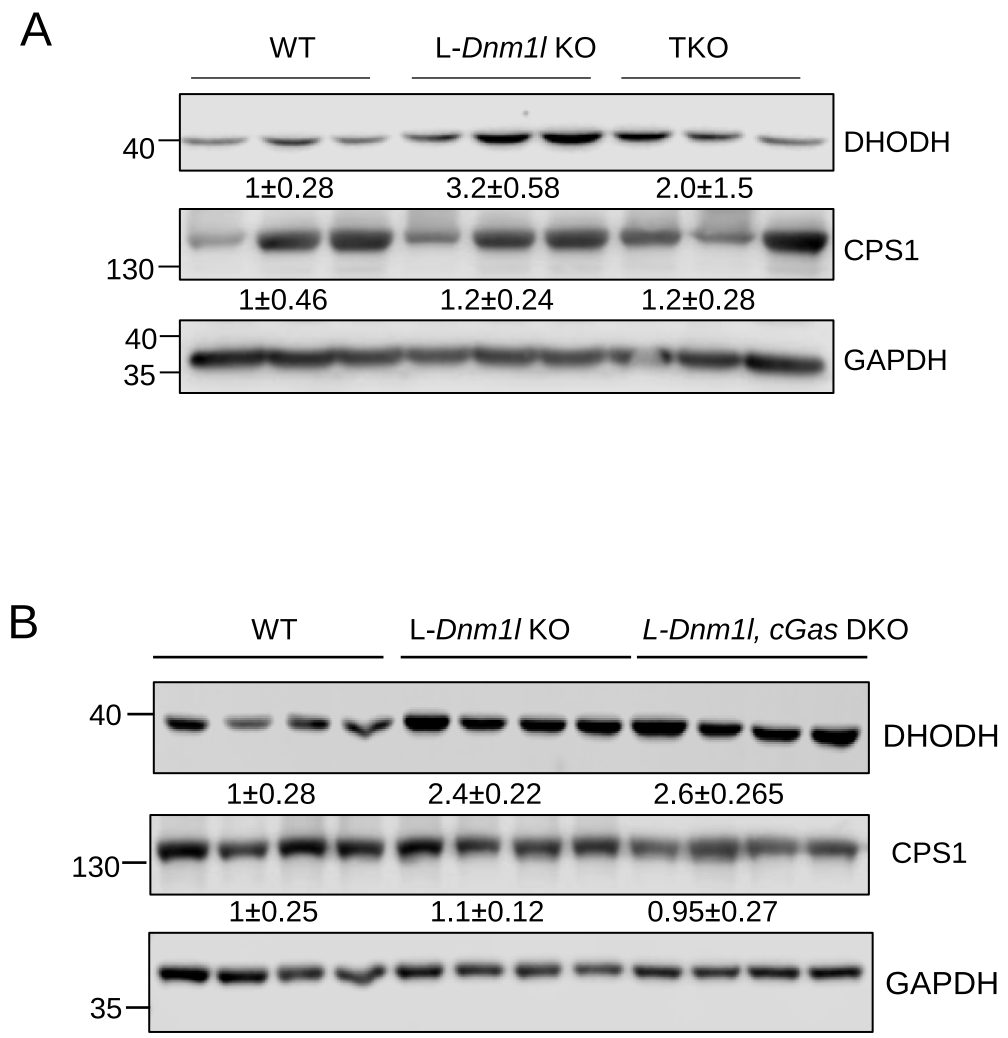

Supplemental Figure 13

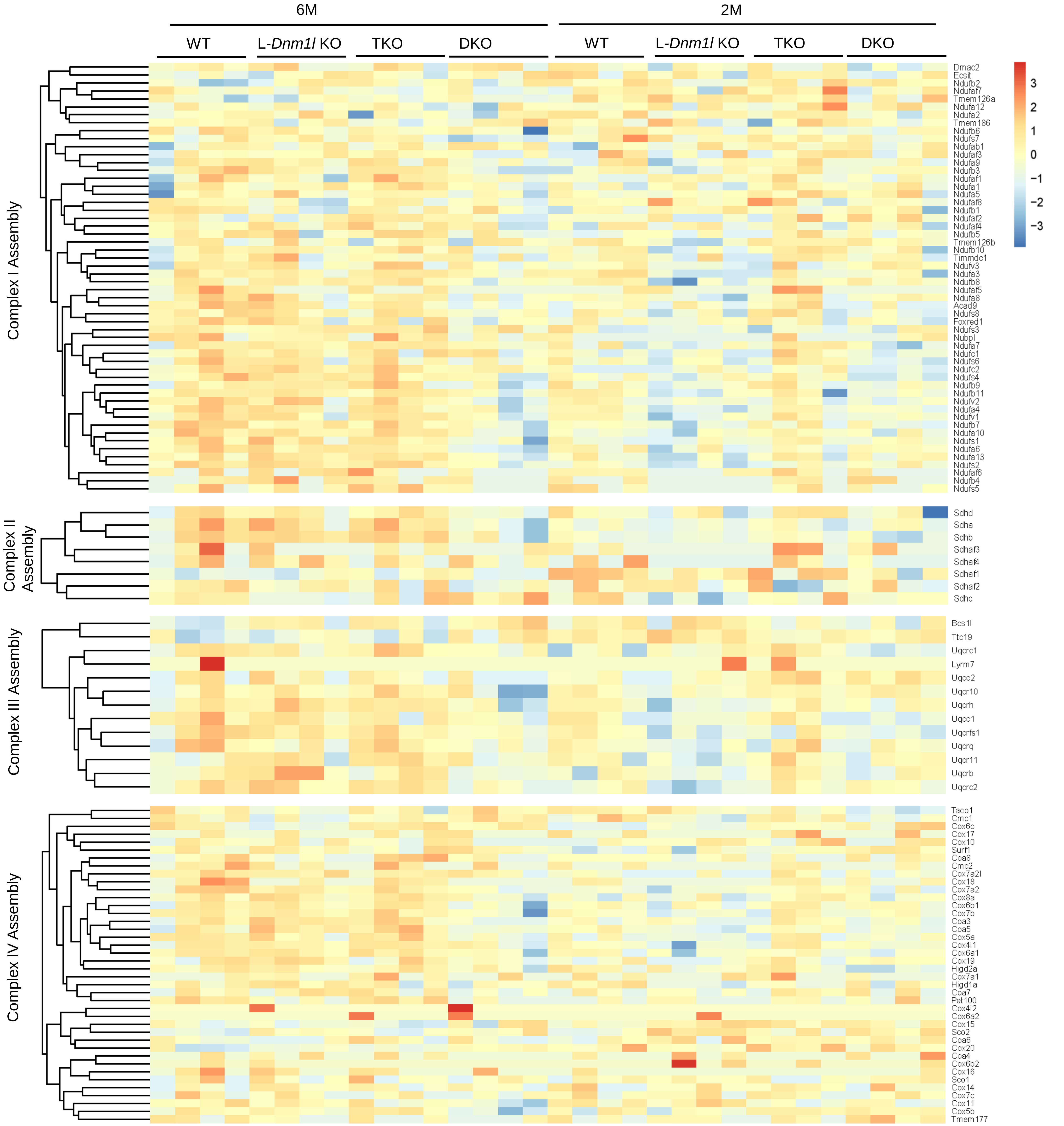

Supplemental Figure 14

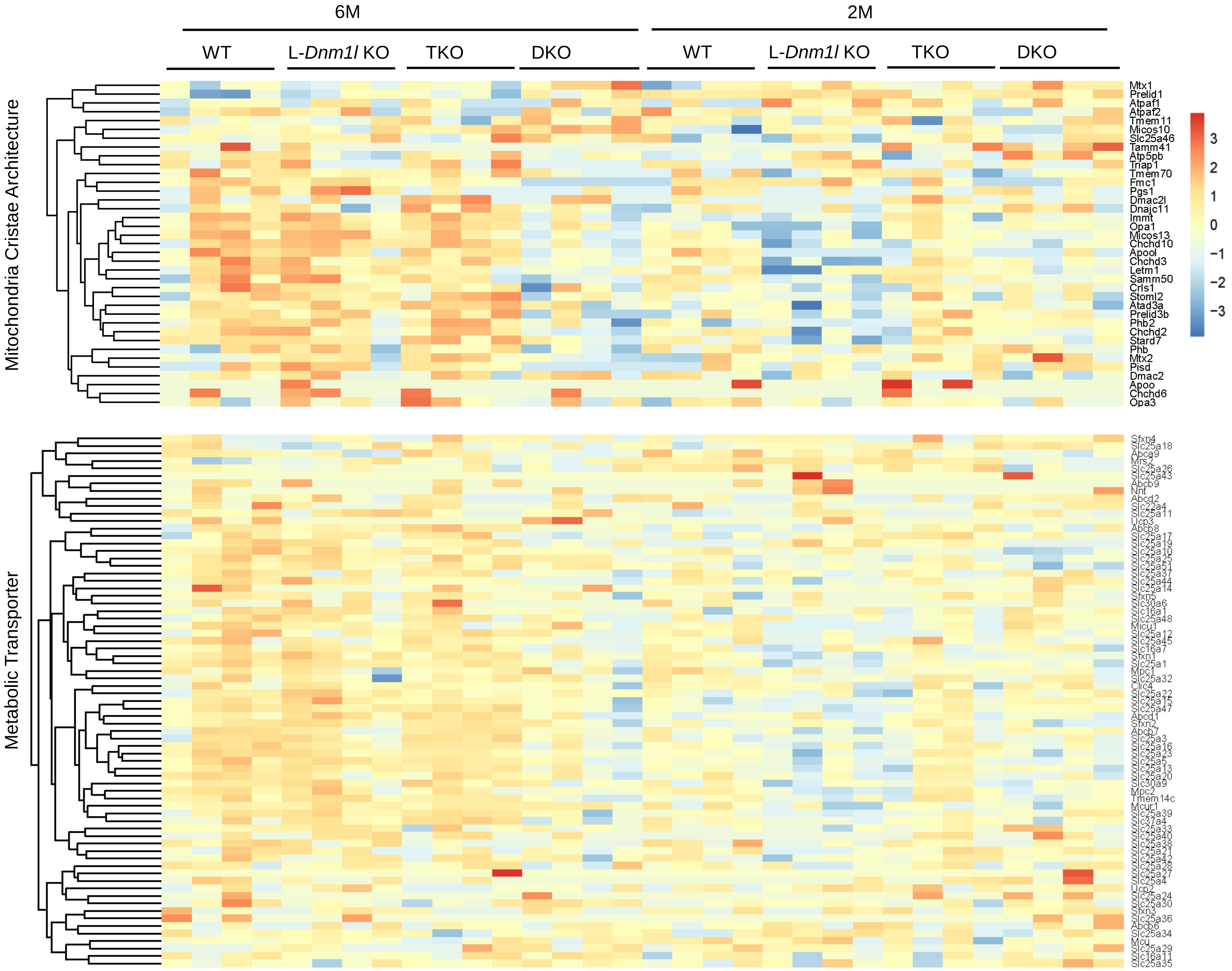

Supplemental Figure 15

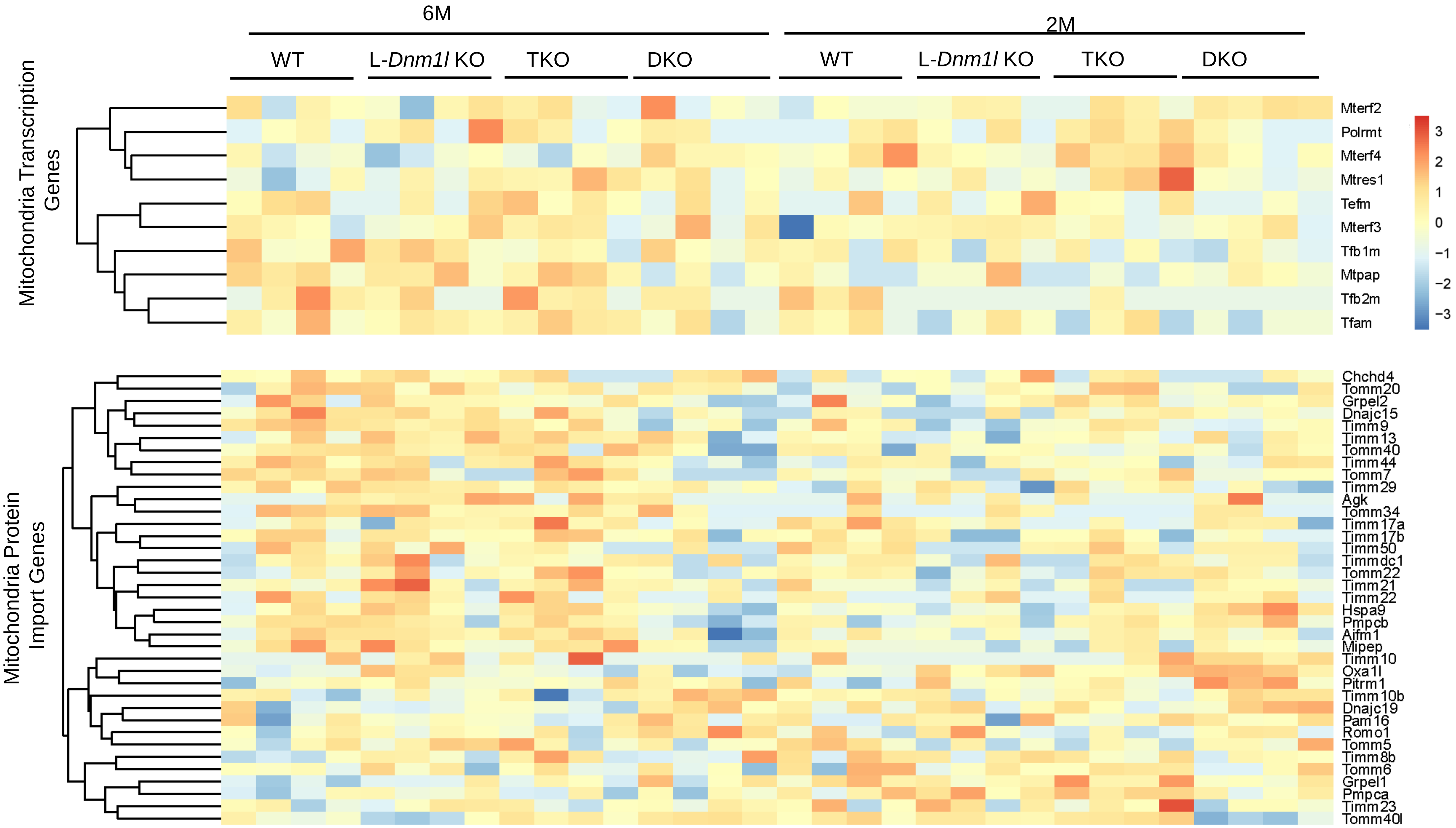

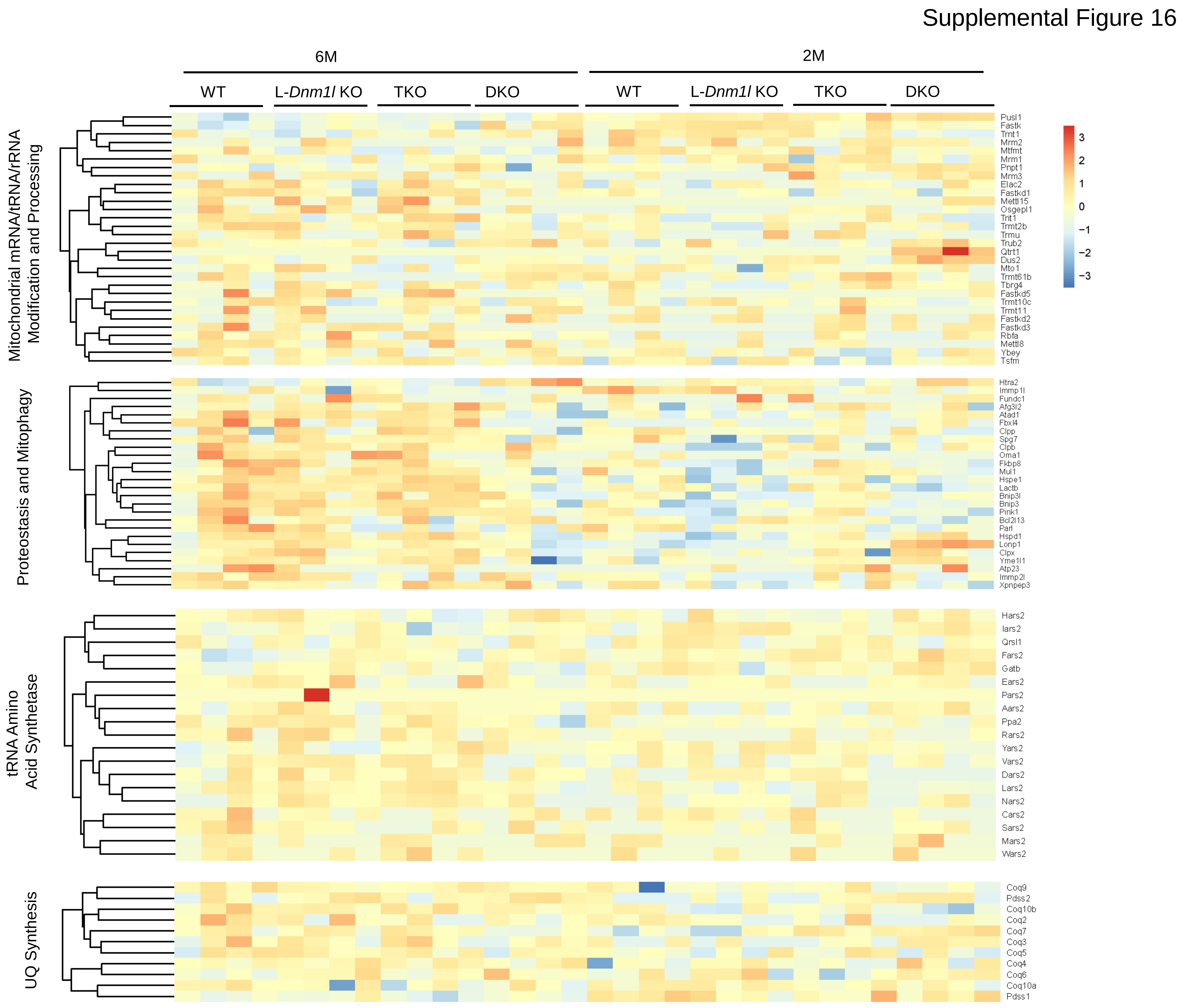
Supplemental Figure 16
6M
2M
WT
L-Dnm1l KO
TKO
DKO
Mitochondrial mRNA/tRNA/rRNA Modification and Processing
Proteostasis and Mitophagy
tRNA Amino Acid Synthetase
UQ Synthesis
Pusl1
Fastk
Trnt1
Mrm2
Mtfmt
Mrm1
Pnpt1
Mrm3
Elac2
Fastkd1
Mettl15
Osgepl1
Trit1
Trmt2b
Trmu
Trub2
Qtrt1
Dus2
Mto1
Trmt61b
Tbrg4
Fastkd5
Trmt10c
Trmt11
Fastkd2
Fastkd3
Rbfa
Mettl8
Ybey
Tsfm
Htra2
Immp1l
Fundc1
Afg3l2
Atad1
Fbxl4
Clpp
Spg7
Clpb
Oma1
Fkbp8
Mul1
Hspe1
Lactb
Bnip3l
Bnip3
Pink1
Bcl2l13
Parl
Hspd1
Lonp1
Clpx
Yme1l1
Atp23
Immp2l
Xpnpep3
Hars2
Iars2
Qrsl1
Fars2
Gatb
Ears2
Pars2
Aars2
Ppa2
Rars2
Yars2
Vars2
Dars2
Lars2
Nars2
Cars2
Sars2
Mars2
Wars2
Coq9
Pdss2
Coq10b
Coq2
Coq7
Coq3
Coq5
Coq4
Coq6
Coq10a
Pdss1
3
2
1
0
−1
−2
−3

Supplemental Figure 17

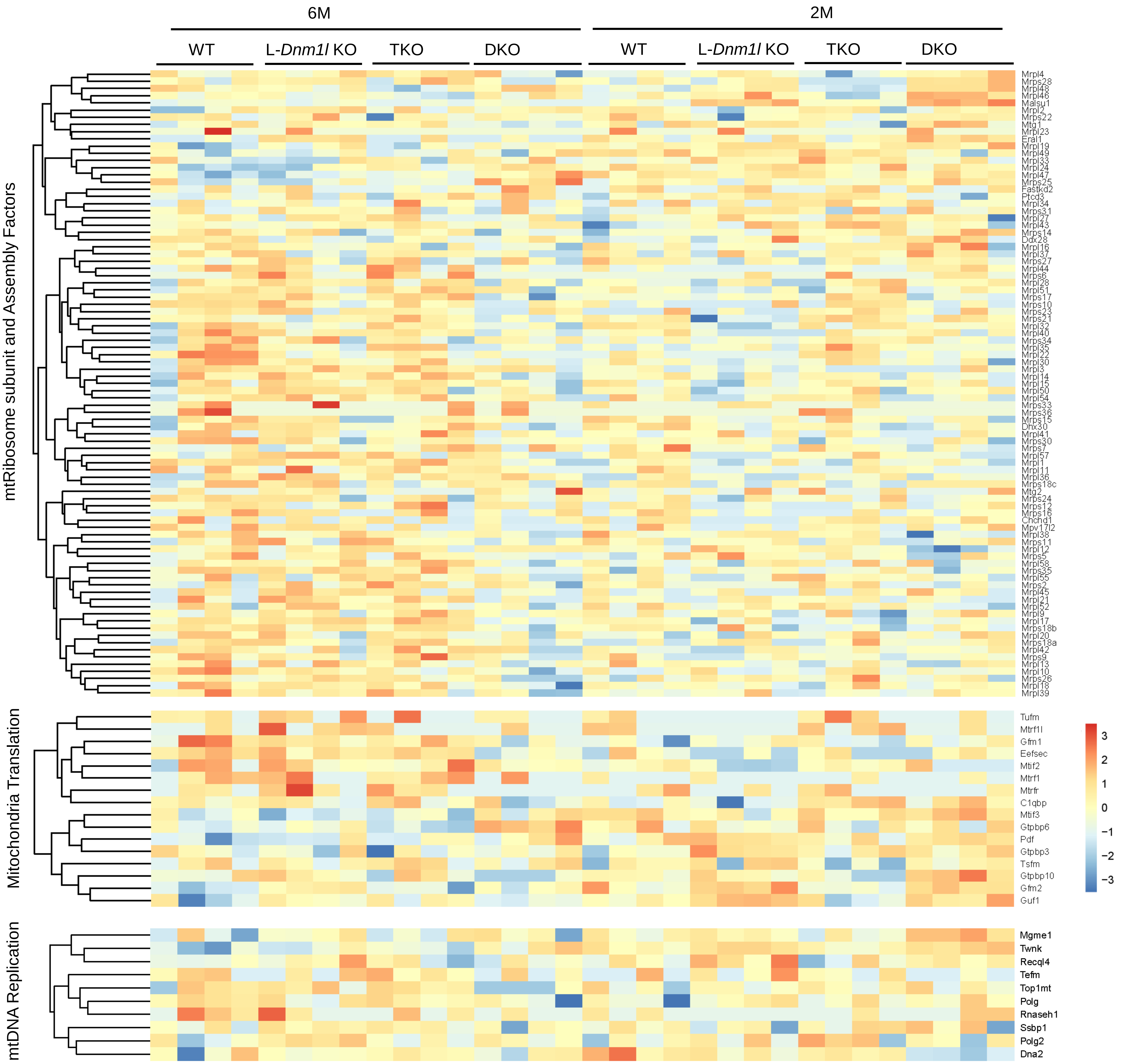

A

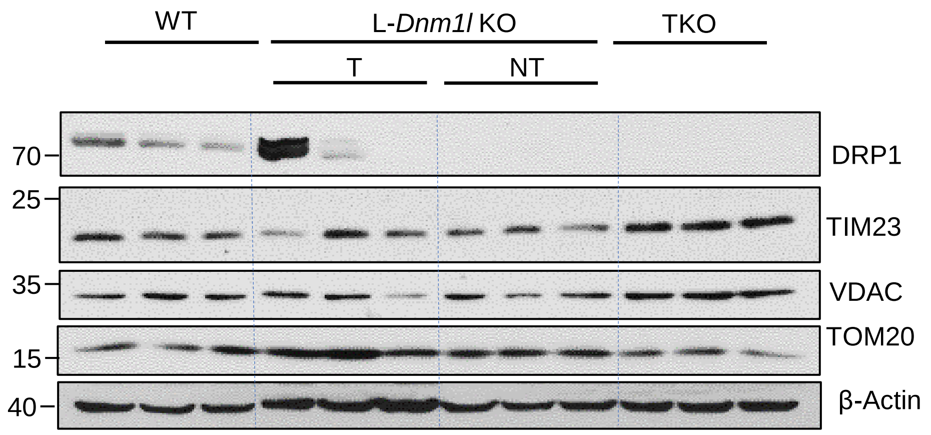
WT
L-Dnm1l KO
TKO
T
NT
70
25
35
15
40
DRP1
TIM23
VDAC
TOM20
β-Actin

B

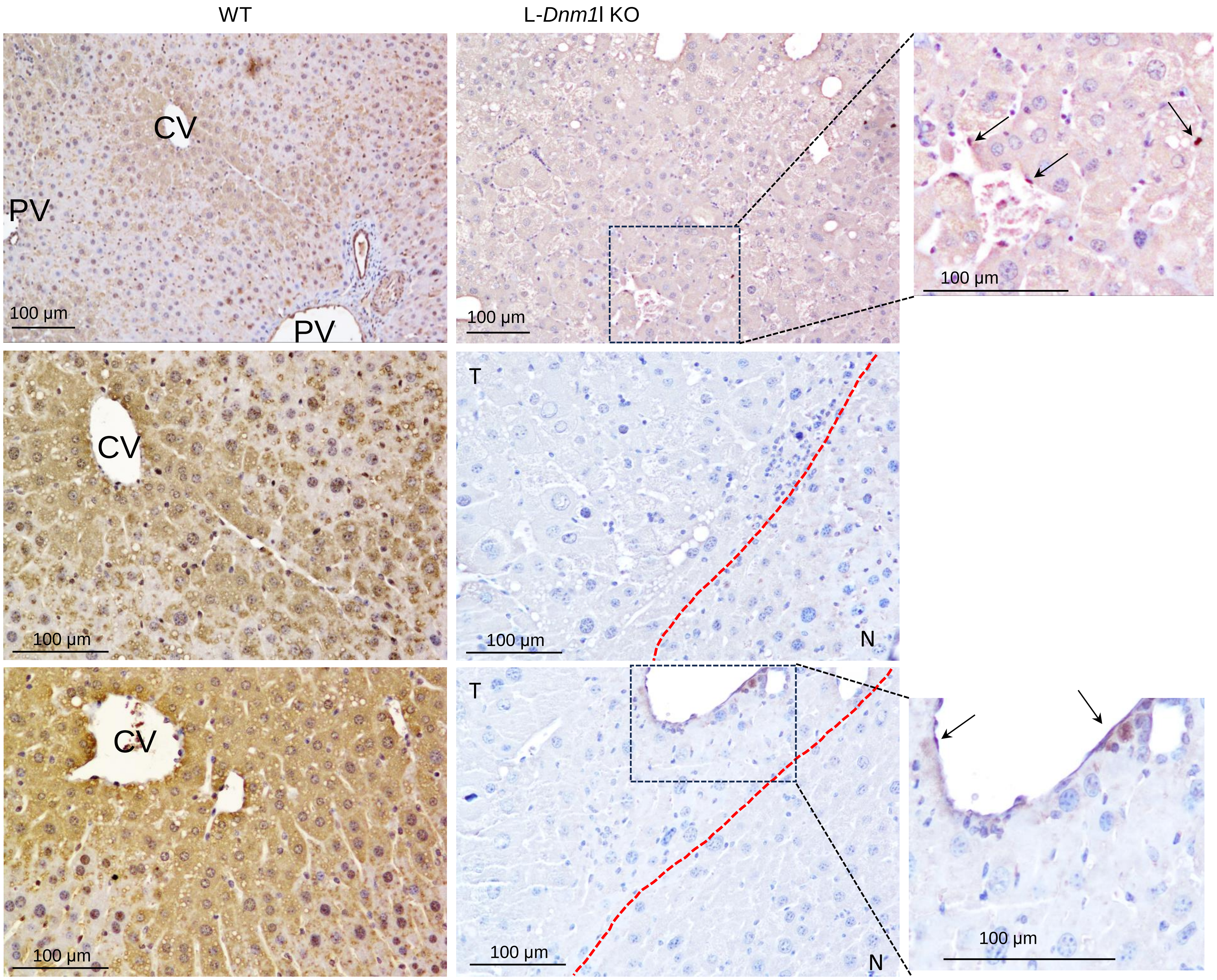
WT
L-Dnm1l KO
CV
PV
PV
100 µm
100 µm
100 µm
CV
100 µm
T
N
100 µm
CV
100 µm
T
N
100 µm
100 µm

Supplemental Figure 19

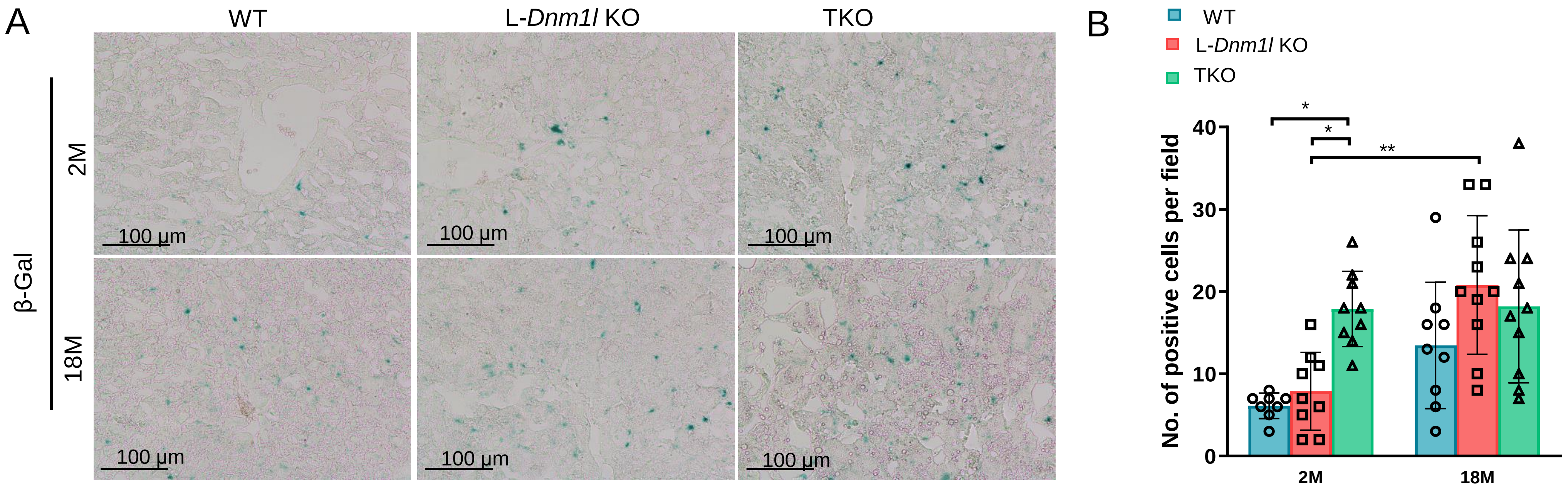

Supplemental Figure 20

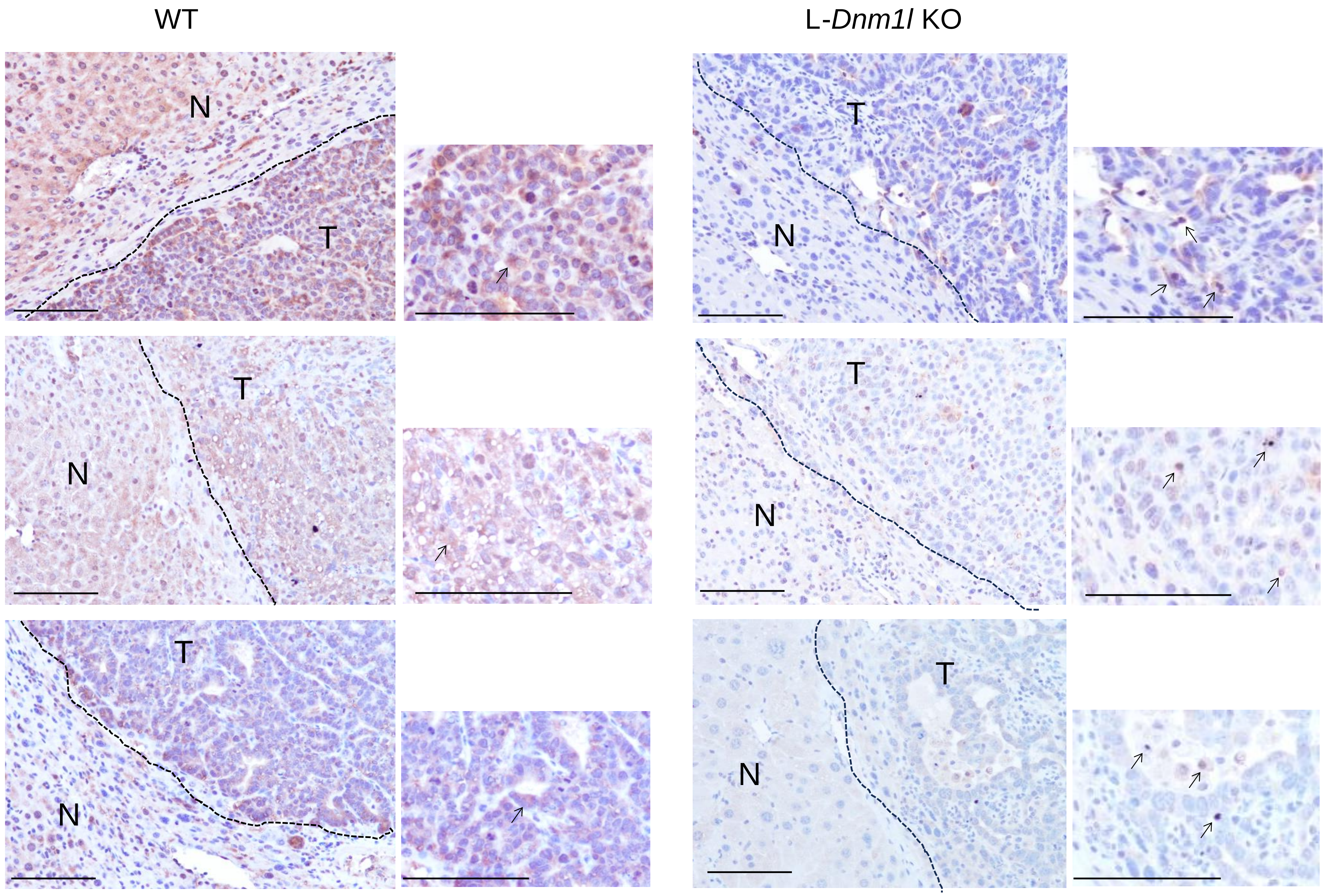

Supplemental Figure 21

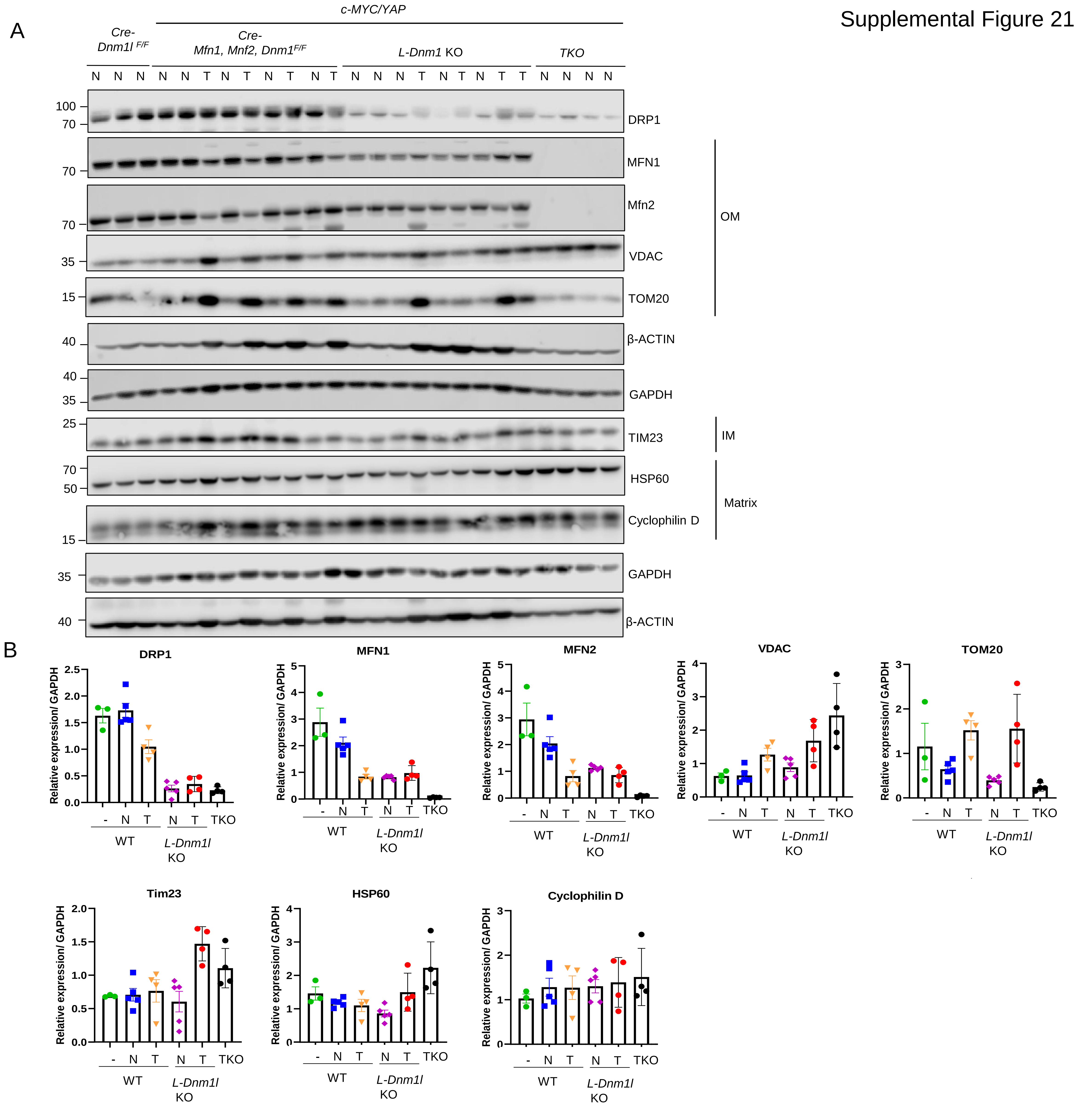

Supplemental Figure 22

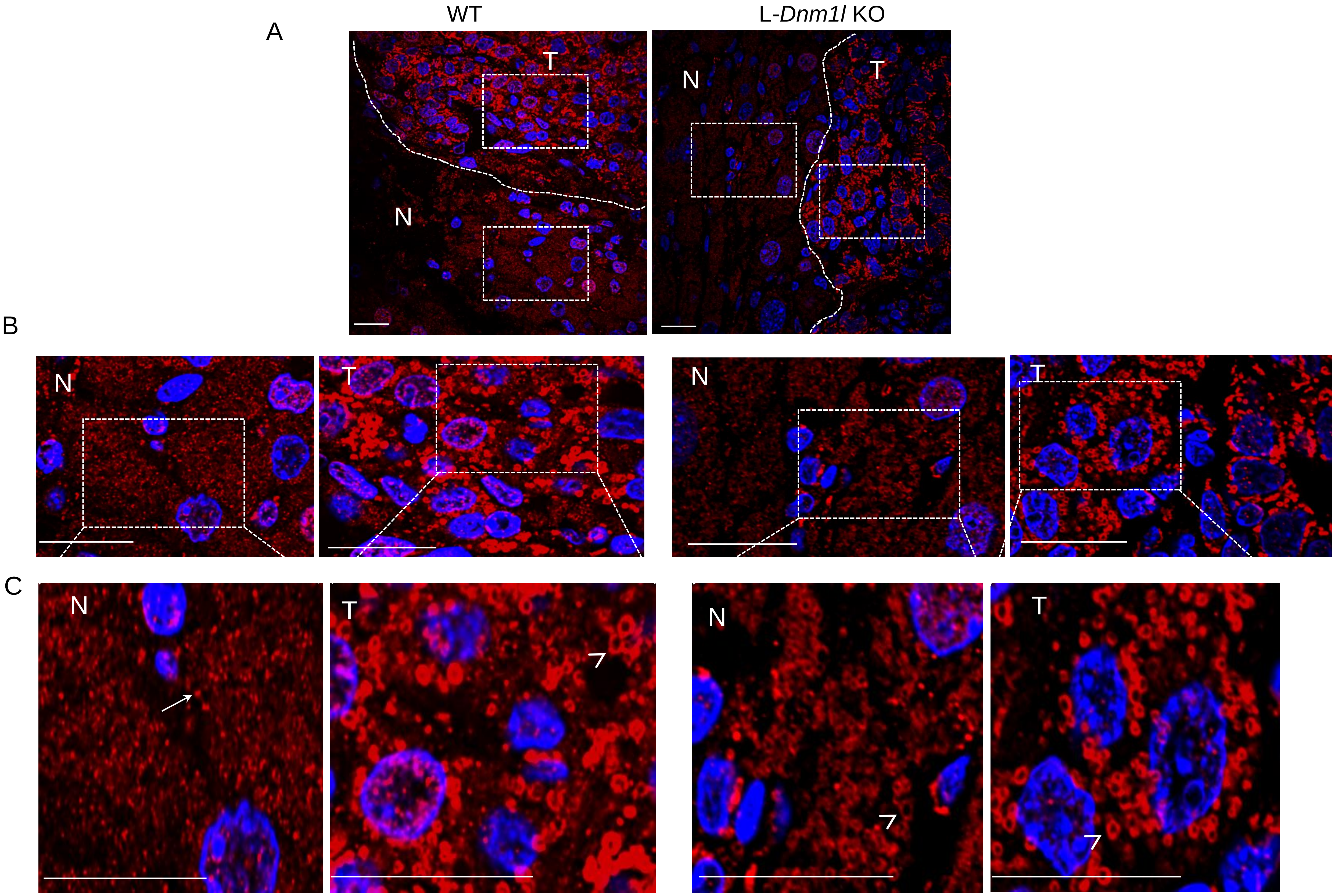

Supplemental Figure 23

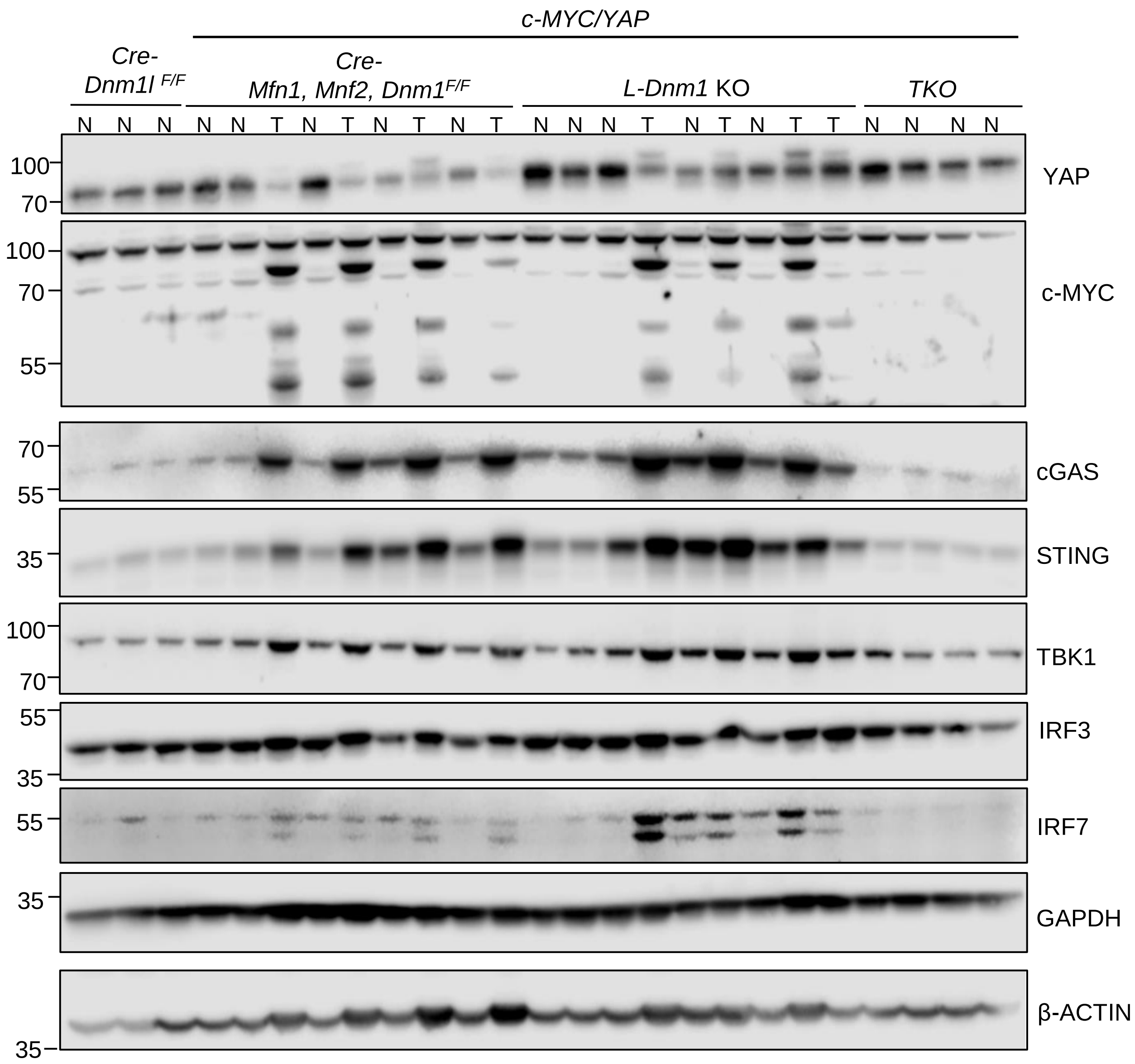

Supplemental Figure 24

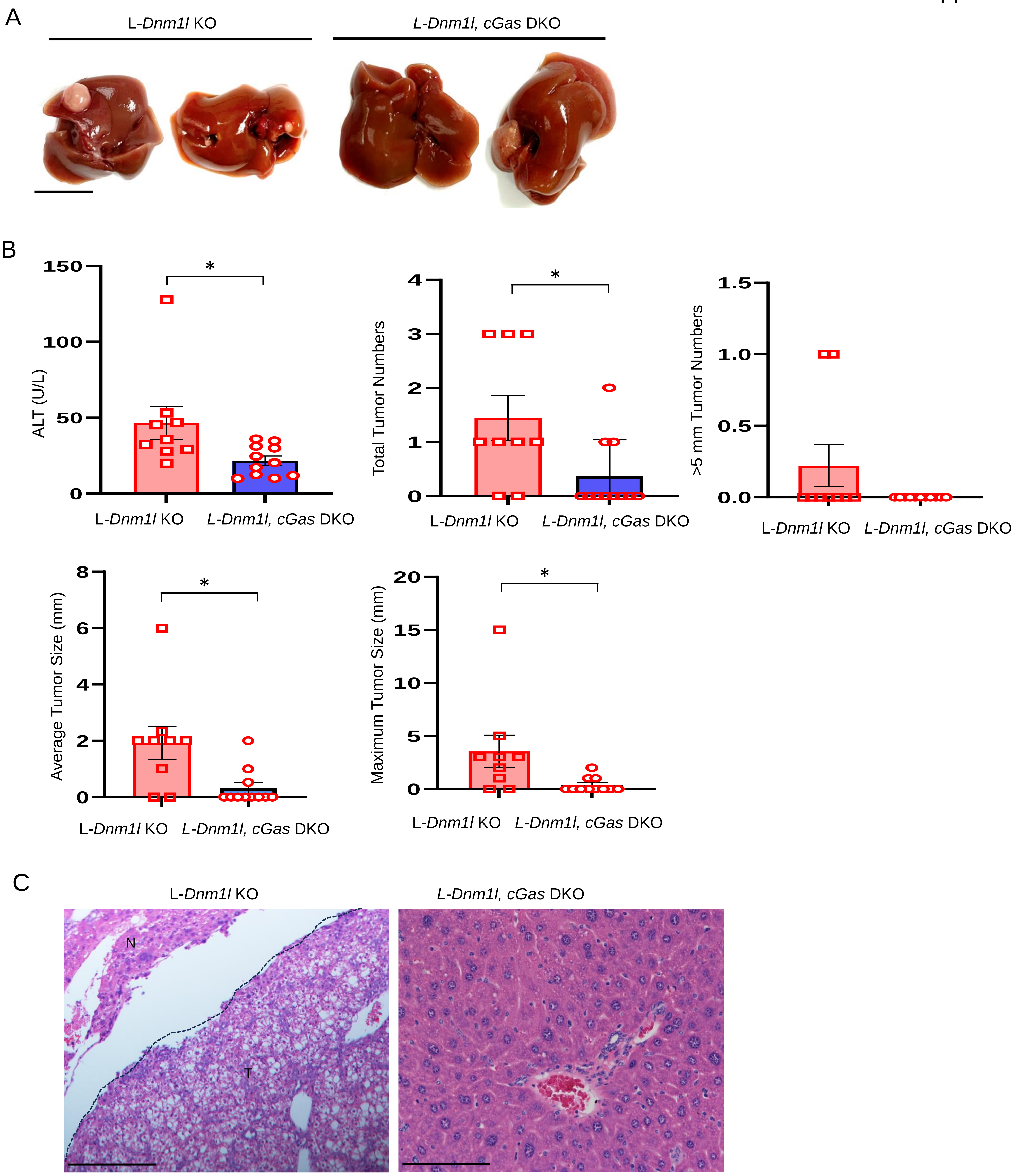

Table S1. Liver tumorigenesis in indicated genotyped mice.

Mice Genotype	Sex	Tumorigenic mice / total		
		6M	15M	⩾18M
L-*Dnm1l* KO	M	0/11	5/6	17/20
	F	0/11	4/8	1/5
L-*Dnm1l, cGAS* DKO	M	0/10	2/7	3/11
	F	0/7	1/6	0/6
