# Supplementary material for: Genetic disruption of mitochondrial dynamics and stasis leads to liver injury and tumorigenesis
Source: J Clin Invest. 2025 Dec 16;136(4):e194441. doi: 10.1172/JCI194441 (PMC12904713; doi:10.1172/JCI194441)

Fig 6B

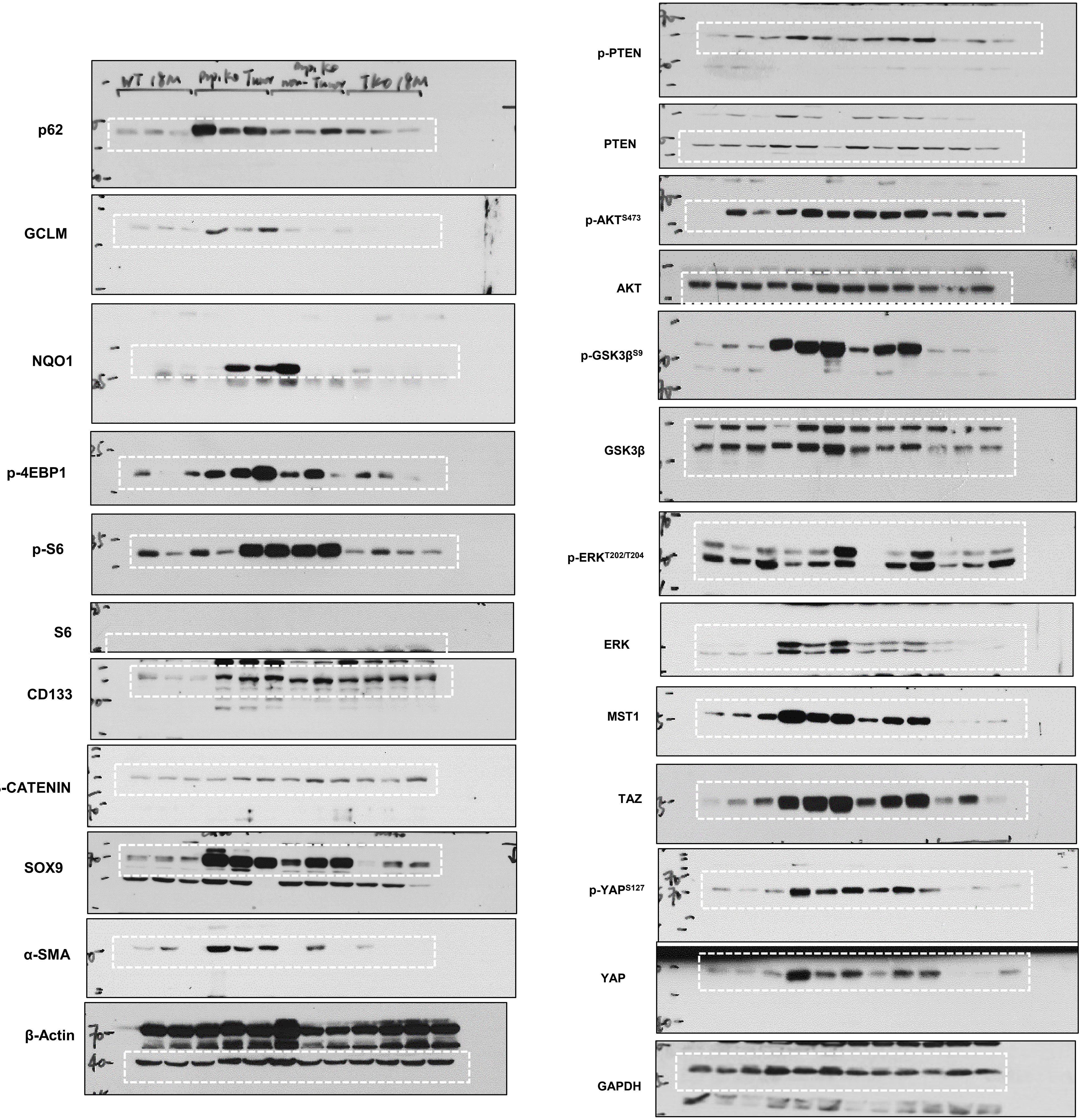

WT 18M
TKO 18M
p62
GCLM
NQO1
p-4EBP1
p-S6
S6
CD133
-CATENIN
SOX9
α-SMA
β-Actin
p-PTEN
PTEN
p-AKT^{S473}
AKT
p-GSK3β^{S9}
GSK3β
p-ERK$^{T202/T204}$
ERK
MST1
TAZ
p-YAP^{S127}
YAP
GAPDH

Figure 7D

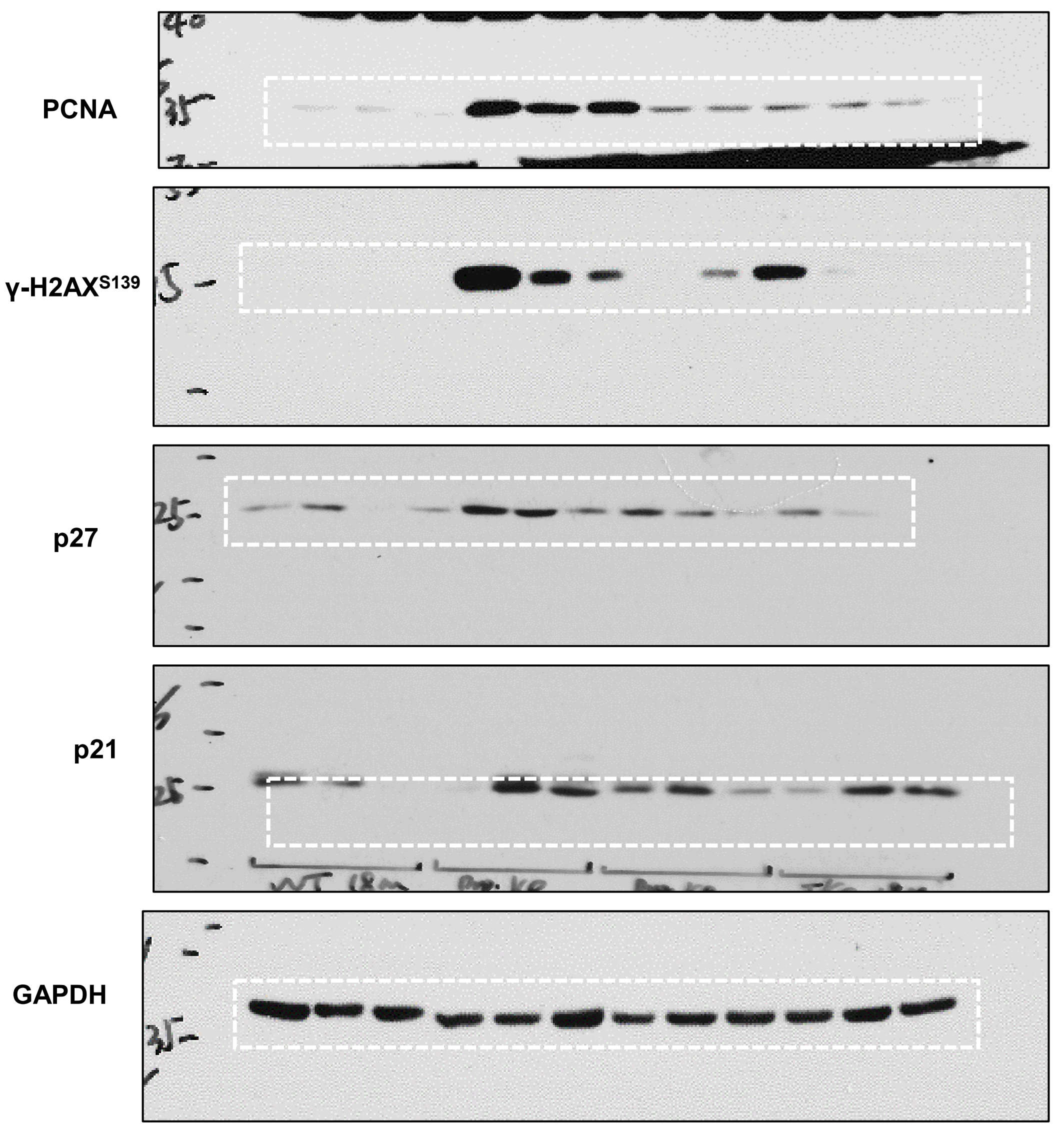

Supplemental Figure 3

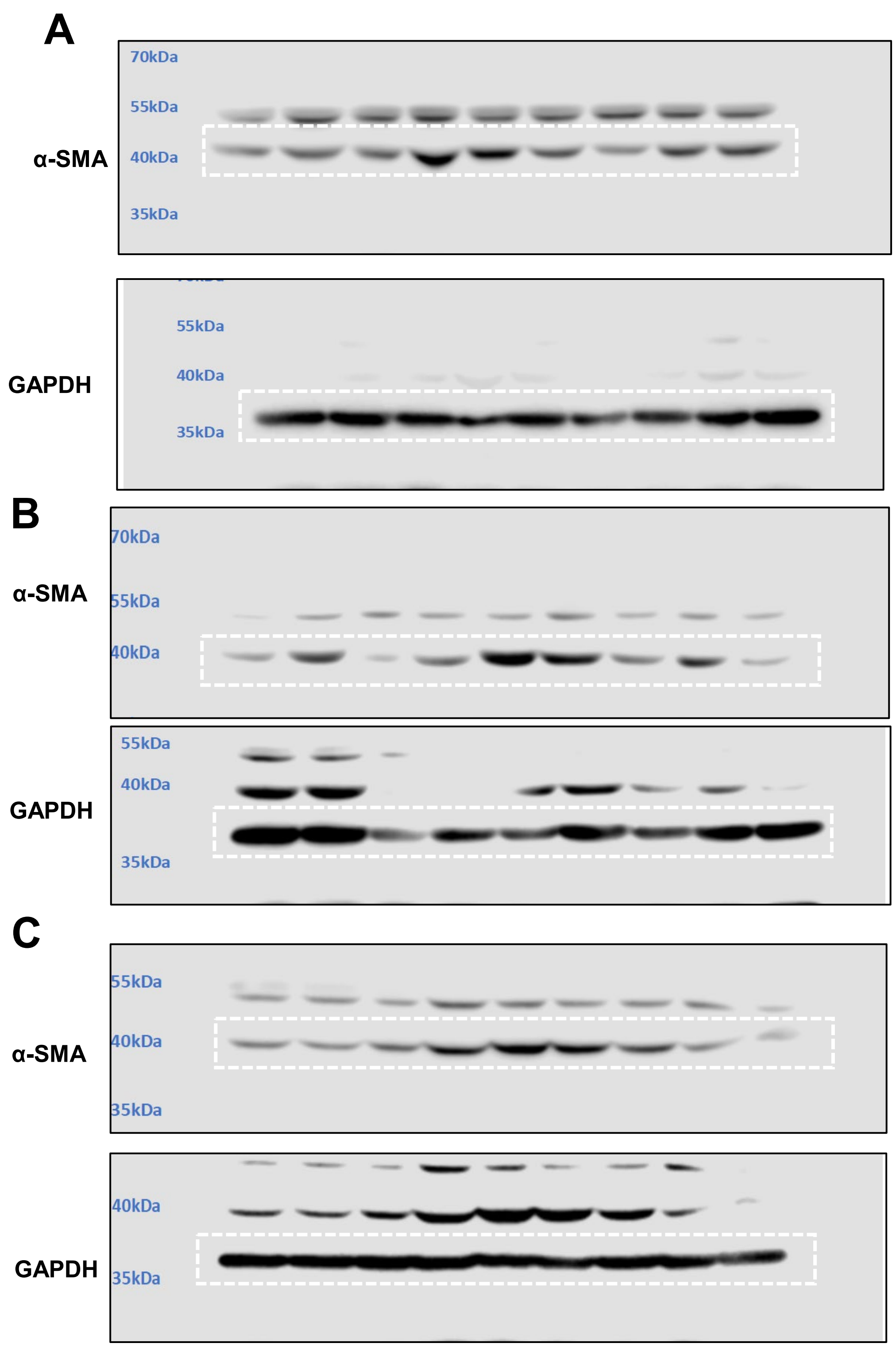

Supplemental Figure 6A

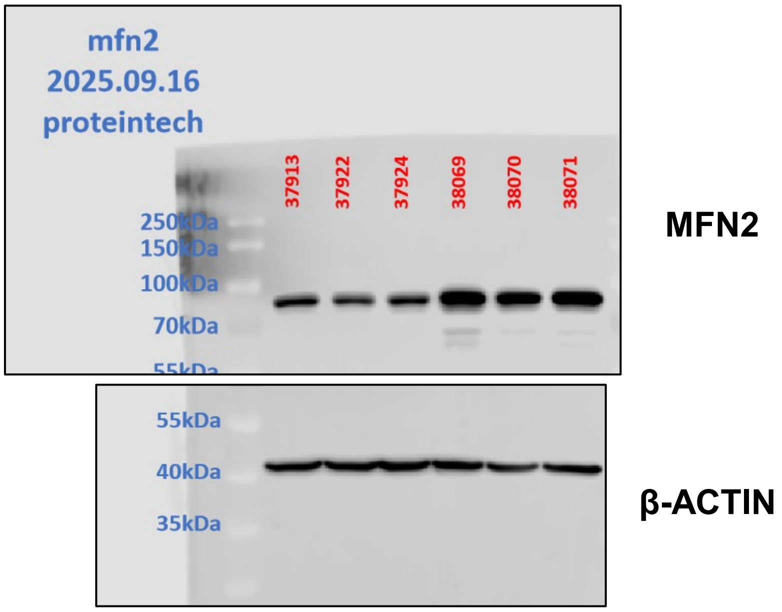

Supplemental Figure 7F

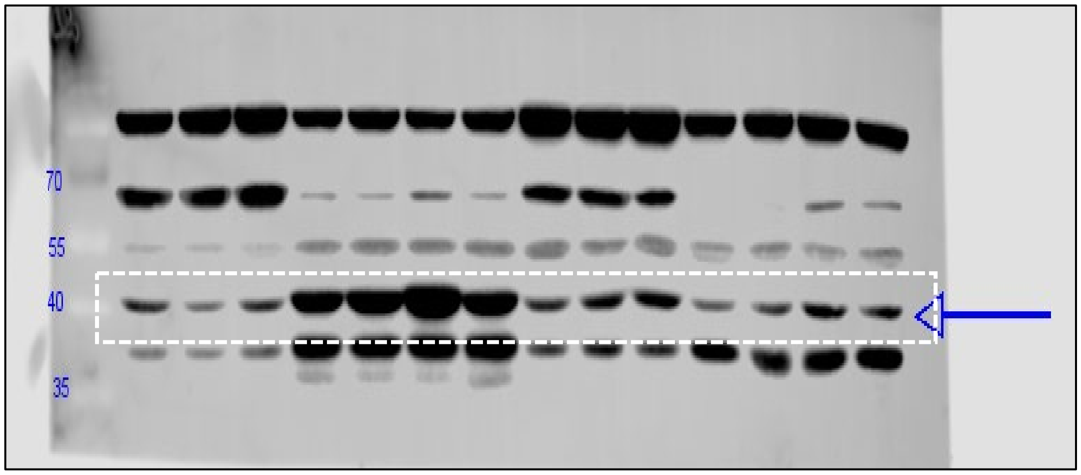

α-SMA

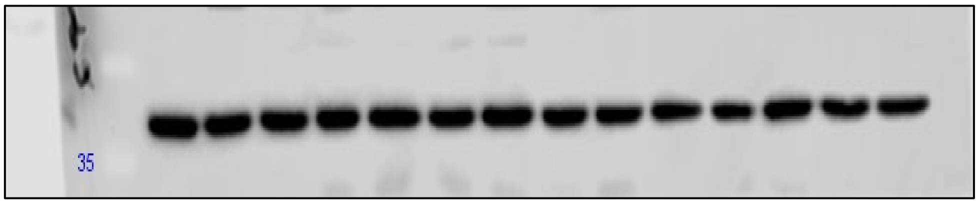

GAPDH

Supplemental Figure 8

C

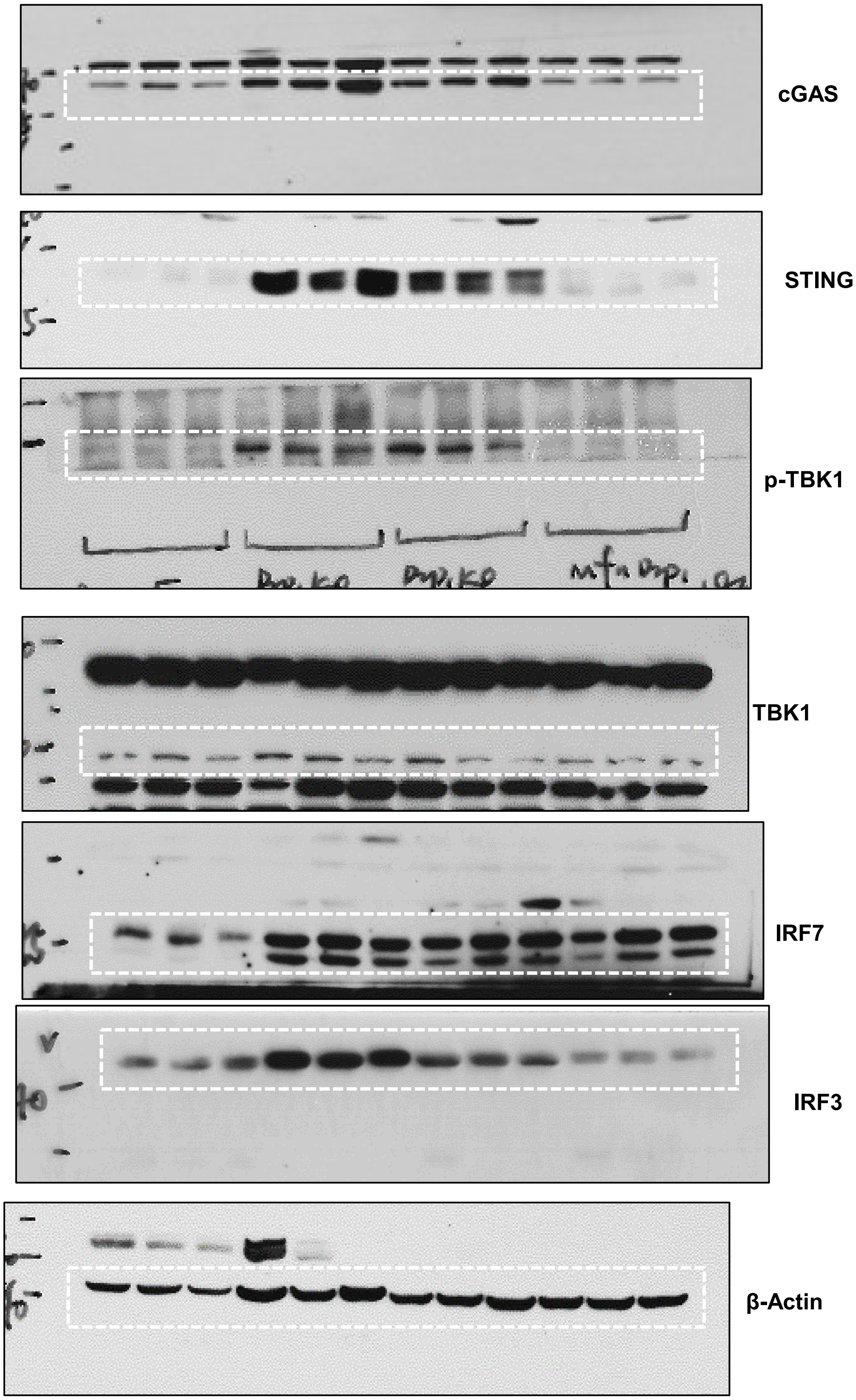

D

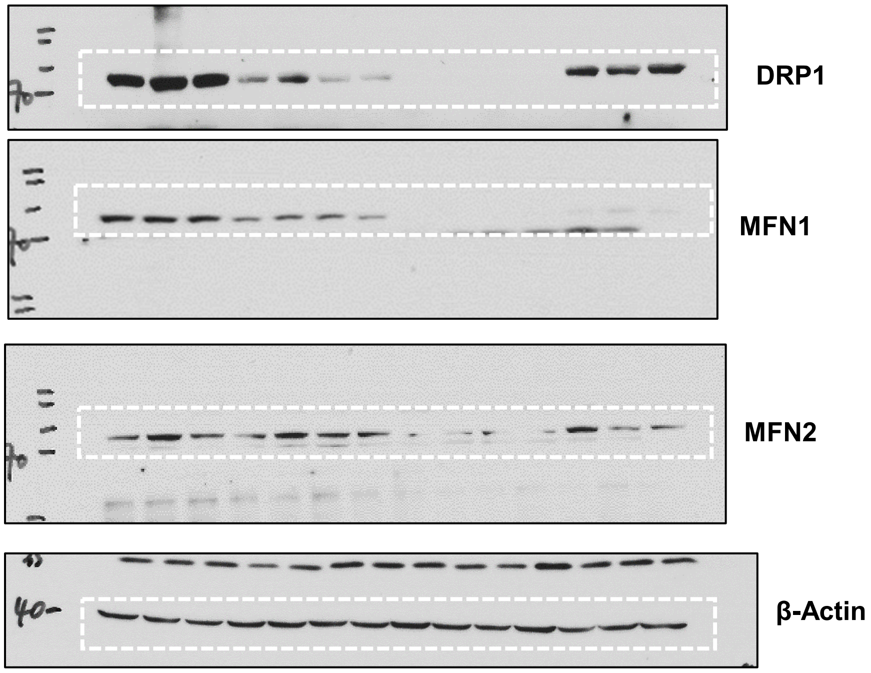

C

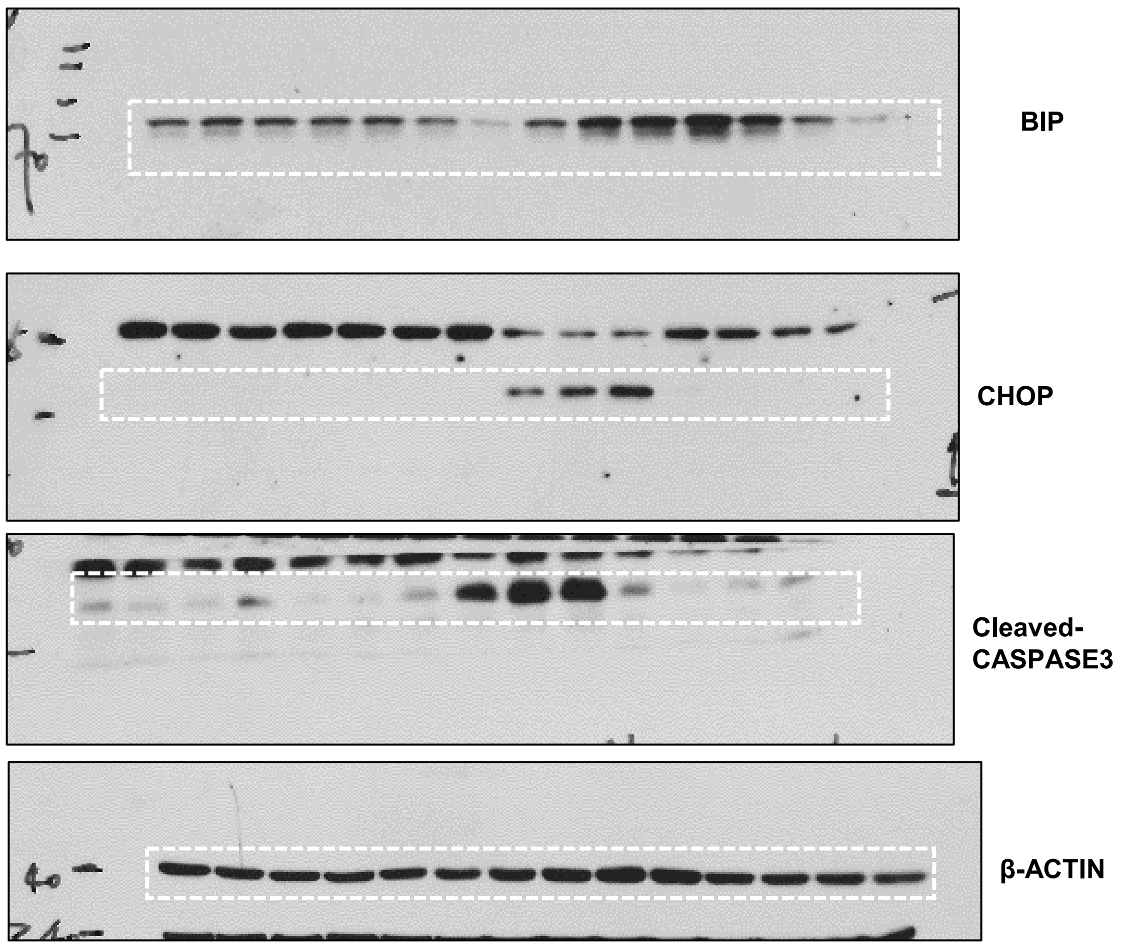

Supplemental Figure 12

A

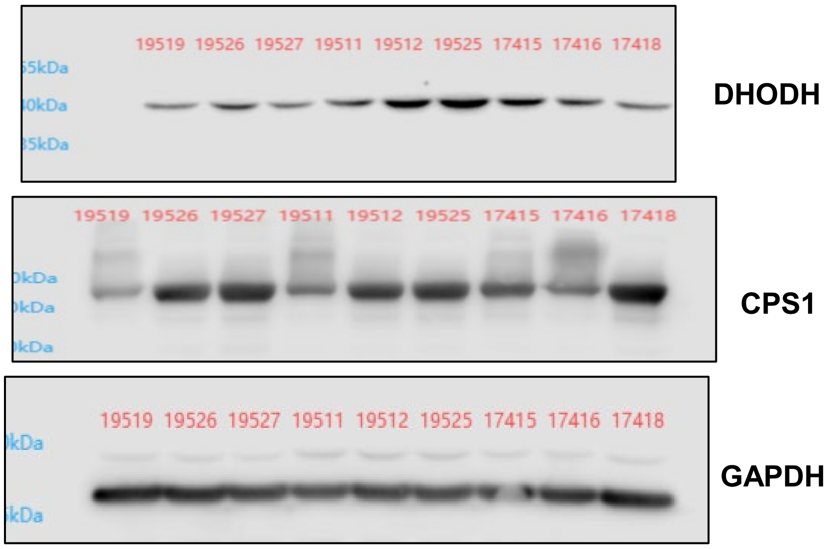

B

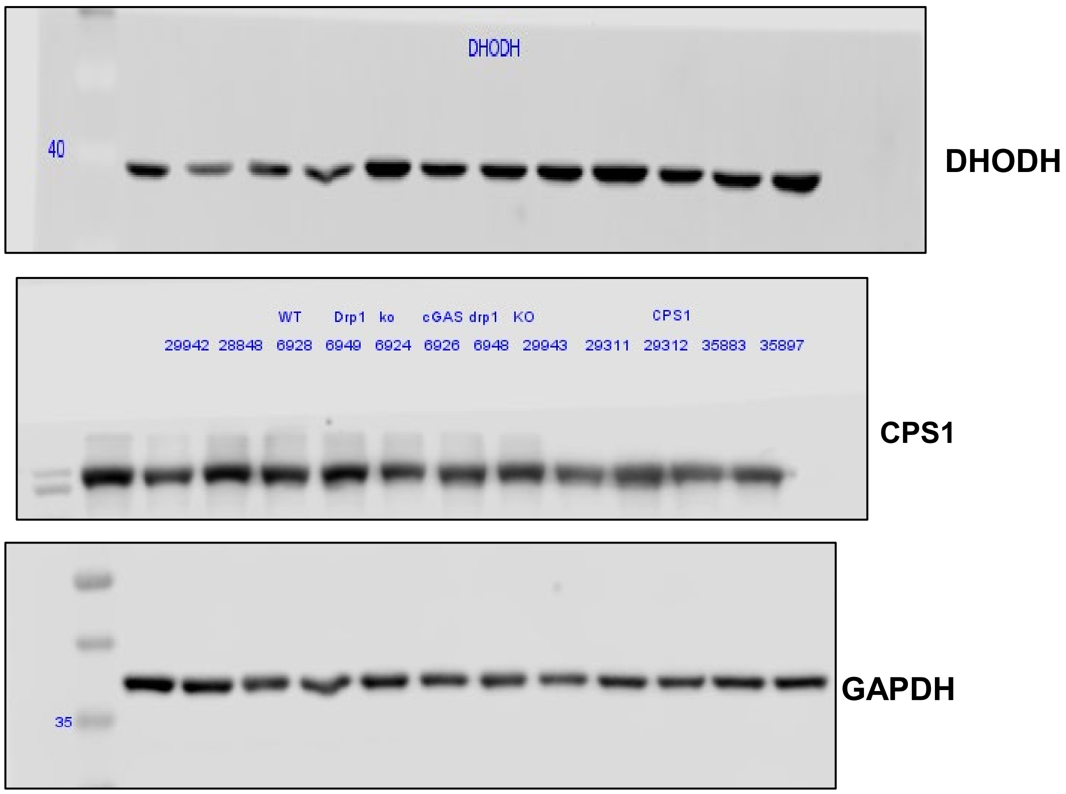

Supplemental Figure 18

A

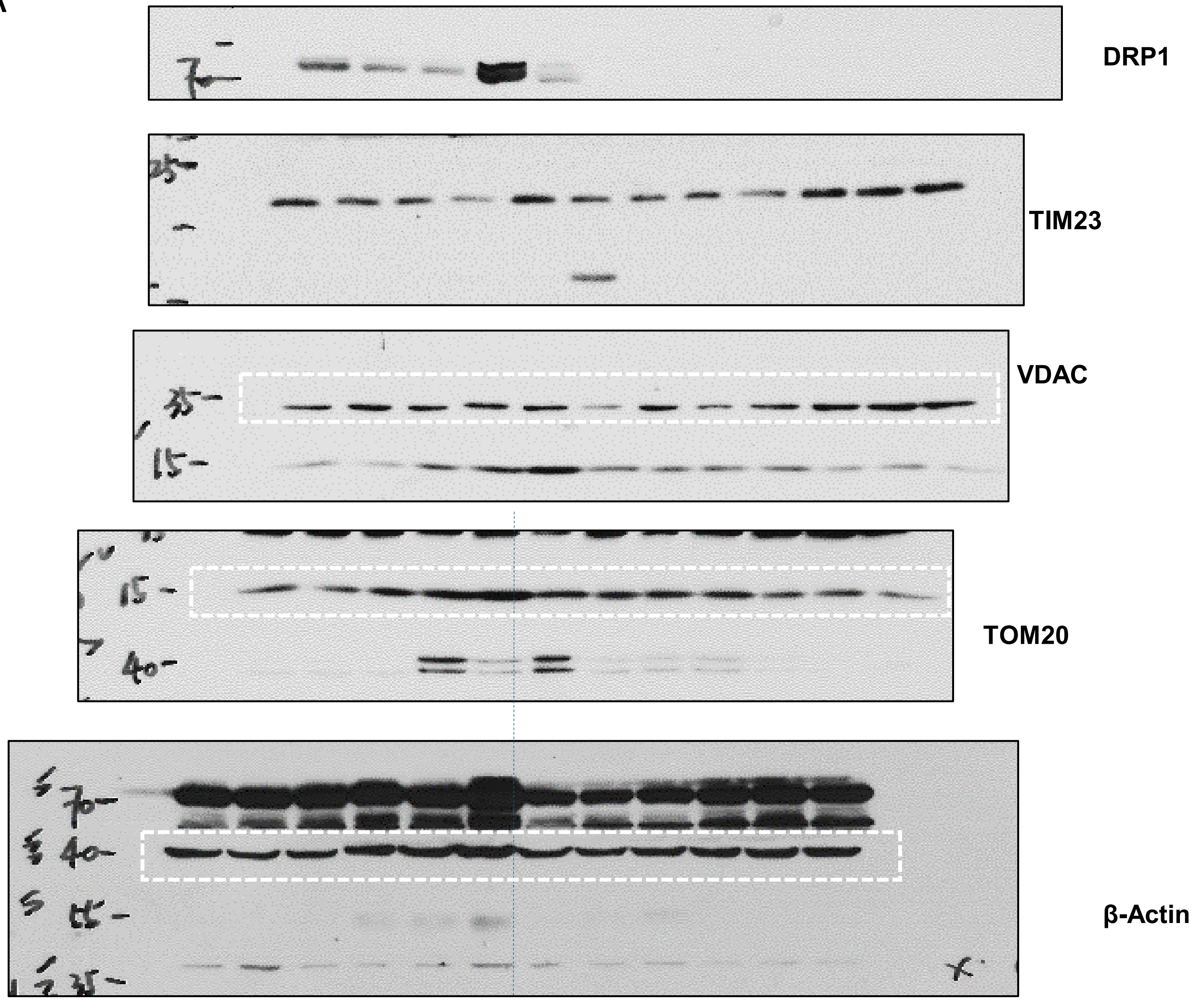

Supplemental Figure 21

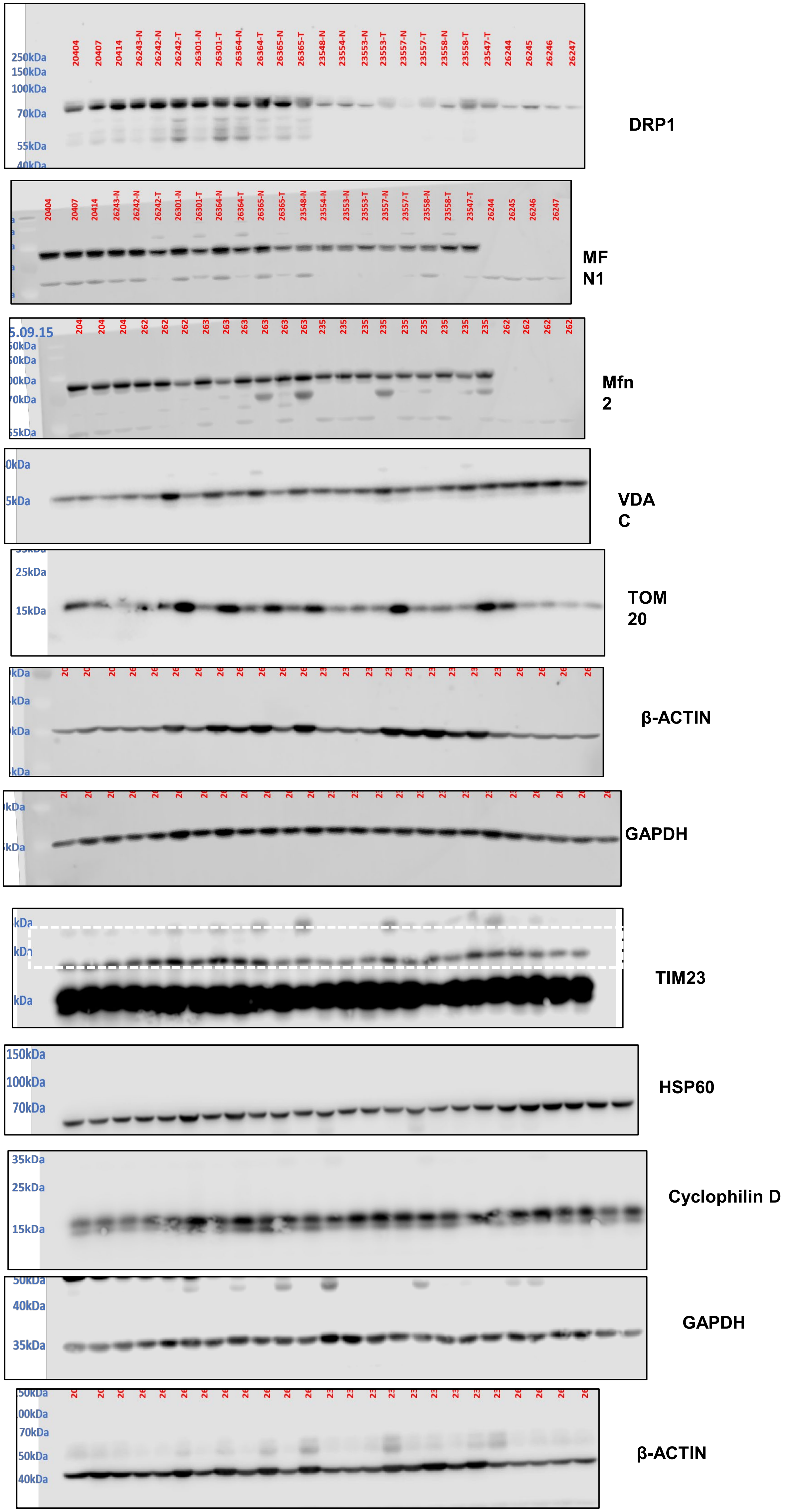

Supplemental Figure 23

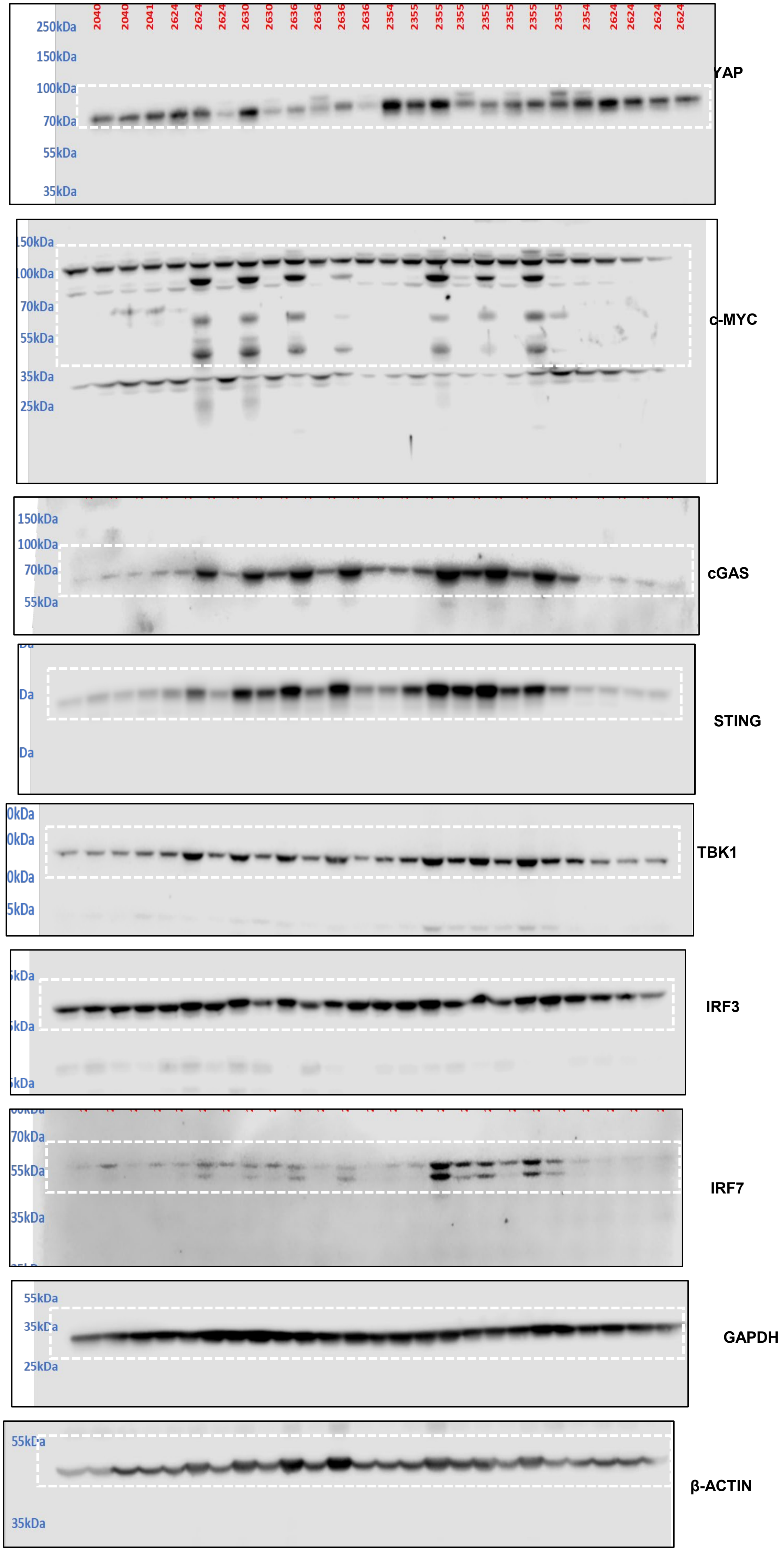

Supplement: Unedited blot and gel images [file jci-136-194441-s196.pdf]
